# Folate deficiency drives mitotic missegregation of the human *FRAXA* locus

**DOI:** 10.1073/pnas.1808377115

**Published:** 2018-12-03

**Authors:** Victoria A. Bjerregaard, Lorenza Garribba, Cynthia T. McMurray, Ian D. Hickson, Ying Liu

**Affiliations:** ^a^Center for Chromosome Stability, Department of Cellular and Molecular Medicine, University of Copenhagen, 2200 Copenhagen N, Denmark;; ^b^Molecular Biophysics and Integrated Bioimaging Division, Lawrence Berkeley National Laboratory, Berkeley, CA 94720;; ^c^Center for Healthy Aging, Department of Cellular and Molecular Medicine, University of Copenhagen, 2200 Copenhagen N, Denmark

**Keywords:** CGG trinucleotide repeats, chromosome missegregation, folate deficiency, FRAXA, RPA UFB

## Abstract

The instability of chromosome fragile sites is implicated as a causative factor in several human diseases, including cancer [for common fragile sites (CFSs)] and neurological disorders [for rare fragile sites (RFSs)]. Previous studies have indicated that problems arising during DNA replication are the underlying source of this instability. Although the role of replication stress in promoting instability at CFSs is well documented, much less is known about how the fragility of RFSs arises. Many RFSs, as exemplified by expansion of a CGG trinucleotide repeat sequence in the fragile X syndrome-associated *FRAXA* locus, exhibit fragility in response to folate deficiency or other forms of “folate stress.” We hypothesized that such folate stress, through disturbing the replication program within the pathologically expanded repeats within *FRAXA*, would lead to mitotic abnormalities that exacerbate locus instability. Here, we show that folate stress leads to a dramatic increase in missegregation of *FRAXA* coupled with the formation of single-stranded DNA bridges in anaphase and micronuclei that contain the *FRAXA* locus. Moreover, chromosome X aneuploidy is seen when these cells are exposed to folate deficiency for an extended period. We propose that problematic *FRAXA* replication during interphase leads to a failure to disjoin the sister chromatids during anaphase. This generates further instability not only at *FRAXA* itself but also of chromosome X. These data have wider implications for the effects of folate deficiency on chromosome instability in human cells.

## Significance

Dietary folate deficiency is associated with fetal neural tube defects, psychological disorders, and age-associated dementia. However, it remains unclear how folate deficiency could be a causative factor in such a diverse range of disorders. Through analysis of the *FRAXA* locus, which contains an extensive CGG repeat sequence, we show that folate deprivation triggers extensive mitotic missegregation of the locus. Moreover, the entire chromosome X becomes unstable during a period of long-term folate deprivation. Considering that the human genome contains several loci associated with extensive CGG repeat regions, these findings suggest a mechanism by which folate deficiency contributes to the onset of a wide range of human diseases.

A chromosome fragile site is a locus that is prone to form a gap or break in an otherwise condensed metaphase chromosome. Based on their prevalence, these loci are defined as being either common fragile sites (CFSs), which are present in all individuals, or rare fragile sites (RFSs), which exist in less than 5% of the population. CFS and RFS instability can be induced by exposure of cells to agents that interfere with DNA metabolism; however, the inducing agent in each case differs. CFSs are classified as being aphidicolin (APH)-inducible, bromodeoxyuridine (BrdU)-inducible, or 5-azacytidine−inducible, while RFSs are classified as being folate-inducible or non−folate-inducible (1). While CFS instability is recognized as a driver of genome instability in cancers (2), many RFSs are associated with intellectual disability disorders (3). To date, much of our understanding of the underlying cause of fragile site instability has been derived from studies of CFSs.

CFS fragility is induced by conditions that create so-called DNA replication stress. It is generally considered that CFS “expression,” defined as the presence of a visible gap/break on a metaphase chromosome, results from a localized inability to properly condense the DNA during early mitosis due to incomplete DNA replication of the locus during interphase (3, 4). The cause of the replication failure is still debated, but increasing evidence suggests that conflicts arising during attempted replication and transcription of the same DNA template are a key driver. We have demonstrated previously that, under replication stress conditions, CFSs are marked by the presence of the FANCD2 and FANCI proteins irrespective of whether the locus is broken or not. These proteins appear at CFS loci as “twin foci” on the sister chromatids in metaphase spreads (5). Many of these FANCD2 foci persist into anaphase and become interlinked by PICH-associated ultrafine DNA bridges (UFBs) (5, 6). Moreover, CFSs have a propensity to be segregated into a micronucleus at the end of mitosis (5, 7), which is a potential source of further genome instability in the daughter cells.

Folate is an essential vitamin that provides the one-carbon source necessary for DNA synthesis. Because folate cannot be synthesized in the human body, dietary sources are essential, and deficiency is, therefore, widespread in human populations affected by malnutrition or where supplementation with folic acid is lacking. To date, folate deficiency is known to be associated with anemia, fetal neural tube defects, infertility in men and women, a wide range of common cancers, psychological disorders, and age-associated dementia (8–17). Previous analyses have suggested that folate deficiency could lead to the formation of micronuclei, nucleoplasmic bridges, and nuclear buds in human lymphocytes (18). Moreover, folate deficiency drives chromosome instability (e.g., chromosome 21 aneuploidy) (19) and DNA replication-associated DNA breakage (20). Nonetheless, it remains largely mysterious how folate deficiency can cause widespread genome instability in human cells. Interestingly, it is well established that a subgroup of RFSs that are characterized by the presence of CGG trinucleotide repeat (TNR) sequences is particularly susceptible to folate deficiency (1). When these TNR sequences expand beyond a critical size, the development of specific neurological diseases can be triggered; most notably, fragile X syndrome (FXS), the most common inherited form of mental retardation (21–24).

The CGG TNR sequence that becomes pathologically expanded in FXS is located at the *FRAXA* locus on the long arm of chromosome X (ChrX) (at Xq27.3). This TNR lies within the 5′ untranslated region of the fragile X mental retardation 1 gene (*FMR1*) (24). In the healthy population, this repeat ranges in length from 6 to 53 triplets, and this locus is stably transmitted to the next generation (23). When the repeat reaches the premutation range (PM; 55 to 200 repeats), it is then prone to expand to a full mutation (FM; >200 repeats) in the next generation (23). FM cases are accompanied by increased methylation of the promoter region of *FMR1*. This leads to *FMR1* gene silencing and reduced expression of FMRP protein (25), which is the direct cause of the symptoms associated with FXS (26). Furthermore, ChrX aneuploidy has been observed in female carriers (27) and in male FXS patients (28–30), but the mechanism underlying this form of *FRAXA* instability also remains unknown. It has been speculated that an atypical DNA structure formed by the CGG repeat itself, such as a hairpin-like structure (31), quadruplex (32), or R loop (33, 34), could contribute to its instability. Interestingly, repeat-length mosaicism has also been reported in PM and FM fragile X males. In these cases, skin and blood cells tend to have different repeat lengths at *FRAXA*, which is associated with variable expression of FMRP. This suggests the fragile X CGG repeat is unstable in somatic tissue during embryogenesis and perhaps during early development. It is also intriguing that folate-sensitive RFSs have two features that distinguish them from CFSs: (*i*) They are found only associated with long CGG repeats; and (*ii*) they are located at the promoters of genes whose transcription is generally silenced due to the expansion of CGG repeats, indicating that the fragility of RFSs is unlikely to be caused by the collision between the replication fork and an unprocessed transcript, as has been proposed for CFSs.

Against this backdrop, we hypothesized that folate deficiency would specifically affect the replication program in genomic regions containing CGG repeats, and that this would then lead to mitotic abnormalities similar to those observed at CFSs. In this study, we used a panel of lymphocytes derived from males who have a normal, PM, or FM *FRAXA* allele to examine mitosis under folate stress conditions. We demonstrate that folate deficiency leads to a dramatic defect in the segregation of FM *FRAXA* in mitosis. We also reveal that ChrX aneuploidy is observed during extended folate stress in the FM cell line. We propose, therefore, that folate deprivation can trigger chromosome instability due to defective mitotic sister chromatid disjunction of genomic regions containing long CGG repeats.

## Results

### *FRAXA* Exhibits Fragility and Is Missegregated in Mitosis During Folate Stress.

We analyzed a panel of immortalized male human lymphocytes that have normal, PM, or FM *FRAXA* alleles (*SI Appendix*, Fig. S1 *A* and *B*); these cell lines will henceforth be referred to as normal, PM, or FM cells, respectively. To track the location of *FRAXA* in mitosis, we performed fluorescence in situ hybridization (FISH) using DNA probes targeting either the *FRAXA* locus or the ChrX centromere (*ChXCEN*). It was shown previously that disruption of thymidine synthesis induced either by folate deprivation or by treatment with the thymidylate synthetase inhibitor, fluorodeoxyuridine (FdU), could cause the fragility at *FRAXA* in lymphocytes from FM carriers (35). We first confirmed that *FRAXA* fragility could indeed be induced in the FM cell line GM09237, either by exposure of cells to 0.5 µM FdU or by culturing them in the absence of folate for 3 d (*SI Appendix*, Fig. S1 *C*–*F*). As expected, we also observed that neither APH nor hydroxyurea could cause fragility at *FRAXA* in the FM cell line, GM09237 (*SI Appendix*, Fig. S1*E*).

**Fig. 1.**
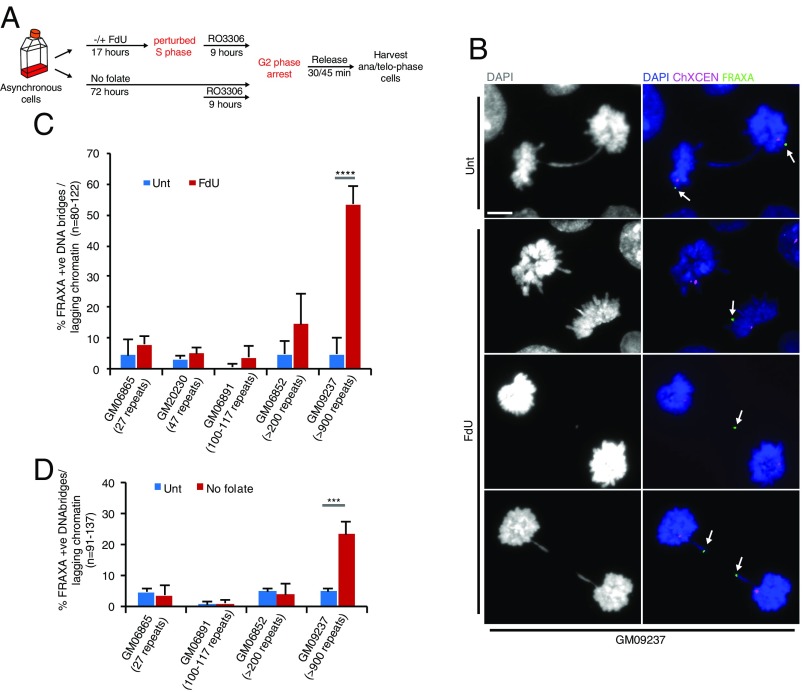
*FRAXA* is located on DNA bridges and lagging chromatin in response to folate stress. (*A*) Experimental workflow for the analysis of lagging chromatin and anaphase bridges in cells following FdU treatment for 17 h (FdU) or folate deprivation for 3 d (No folate). (*B*) Representative images and (*C*) quantification of lagging chromatin and anaphase bridges containing the *FRAXA* locus in a panel of lymphocyte cell lines following FdU treatment. Unt, untreated. White arrows in *B* denote the location of *FRAXA*. (Scale bar in *B*: 5 μm.) (*D*) Quantification of lagging chromatin and anaphase bridges containing the *FRAXA* locus in a panel of lymphocyte cell lines following folate deprivation. Data are means of at least three independent experiments. Error bars represent SDs. ***P < 0.001; ****P < 0.0001.

We then investigated whether the *FRAXA* locus could generate chromatin bridges or UFBs in anaphase in response to either FdU or the absence of folate, as has been observed at CFSs in cells treated with APH (5). To this end, lymphocytes were treated with either FdU for 17 h or deprived of folate for 3 d (“No folate”), and then arrested in late G2 phase with the CDK1 inhibitor RO3306 (36), before being released into mitosis (Fig. 1*A*). Interestingly, we observed that the *FRAXA* locus was located on chromatin bridges and on lagging chromatin in all of the cell lines tested, although with markedly different frequencies (Fig. 1 *B*–*D*). In particular, aberrant mitotic segregation of *FRAXA* was seen strikingly in the FM GM09237 cell line containing more than 900 CGG repeats, reaching a level of 50% of the anaphases following FdU treatment and 30% of the anaphases following folate deprivation (Fig. 1 *B*–*D*). In addition, we confirmed that exposure to APH did not lead to missegregation of *FRAXA* in either the FM cells or a cell line containing normal *FRAXA* (GM20230), and that FdU did not induce CFS-associated anaphase bridges [using the widely studied CFS locus, *FRA16D*, as an example (37)] (*SI Appendix*, Fig. S2).

### *FRAXA* Localizes to Anaphase DNA Bridges Containing Single-Stranded DNA.

**Fig. 2.**
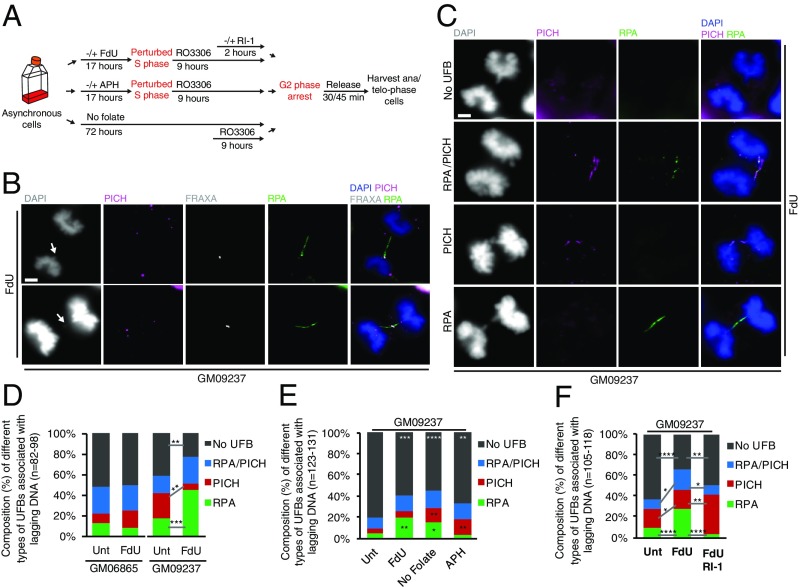
FM *FRAXA* is associated with RPA-coated UFBs in response to folate stress. (*A*) Experimental workflow for DNA bridge analysis in anaphase following treatment with FdU, APH, or deprivation of folate for 3 d. For cells treated with FdU, some were also treated with the RAD51 inhibitor, RI-1, during the final stages of G2. (*B*) Representative images of *FRAXA*+ve UFBs coated by RPA. *FRAXA* was detected using a specific FISH probe, and RPA was detected using an RPA antibody by IF. (*C*) Representative images and (*D*) quantification of PICH+ve, RPA+ve, or PICH/RPA+ve UFBs in GM06865 or GM09237 cells. (*E*) Quantification of PICH+ve, RPA+ve, or PICH/RPA+ve UFBs in GM09237 cells following the indicated treatments. (*F*) Quantification of PICH+ve, RPA+ve, or PICH/RPA+ve UFBs in GM09237 cells following FdU treatment in S phase and RI-1 treatment in late G2. In *B* and *C*, white arrows denote the *FRAXA*+ve lagging DNA. (Scale bar in *B* and *C*: 5 μm.) Data are means of at least three independent experiments. Error bars represent SDs. *P < 0.05; **P < 0.01; ***P < 0.001; ****P < 0.0001.

Because many of the *FRAXA-*containing chromatin bridges were apparently discontinuous and contained regions that were not stained by DAPI (Fig. 1*B*), we hypothesized that such DAPI-negative “gaps” might harbor UFBs. To test this hypothesis, we analyzed whether the PICH protein (6, 38), an established marker of UFBs, was present in these gaps (Fig. 2*A*). Surprisingly, PICH was rarely found in DAPI-negative regions of the DNA bridges containing *FRAXA* (Fig. 2*B*). Because PICH binds only to double-stranded DNA (dsDNA) (39), we examined whether RPA, the major single-stranded DNA (ssDNA) binding protein in human cells, might be present in the DAPI-negative gaps instead. Our results indicate that this was the case: Most of the gaps contained a UFB that was coated by RPA along almost all of its length (designated “RPA+ve” UFBs) (Fig. 2*B*). In addition, we observed that the staining patterns for PICH and RPA were generally mutually exclusive in those rare cases where UFBs were decorated by both of these proteins (designated “PICH/RPA+ve” UFBs) (Fig. 2*C*). Quantification of the frequency of PICH+ve, RPA+ve, or PICH/RPA+ve UFBs revealed that FdU treatment had a minimal effect on the spectrum of different UFB types in the cell line with normal *FRAXA* allele. In contrast, the cell line with FM *FRAXA* allele exhibited a significant increase of RPA+ve UFBs in response to FdU treatment (Fig. 2*D*). Furthermore, we confirmed that RPA+ve UFBs were also frequently observed in the No folate condition (Fig. 2 *A* and *E*). As a control, and consistent with previous findings, the majority of the UFBs induced by APH treatment were PICH+ve, and not RPA+ve (Fig. 2 *A* and *E*).

The discovery of *FRAXA*-associated, RPA+ve UFBs is intriguing because the presence of RPA-coated UFBs has only been observed in a small number of previous studies. These RPA-coated UFBs have been proposed to represent either underreplicated DNA or unresolved homologous recombination (HR) intermediates (40, 41). We therefore assessed whether the RPA-associated UFBs arising at *FRAXA* in response to FdU are dependent upon the major recombinase in human cells, RAD51. We observed that, following addition of the RAD51 inhibitor, RI-1, in G2 cells, there was a striking decrease in the frequency of RPA+ve UFBs and a concomitant increase in PICH+ve UFBs (Fig. 2 *A* and *F* and *SI Appendix*, Fig. S4). These data suggest that RPA+ve UFBs arise due to persistence of unprocessed HR intermediates, rather than underreplication per se.

The above finding also prompted us to ask whether the proteins involved in marking the location of CFSs following APH treatment are also found at *FRAXA* under folate stress conditions. We therefore analyzed whether FANCD2 colocalizes with either the *FRAXA* locus or the RPA+ve UFBs in response to FdU treatment (*SI Appendix*, Fig. S3). In this analysis, we focused on the FdU treatment only, since this treatment induces a higher rate of RPA+ve UFBs than does folate deprivation. We only rarely detected colocalization of FANCD2 with *FRAXA* following FdU treatment of cells with either a normal *FRAXA* allele (GM06865) or an FM allele (GM09237) (*SI Appendix*, Fig. S3 *A*–*C*). Moreover, although FANCD2 was occasionally detected at the ends of PICH-associated UFBs, the vast majority of the RPA+ve UFBs induced by FdU treatment were FANCD2-negative (*SI Appendix*, Fig. S3 *D*–*F*).

### *FRAXA* Is Located in Micronuclei and Is Missegregated During Folate Stress.

**Fig. 3.**
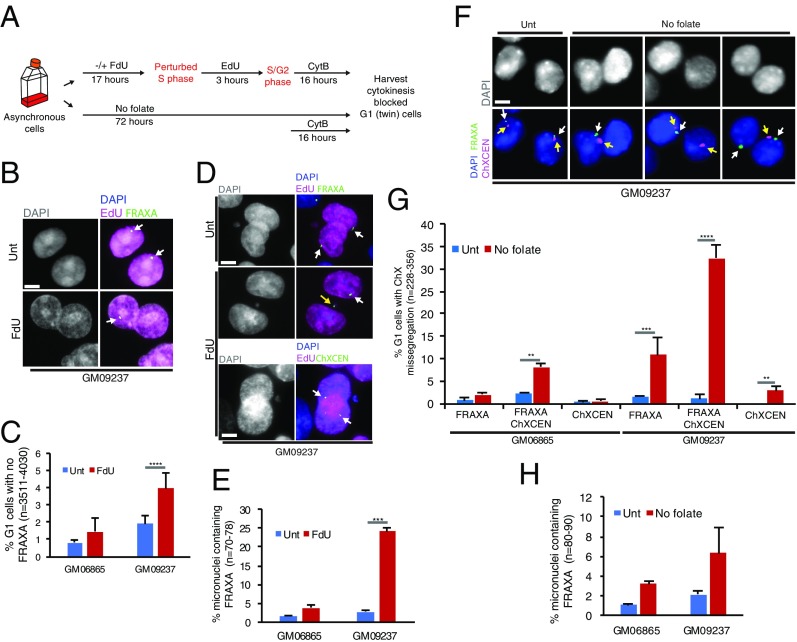
FM *FRAXA* is frequently located in a micronucleus, or lost in daughter G1 cells following folate stress. (*A*) Experimental workflow for quantifying *FRAXA* loss in cytokinesis-blocked G1 twin cells following FdU treatment for 17 h or folate deprivation for 3 d. (*B*) Representative images and (*C*) quantification of G1 cells that have lost *FRAXA.* White arrows denote *FRAXA* located in the nucleus. (*D, Top*) Representative images of the location of *FRAXA* in G1 cells in untreated cells. (*Middle*) Representative images of *FRAXA* located within a micronucleus in FdU treated cells allowed to progress into G1. (*Bottom*) Representative images of the location of *ChXCEN* in FdU treated cells. White arrows denote *FRAXA* in the nucleus, and yellow arrow denotes *FRAXA* located in a micronucleus. The location of *FRAXA* or *ChXCEN* was detected by the relevant FISH probe. (*E*) Quantification of micronuclei containing *FRAXA* in G1 cells following FdU treatment of GM06865 or GM09237 cells. (*F*) Representative images of GM09237 G1 cells that have lost either *FRAXA* (*Lower*, second from right), or *ChxCEN* (*Lower*, right), or both *FRAXA and ChxCEN* (*Lower*, second from left) following 3 d of folate deprivation. *Upper*, phase contrast images of nuclei stained with DAPI. (*G*) Quantification of the events observed in *F* in GM06865 or GM09237 cells. White arrows denote *FRAXA*, and yellow arrows denotes *ChxCEN*. *FRAXA* and *ChxCEN* were detected by a FISH probe. (*H*) Quantification of micronuclei containing *FRAXA* in G1 cells following 3 d folate deprivation in GM06865 or GM09237 cells. (Scale bar in *B*, *D*, and *F*: 5 μm.) Data are means of at least three independent experiments. Error bars represent SDs. **P < 0.01; ***P < 0.001; ****P < 0.0001.

To investigate whether the aberrantly segregated DNA containing *FRAXA* could disrupt the normal inheritance of the *FRAXA* locus in daughter cells, we analyzed cytokinesis-blocked “twin-daughter” G1 cells using FISH to define the location of *FRAXA*. To ensure that we analyzed only the cells that had undergone a perturbed round of DNA replication in the presence of FdU, we labeled cells that had traversed S phase by incubating them with 5-ethynyl-2′-deoxyuridine (EdU) for 3 h following FdU treatment (Fig. 3*A* and *SI Appendix*, Fig. S5). Hence, we only scored binucleated (twin-daughter) G1 cells that were EdU-positive. Consistent with the significantly high frequency of FRAXA-containing DNA bridges in anaphase in FM cells, we observed an increased frequency of *FRAXA* loss in one of the daughter nuclei in these cells, an effect that was not seen in the cells with a normal *FRAXA* allele (Fig. 3 *B* and *C*). In addition, 25% of the FM daughter cells contained a *FRAXA*-positive micronucleus, a greater than fivefold increase in comparison with that of normal cells (Fig. 3 *D* and *E*). To verify whether *ChXCEN* was missegregated together with *FRAXA* in FM cells following FdU treatment, we conducted the same experiment using a FISH probe specific for *ChXCEN.* However, none of the micronuclei scored contained *ChXCEN,* and none of the daughter nuclei analyzed had lost *ChXCEN* (Fig. 3*D*, *Bottom*). Taken together, our data indicate that, during only a single cell cycle, FdU treatment of FM cells causes extensive mitotic missegregation of *FRAXA,* but not of ChrX in its entirety.

To investigate whether folate deprivation might also cause the mislocalization and/or loss of *FRAXA* in the daughter cells, we cultured cells without folate for 3 d and then analyzed cytokinesis-blocked twin-daughter G1 cells as above (Fig. 3*A*). We observed a clear increase in the frequency of *FRAXA* loss in the cells containing either a normal or a mutant *FRAXA* allele (Fig. 3 *F* and *G*). More strikingly, we observed a strong increase in the combined loss of *FRAXA* and *ChXCEN*, or the loss of only the centromere, in cells with a mutant *FRAXA* allele (Fig. 3 *F* and *G*). Unlike FdU treatment, however, folate deprivation led to only a modest increase in the frequency of micronuclei containing *FRAXA* (Fig. 3 *E* and *H*). One likely explanation for this is that any *FRAXA*-containing micronuclei would be lost from the population during the extended growth period required to deprive cells of folate.

### *FRAXA* and ChrX Are Unstable During Extended Folate Deprivation.

**Fig. 4.**
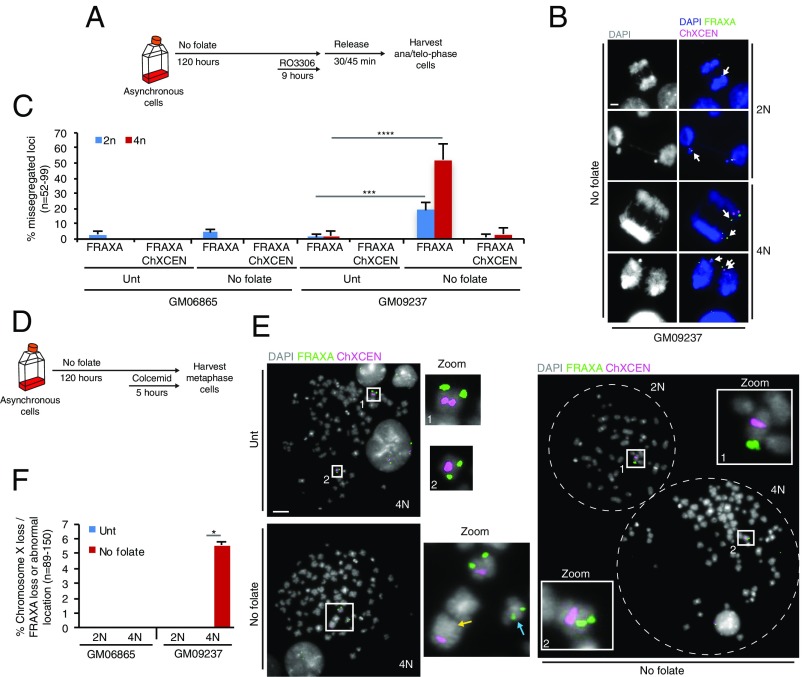
*FRAXA* and *ChXCEN* become unstable during extended folate deprivation. (*A*) Experimental workflow for analysis of anaphase cells following folate deprivation for 5 d. (*B*) Representative images and (*C*) quantification of missegregation of *FRAXA* and/or *ChXCEN* in either 2N or 4N cells with normal (GM06865) or FM (GM09237) *FRAXA* alleles. The white arrows in *B* denote the missegregated *FRAXA* or *ChXCEN.* (*D*) Experimental workflow for the analysis of mitotic chromosomes following folate deprivation for 5 d. (*E*) Representative images and (*F*) quantification of translocation or loss of *FRAXA* in metaphase spreads from the 4N population of cells with normal (GM06865) or FM (GM09237) *FRAXA* alleles. *E*, *Left* shows the location of *FRAXA* within chromosome X (*Upper*) or at an ectopic site (*Lower*). *E*, *Middle* shows zoomed images of the boxed area in *Left*. *E*, *Right* shows the loss of chromosome X in a 4N cell. The yellow and blue arrows in *E* denote the loss and abnormal location of *FRAXA,* respectively. (Scale bar in *B* and *E*: 5 μm.) Data are means of at least three independent experiments. Error bars represent SDs. *P < 0.05; ***P < 0.001; ****P < 0.0001.

In addition to missegregation of *FRAXA* in cells deprived of folate, we also observed an accumulation of binucleated progeny (4N cells) (*SI Appendix*, Fig. S6*A*). This was particularly evident when cells were cultured without folate for 5 d (*SI Appendix*, Fig. S6*B*). We reasoned that these binucleated cells would likely result from cytokinesis failure due to the presence of unresolved DNA bridges in telophase. This prompted us to address whether the abnormal segregation of *FRAXA* discussed above might be associated with the appearance of 4N cells. We therefore analyzed the segregation of ChrX in the cells deprived of folate for 5 d using FISH probes targeting either *FRAXA* or *ChXCEN.* We observed that there was a significantly greater increase in the frequency of abnormal segregation of *FRAXA* in the 4N progeny than in the 2N progeny in both normal and FM cells (Fig. 4 *A*–*C*). Interestingly, there was also a small, but measurable, increase of *ChXCEN* being missegregated in the 4N FM cells (Fig. 4 *A*–*C*). To investigate this in more detail, the location of *FRAXA* and *ChXCEN* was analyzed on metaphase chromosome spreads derived from either diploid (2N) or tetraploid) 4N cells deprived of folate (Fig. 4*D*). This showed that the *FRAXA* locus was either lost or located at an ectopic site in around 5% of the FM 4N cells but not in 2N cells or the 4N normal cells (Fig. 4*E*, *Left*, and *F*). Moreover, in some rare cases, the entire ChrX was lost in FM 4N cells (see example in Fig. 4*E*, *Right*). It is intriguing that the mitotic missegregation of *FRAXA* in the 4N cells was far more frequent than was the complete loss of *FRAXA*. We reasoned that this simply reflects the fact that a catastrophic chromosome event (e.g., the lagging DNA containing *FRAXA* or *ChxCEN*) can be captured while it is occurring in anaphase (Fig. 4*B*), but the majority of such events would lead to a change in ploidy or chromosome structure and hence cell death in the following cell cycles, which could not be scored in the metaphase spread analysis (Fig. 4 *E* and *F*).

### PM Allele Becomes Unstable Under Folate Stress.

Following the observation of dramatic changes at *FRAXA* in response to folate stress, we investigated whether folate stress might affect the stability of CGG repeats. To this end, we cultured cells in the absence or presence of folate stress (with FdU for 17 h, or no folate for 5 d), and then seeded cells as small populations (200 cells per well; designated “pooled clones”) in normal medium for 2 wk. Subsequently, we extracted DNA from each population of pooled clones and analyzed the distribution of *FMR1* CGG allele sizes (*SI Appendix*, Fig. S7*A*). We observed that the normal *FMR1* CGG allele remains stable under these conditions, with the PCR products from all of the pooled clones varying in size by no more than the equivalent of two CGG repeats (*SI Appendix*, Fig. S7*B* and Table S1). In contrast, there was significantly more variation in CGG allele length in the PM pooled clones, particularly in those cells that had been cultured under folate stress conditions, resulting in a decrease in the proportion of the population containing the initial allele length (*SI Appendix*, Fig. S7 *C* and *D* and Table S1 and Dataset S1). Moreover, there was a tendency for the PM allele to contract in size during folate stress (*SI Appendix*, Fig. S7 *C*, *E*, and *F* and Table S1 and Dataset S1).

## Discussion

To further understand the mechanism underlying the instability of folate-sensitive RFSs, we have analyzed the mitotic segregation of *FRAXA*, a locus containing an unstable CGG TNR. Our data demonstrate that folate stress causes a high level of mitotic instability in cells harboring a pathologically expanded CGG TNR region. Specifically, the FM *FRAX*A locus displays increased fragility in metaphase and aberrant segregation to daughter cells when the cells are treated with FdU or deprived of folate for a 3-d period, a pattern very reminiscent of that seen at CFSs challenged with APH (5). However, while most of the UFBs associated with CFSs following APH treatment comprise dsDNA and hence are coated with PICH, the folate stress-induced UFBs associated with *FRAXA* are largely RPA-associated ssDNA, suggesting that the source of the UFBs arising from these two types of fragile loci is fundamentally different. Consistent with this, the folate stress-induced RPA+ve UFBs are generally not associated with FANCD2, an established marker of CFSs (5, 7). Moreover, we could show that accumulation of RPA+ve UFBs depends on RAD51, which is essential for HR. These data suggest that *FRAXA*-associated UFBs represent predominantly unresolved HR intermediates, a new class of UFB discovered recently (40, 41). Indeed, our data provide evidence of an HR-dependent UFB derived from a specific locus in human cells. We propose that, during folate stress, replication of long CGG repeats is strongly perturbed, leading to replication fork collapse that drives HR at this locus in the late G2 phase.

Based on the striking level of *FRAXA* missegregation in FM cells, there seems little doubt that the cell struggles to replicate and segregate such long CGG repeat loci. In line with this notion, it is plausible that dietary folate deficiency could drive the FRAXA mosaicism observed in FXS patients (42, 43). Similarly, our data offer a potential explanation for the observation that ChrX aneuploidy occurs in female carriers and male FXS patients. The fact that we only observed *FRAXA* loss in tetraploid cells could simply reflect the fact that loss of ChrX (or even just the ChrX telomere region) (44) would probably be lethal to diploid male cells.

It is well established that PM alleles expand to FM from one generation to the next (42–44), but the mechanism underlying this expansion remains unknown. We have assessed whether folate stress could induce rapid expansion in only a few cell generations. Although our results did not indicate clear expansion of the PM allele, we did uncover evidence that the PM allele could contract in length following a short period of folate stress, which is consistent with the previous finding that CGG repeats can undergo either contraction or expansion (44). Further investigation is warranted to assess whether extended exposure to folate stress might lead to more dramatic changes in the PM alleles.

Taken together, the data presented here allow us to propose a model wherein the replication fork collapses at FM *FRAXA* CGG repeats under folate stress conditions, which initiates HR in late G2 phase (*SI Appendix*, Fig. S8). These HR intermediates persist into mitotic anaphase and form *FRAXA*-associated ssDNA UFBs or lagging chromatin. If these bridges and laggards fail to be resolved, which seems to occur in a significant proportion of the cases, *FRAXA* DNA can missegregate or form micronuclei in the next G1 phase. In some cases, *FRAXA*-associated UFBs in telophase might trigger the abortion of cytokinesis and lead to binucleation, presumably by activation of the abscission checkpoint (45, 46). This could give the cells another chance to replicate and divide, but inevitably would promote ChrX aneuploidy, and potentially more general chromosomal instability.

In this study, a specific locus has been tracked in cells deprived of folate and shown to be partitioned aberrantly between the newly born daughter cells. The methodologies developed here should facilitate future studies on replication stress and genomic instability. In addition, considering that numerous CG-rich repeat regions exist in the genome of all individuals, particularly those associated with CpG islands in gene promoters, further studies are warranted focusing on those regions. It is conceivable that folate deficiency could affect other, apparently nonpathological, CG-rich repeat regions in the human genome, which, over time, would drive progressive chromosome instability that has pathological consequences.

## Materials and Methods

The full details of cell lines, cell culture, cell synchronization, and treatment are described in *SI Appendix*, *Materials and Methods*. The procedures for Immunofluorescence (IF), FISH, FISH combined with IF, flow cytometry, Western blot analysis, and the FMR1 CGG allele assay are described in *SI Appendix*, *Materials and Methods*. Image and statistical analysis are also described in *SI Appendix*, *Materials and Methods*. In addition, the *FMR1* CGG allele PCR capillary electrophoresis output plots are included as Dataset S1.

## Supplementary Material

Supplementary File

Supplementary File
