# Supplementary material for: Folate deficiency drives mitotic missegregation of the human FRAXA locus
Source: Proc Natl Acad Sci U S A. 2018 Dec 3;115(51):13003–8. doi: 10.1073/pnas.1808377115 (PMC6304931; doi:10.1073/pnas.1808377115)
Supplement: Supplementary File [file pnas.1808377115.sapp.pdf]

Supplementary Information for

Folate deficiency drives mitotic missegregation of the human *FRAXA* locus

Victoria A. Bjerregaard, Lorenza Garribba, Cynthia T. McMurray, Ian D. Hickson, and Ying Liu

Corresponding author: Ying Liu
Email: ying@sund.ku.dk

This PDF file includes:

Supplementary text
Figs. S1 to S8
Table S1
References for SI reference citations

www.pnas.org/cgi/doi/10.1073/pnas.1808377115

SUPPLEMENTARY TEXT

MATERIALS AND METHODS

Cell lines and cell culture

Epstein-Barr virus immortalized human B-Lymphocyte cell lines containing different CGG repeat copy number at the *FRAXA* locus were from the Coriell Biorepository: GM06865 (male, with 27 CGG repeats), GM20230 (male, with 47 CGG repeats), GM06891 (male, with 100-117 CGG repeats), GM06852 (male, with over 200 CGG repeats) (1), and GM09237 (male, with 931-940 CGG repeats) (1, 2). The expression of the FMRP gene in the above cells lines was analyzed using conventional western blotting. The *FRAXA* CGG repeat length of cell lines expressing FMRP was confirmed by a published *FMR1* allele assay (3) by Laragen (Laragen Inc, USA). B-lymphocytes were maintained in RPMI 1640 Medium (Gibco) supplemented with 15% fetal bovine serum (FBS; ThermoFisher Scientific) and antibiotics. The cells were maintained at 37°C in a humidified atmosphere with 5% CO2, and were routinely subjected to mycoplasma testing (using MycoAlert; Lonza). Only mycoplasma-free cells were analyzed.

Cell synchronization and treatment

To obtain metaphase chromosomes, asynchronous cells under various conditions including untreated (Unt), with 0.5 μM 5-Fluorodeoxyuridine (FdU; Abcam), 0.4 μM aphidicolin (APH; Sigma Aldrich), or 0.4 mM hydroxyurea (HU; Sigma Aldrich) for 22h were synchronized in metaphase by being cultured with colcemid (100 ng/ml; ThermoFisher Scientific) for the last 5h of the 22h treatment. To analyze cells cultured with 'No folate' condition, cells were cultured in RPMI 1640 medium without folic acid (Gibco) for 72h, and were then synchronized at metaphase by being cultured with colcemid (100 ng/ml; ThermoFisher Scientific) for the last 5h before harvesting.

To harvest anaphase/telophase cells, asynchronous cells cultured with various conditions including untreated (Unt), with 0.5 μM 5-Fluorodeoxyuridine (FdU) (ab142670, Abcam), or 0.4 μM aphidicolin (APH) (89458-5MG, Sigma Aldrich) for 17h and synchronized in late G2 phase using RO3306 (12 μM) (217699, Merck) for 9h. Cells were then released

from the RO3306 arrest, incubated for 30 mins or 45 mins in pre-warmed (37°C) cell culture medium, and then seeded onto Poly L-Lysine coated slides (Sigma Aldrich) prior to fixation. For cells cultured in RPMI 1640 without folic acid (No folate), RO3306 was added to the medium after 72h of incubation, and the cells were released after 9 hours as described above. To inhibit RAD51 activity in G2 phase, RAD51 inhibitor RI-1 (60 μM) (ab144558, Abcam) was added during the last 2h of RO3306 synchronization. G2 arrested cells or anaphases/telophases were then harvested for further analysis.

To analyze micronucleus formation following FdU treatment, cells were released from FdU containing medium into medium containing 10 μM 5-ethynyl-2'-deoxyuridine (EdU; ThermoFisher Scientific) for 3h. Cells were then cultured with cytochalasin B (3 μg/ml; C6762, Sigma-Aldrich), in the absence of EdU, for 16h, and seeded onto Poly L-Lysine slides (Sigma-Aldrich) for further analysis.

Fluorescence in situ hybridization (FISH)

To analyze mitotic chromosomes, cells treated with colcemid as above were harvested using standard procedures. The cells were then swollen in 75 mM KCl (at 37°C), fixed in methanol:acetic acid (3:1) and then dropped onto glass slides. To analyze anaphase or G1 cells, cells were seeded on Poly L-Lysine slides (Sigma-Aldrich) and fixed in methanol:acetic acid (3:1). FISH probes where prepared from the following BAC clones: RP11-383P16 (GenBank: AC233288.1) for *FRAXA*, RP11-249C12 (GenBank: AQ508215.1) for *ChXCEN*, and RP11-264L1 (GenBank: AC046158.6) for *FRA16D*. Probes were labeled using the BioNick labeling system (Thermo Fisher Scientific) or DIG-nick translation mix (Sigma Aldrich). FISH was carried out using standard procedures. Briefly, samples were treated with RNase A, followed by dehydration in an ethanol series (70%, 90%, 96%) at RT for 2 mins each. Samples were denatured in 70% formamide at 75°C for 3 mins, and dehydrated again in an ice-cold ethanol series (70%, 90%, 96%) for 2 mins each. FISH-probe hybridization was carried out at 37°C for 16 to 72h. Biotin-conjugated probes were detected using avidin-FITC (ThermoFisher Scientific) and biotin-labeled anti-avidin (Vector Labs). DIG-conjugated FISH probes were detected using anti-digoxegenin-rhodamine (Roche) and Alexa Fluor 568 donkey

anti-sheep IgG (Thermo Fisher Scientific). Slides were mounted using Vectashield mounting medium with DAPI. Images were captured using an Olympus BX63 microscope and analyzed using CellSens (Olympus) or Fiji/ImageJ software.

Immunofluorescence (IF) analysis

To analyze G2 phase cells with IF, RO3306 arrested cells were seeded onto coated cytoslides (Thermo Fisher Scientific) at 500 rpm using a Cytospin 4 cytocentrifuge (Thermo Fisher Scientific) and then fixed in 4% paraformaldehyde (PFA) fixation buffer (4% PFA, 250 mM HEPES, 1xPBS, 0.2% Triton X-100) for 20 mins at room temperature (RT).

To analyze metaphase chromosomes with IF or FISH combined with IF, colcemid treated cells were swollen in hypotonic solution (10 mM Tris-HCl, pH 7.5, 10 mM NaCl, 5 mM MgCl2), harvested onto coated cytoslides at 850 rpm using Cytospin and then fixed in 4% PFA fixation buffer (4% PFA, 250 mM HEPES, 1xPBS, 0.4% Triton X-100) for 20 mins at 4°C.

To analyze anaphase cells, following the treatment, cells were seeded onto Poly L-Lysine slides (Sigma-Aldrich) and harvested at low speed (300 rpm) using Cytospin and fixed in 4% PFA fixation buffer for 20 mins at 4°C. Samples were then blocked for 2h in 5% bovine serum albumin (BSA) and incubated with primary antibodies overnight at 4°C, and then with secondary antibodies for 1h at RT. Slides were either further processed for with FISH combined with IF, or mounted using Vectashield with or without DAPI (Vector Laboratories). Images were captured using Olympus microscopes (BX63 or IX83) and then analyzed using CellSens (Olympus) or Fiji/ImageJ software. Primary antibodies used were: PICH (1:200, in-house), RPA (1:200, ab2175, Abcam), FANCD2 (1:400, NB100-182, Novus), cyclin A (1:100, ab16726, Abcam), RAD51 (1:100, sc8349, Santa Cruz Biotechnology). Secondary antibodies used were: For FANCD2: Alexa Fluor 647 donkey anti-rabbit IgG (1:500, A-31573; Thermo Fisher Scientific) or Alexa Fluor 568 goat anti-Rabbit IgG (1:500, A-110291; Thermo Fisher Scientific). For RAD51: Alexa Fluor 488 goat anti-rabbit (1:400, A-11008; Thermo Fisher Scientific). For PICH:

Alexa Fluor 488 goat anti-guinea pig IgG (1:500, A-11073; Thermo Fisher Scientific) or Alexa Fluor 568 goat anti-guinea pig IgG (1:500, A-11075; Thermo Fisher Scientific). For RPA and cyclin A: Alexa Fluor 568 goat anti-mouse (1:500, A-11004; Thermo Fisher Scientific) or Alexa Fluor 488 goat anti-mouse (1:500, A-150113; Thermo Fisher Scientific).

FISH combined with IF

First IF was carried out as described above. For antibodies that could tolerate the FISH conditions, samples were re-fixed in 8% PFA for 20 mins on ice prior to FISH analysis. For FISH, samples were denatured at 88°C for 5 mins and then processed as described above in FISH section. For antibodies that could not tolerate the FISH conditions, images were captured before and after FISH analysis using CellSens stage navigation. For these samples, the re-fixation step was carried out with 4% PFA fixation buffer and the DNA was denatured at 80°C for 5 mins. Images were captured using an Olympus BX63 microscope and analyzed using CellSens (Olympus) or Fiji/ImageJ software.

Flow cytometry

Cells were harvested and fixed in 70% ethanol (added dropwise while vortexing) and stored at -20°C for a minimum of 2h. Cells were centrifuged at 500 g for 5 mins at 4°C and then washed once in 1% BSA in PBS, centrifuged again, and stained with staining solution (2 mg/ml propidium iodide (Thermo Fisher Scientific), 10 mg/ml RNase A (Sigma-Aldrich) in 1 x PBS for 30 mins at 37°C. Fluorescence-activated cell sorting analysis was carried out on a FACSCalibur flow cytometer (BD Biosciences). For each condition, at least 20,000 events were recorded, and data analysis was performed using FlowJo software.

Western blot analysis

Cells were lysed using cell extraction buffer (FNN0011; Life technologies), supplemented with Protease inhibitor cocktail (PIC, Roche). Following protein quantification of the whole cell lysate, aliquots of samples were incubated for 10 mins at 70°C with NUPAGE SDS sample buffer (ThermoFisher Scientific), run on a SDS–PAGE

gel and transferred to a Hybond-PVDF membrane (Amersham Pharmacia). The membrane was blocked in PBST with 5% non-fat dry milk (Sigma Aldrich) for 1h at RT and then incubated overnight with a primary antibody re-suspended in PBST (with 5% non-fat dry milk) at 4°C. Following 3 x 15 min washes using PBST, the membrane was incubated for up to 2h at RT with a secondary antibody re-suspended in PBST (with 5% non-fat dry milk) at room temperature, followed by 4 x 15 min washes in PBST. Luminata Forte HRP substrate (Millipore) was used for signal detection, and images were captured on an Amersham Imager 600. Primary antibodies used were: FMRP (1:1000, ab17722; Abcam), and Actin (1:1000, A3853, AC-40, Sigma Aldrich). Secondary antibodies used were: anti-rabbit IgG (1:1000, A6667; Sigma Aldrich) and anti-mouse IgG (1:1000, A4416; Sigma Aldrich).

The *FMR1* CGG allele assay in sub-populations of GM06891 cells

To analyze the effect of folate stress on CGG repeat stability in *FRAXA* pre-mutation allele, GM06891 cells were cultured under the following conditions: untreated (Unt) or exposed to FdU (0.5 μM) for 17 h, or in RPMI 1640 medium without folic acid for 5 days (No folate). The cells were then counted and seeded into 12-wells plates as sub-populations (pooled-clones; 200 cells/well) (Fig. S7). These small populations of cells were cultured with standard medium for 2 weeks and were then harvested for DNA extraction. DNA was extracted using a QIAamp DNA Micro Kit (Qiagen) following the manufacturer's instructions. DNA samples were then subjected to analysis of *FMR1* allele length (3) by Laragen (Laragen Inc, USA).

Statistical analysis

At least three independent experiments were carried out to generate each data set. Statistical significance in each case was calculated using chi-squared test. In the *FMR1* CGG allele assay in GM06981 cells, 23 pooled-clones per condition were analyzed and the Student's t-test was performed to calculate statistical differences.

A

Cell line	Cell type	Sex	CGG repeat number at *FRAXA*	Mutation classification
GM06865	B-lymphocyte	Male	27	normal
GM20230	B-lymphocyte	Male	47	normal
GM06891	B-lymphocyte	Male	100-117	premutation
GM06852	B-lymphocyte	Male	>200	full mutation
GM09237	B-lymphocyte	Male	931-940	full mutation

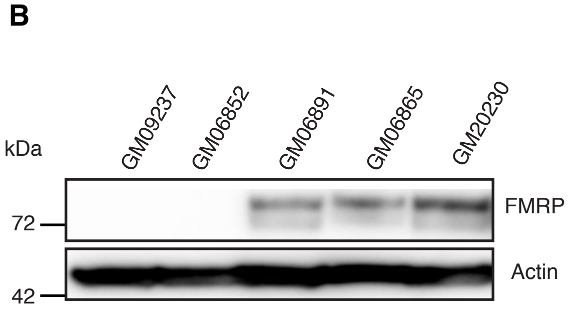

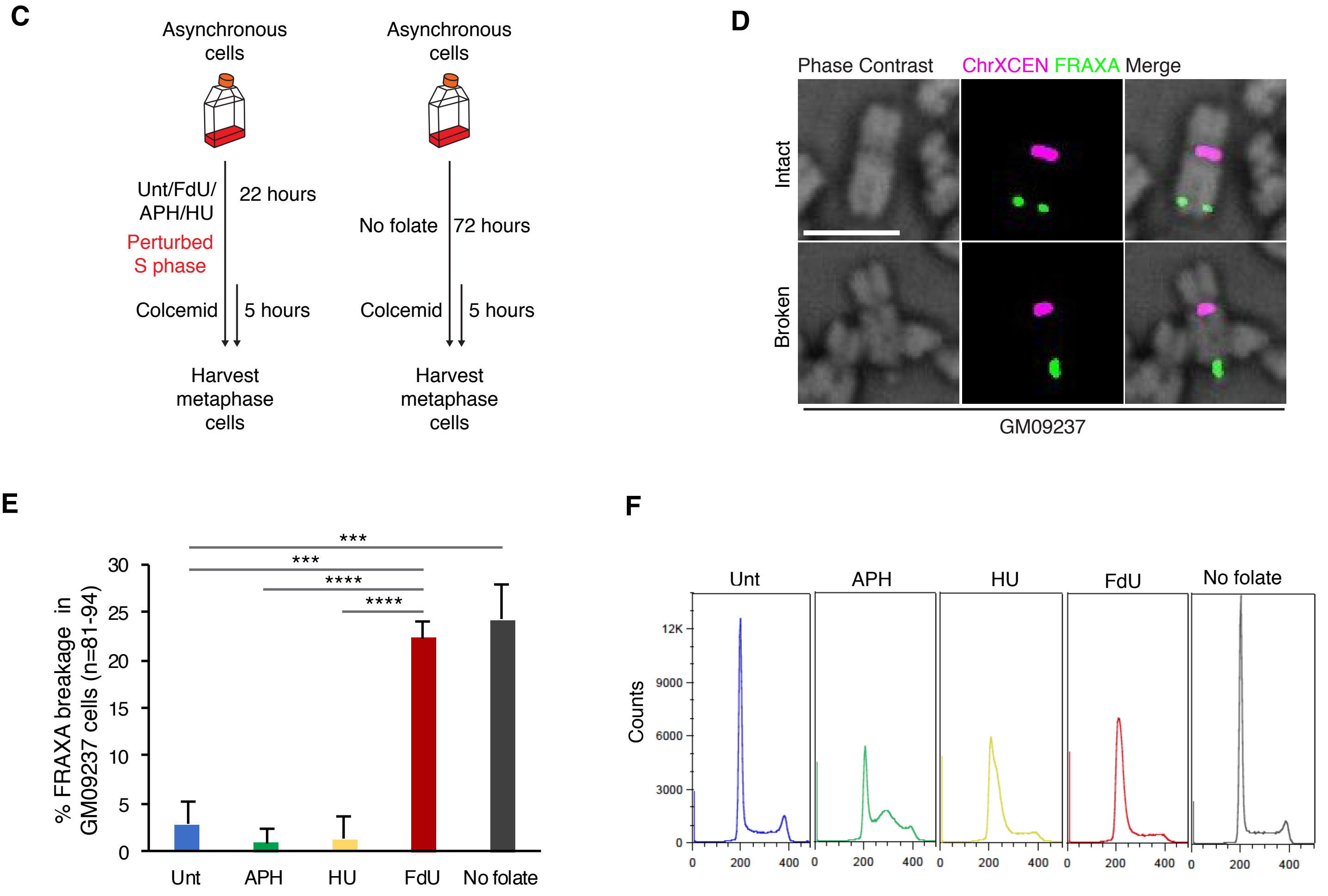

Fig. S1. (relates to Fig. 1). FdU treatment or folate deprivation, but not APH or HU treatment, induces fragility at FRAXA. (A) A table of the cell lines used in this study. B) Western blot analysis of FMRP protein expression in the panel of lymphocytes analyzed. (C) Experimental workflow for the analysis of fragility in metaphase chromosomes. Representative images (D) and quantification (E) of FRAXA fragility in GM09237 cells that have a FM FRAXA allele. A cell line with a normal FRAXA allele (GM06865) was analyzed in parallel. Fragility at FRAXA was not observed in GM06865 cells under any of the conditions tested (not shown). Scale bar, 5 µm. Data are means of at least three independent experiments. Error bars represent SDs (***denotes $p<0.001$; ****denotes $p<0.0001$;). F) Representative flow cytometry profiles of GM09237 cells under different conditions prior to the addition of colcemid for metaphase chromosome analysis.

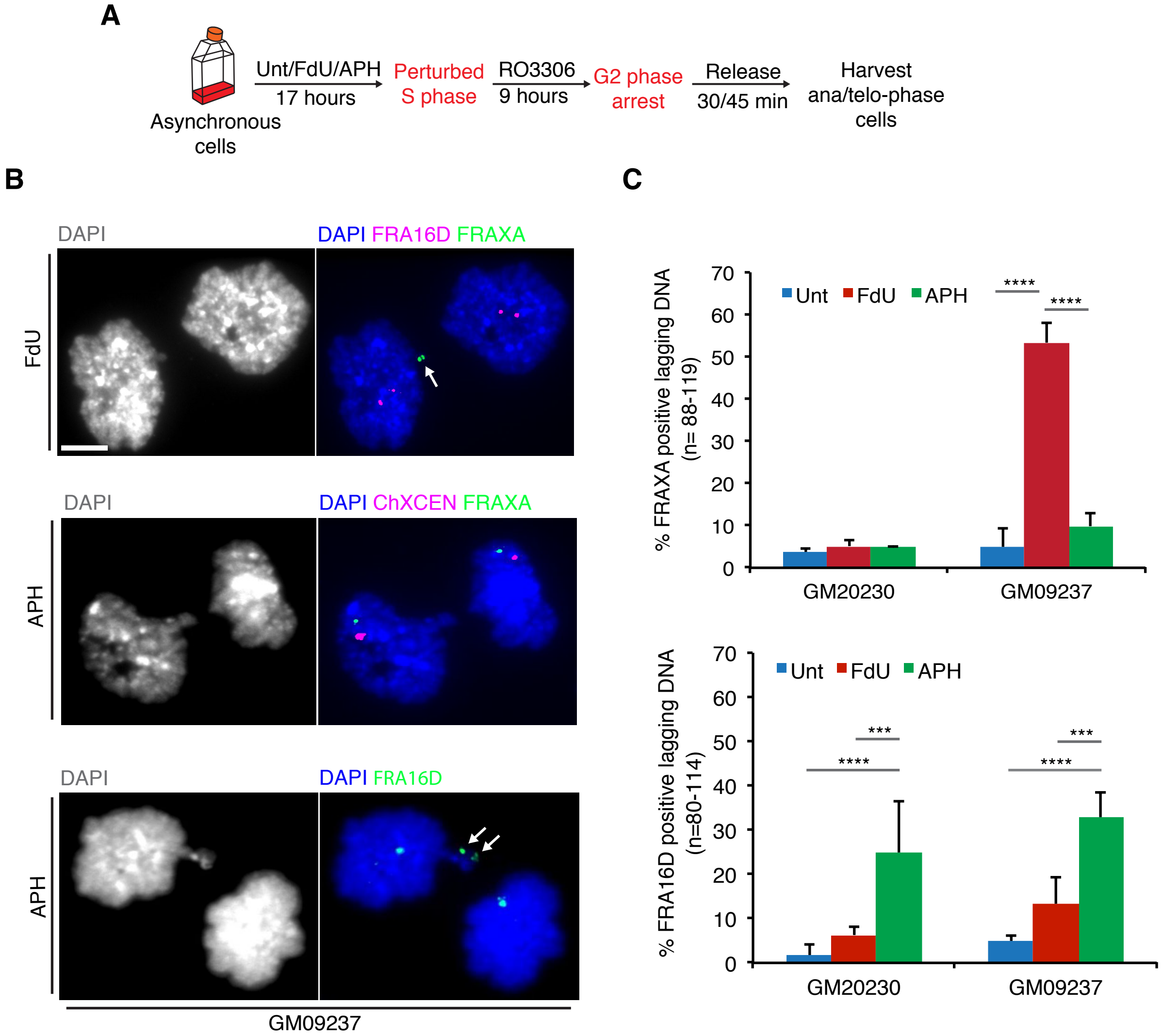

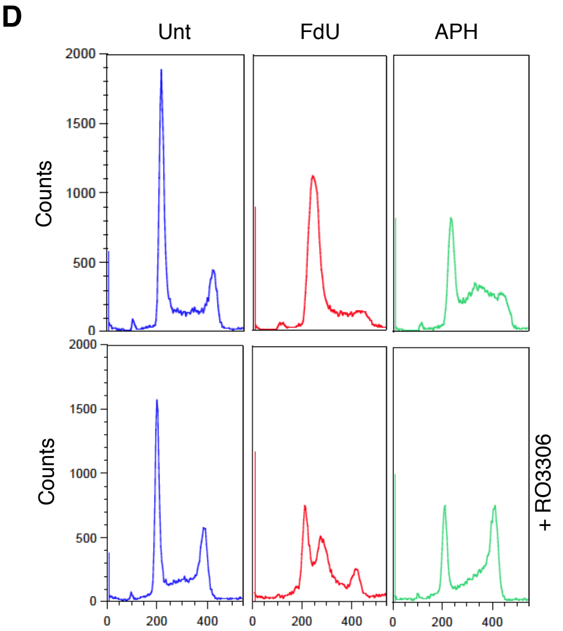

Fig. S2. (relates to Fig. 1). FRAXA associated lagging DNA is only induced by FdU treatment, while CFS associated lagging DNA at FRA16D is only induced by APH treatment. (A) Experimental workflow for the analysis of lagging DNA in anaphase cells. Representative images (B) and quantification (C) of lagging DNA containing FRAXA or FRA16D in cells with normal (GM20230) or FM (GM09237) FRAXA alleles under FdU or APH conditions. White arrows indicate lagging DNA containing FRAXA or FRA16D. The FRAXA and FRAX16D loci were detected using the corresponding FISH probes. A chromosome X centromere probe (ChXCEN) was used as a control for the normal segregation of the other parts of chromosome X under FdU or APH conditions. Scale bar, 5 µm. Data are means of at least three independent experiments. Error bars represent SDs. (***denotes $p<0.001$; ****denotes $p<0.0001$). (D) Representative flow cytometry profiles of GM09237 cells under different conditions prior to and after the addition of RO3306 for anaphase/telophase cell analysis.

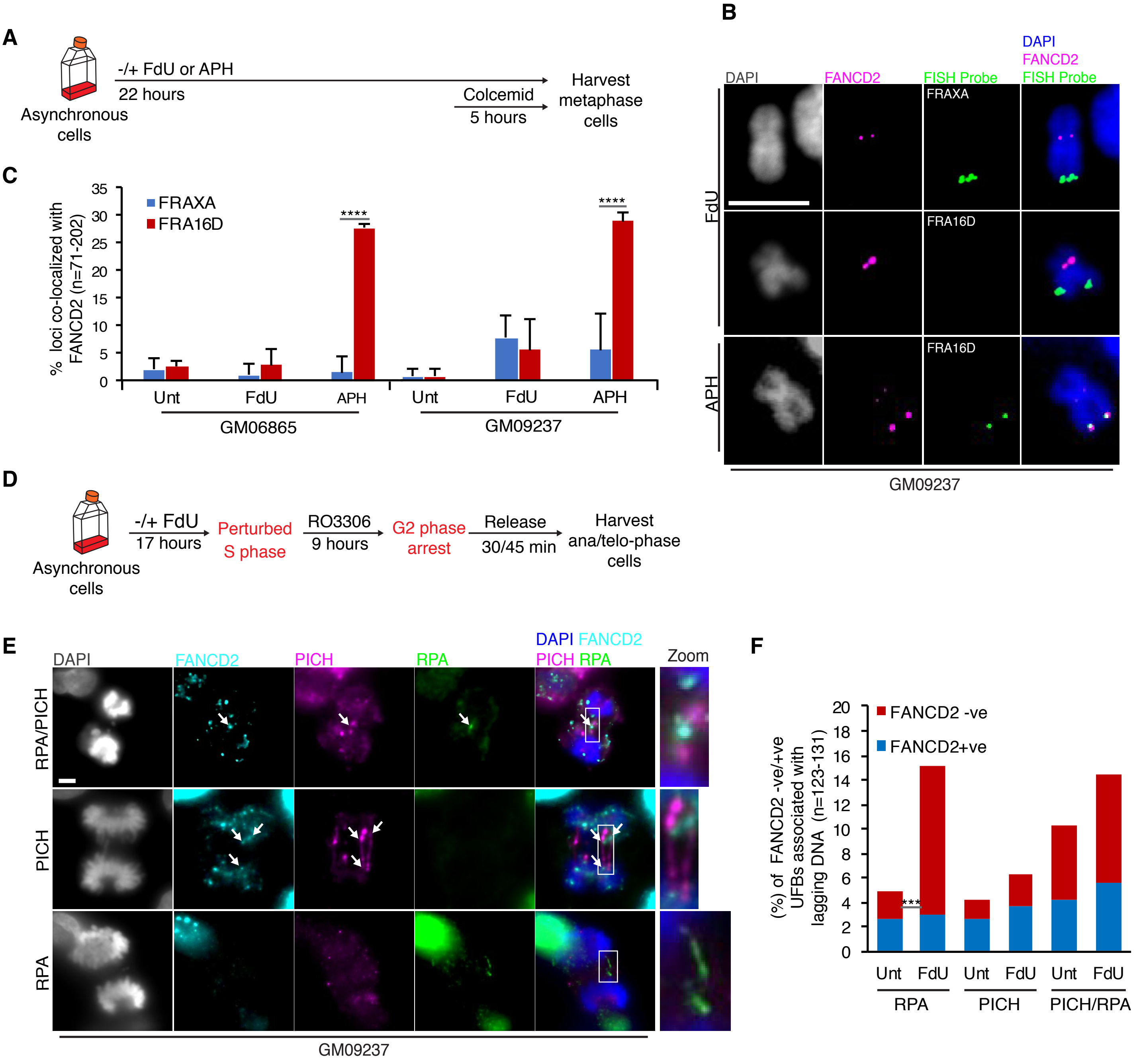

Fig. S3. (relates to Fig. 2). The FM FRAXA locus is not marked by FANCD2 following FdU treatment. (A) Experimental workflow for the analysis of metaphase chromosomes following FdU or APH treatment. (B) Representative images of the location of FANCD2 foci (red) on chromosomes in FM GM09237 cells treated with either FdU (top two panels) or APH (bottom panel). FRAXA or FRA16D was detected by the corresponding FISH probe respectively (green). (C) Quantification of the co-localization of FRAXA or FRA16D with FANCD2 foci following FdU or APH treatment in GM06865 or GM09237 cells. (D) Experimental workflow for the analysis of ana/telo-phase GM09237 cells by IF with a FANCD2 antibody combined with RPA, PICH, or RPA and PICH antibodies following FdU treatment. (E) Representative images of FANCD2 foci (blue) and anaphase UFBs that are bound with either RPA, or PICH, or RPA/PICH. White arrows indicate FANCD2 foci that localize adjacent to RPA or PICH. (F) Quantification of FANCD2 co-localization with either RPA, or PICH, or RPA/PICH UFBs in untreated or FdU treated conditions. Scale bar, 5 µm. Data are means of at least three independent experiments. Error bars represent SDs. (***denotes $p<0.001$; ****denotes $p<0.0001$).

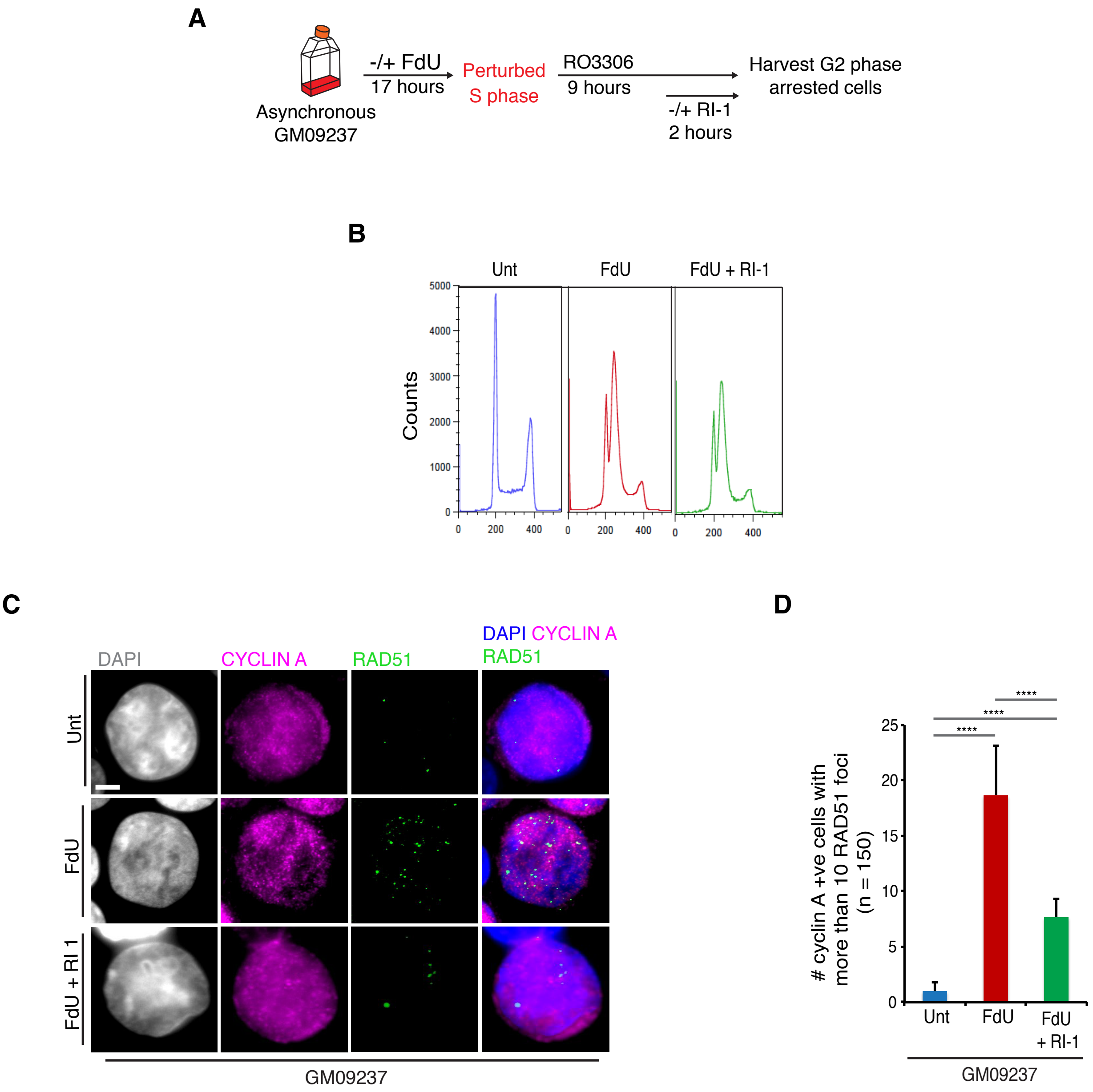

Fig. S4. (relates to Fig. 2). A RAD51 inhibitor (RI-1) reduces RAD51 activity following FdU treatment. (A) Experimental workflow for the analysis of the RAD51 inhibitor, RI-1, in G2 phase cells following FdU treatment. (B) Representative cell cycle profiles of GM09237 cells prior to being harvested for IF analysis following different treatments (untreated, FdU, or FdU combined with RI-1). (C) Representative images of RAD51 foci (green) in G2 phase cells (Cyclin A-positive; red) under the indicated conditions. (D) Quantification of Cyclin A-positive cells containing >10 RAD51 foci in G2 phase cells under the indicated conditions. Scale bar, 5 µm. Data are means of at least three independent experiments. Error bars represent SDs. (****denotes $p<0.0001$)

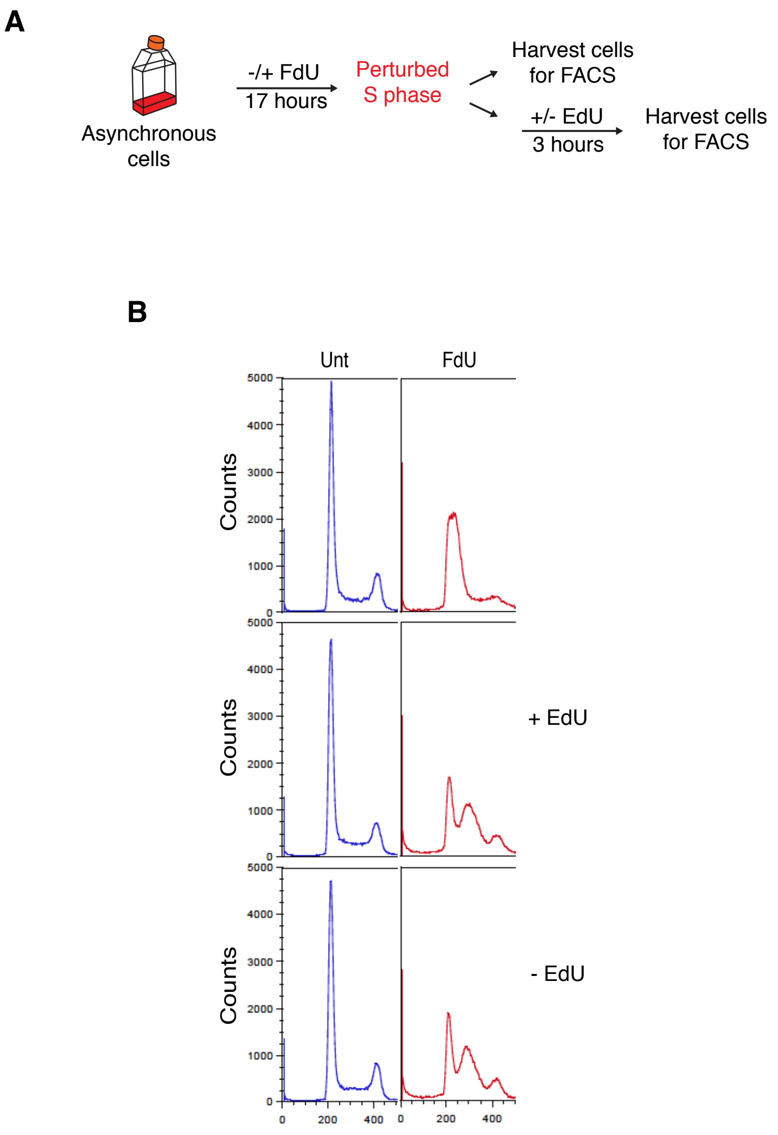

Fig. S5. (relates to Fig. 3) The cell cycle profile of cells treated with FdU and then labeled with EdU. (A) Experimental workflow for the treatment with FdU, and labeling with EdU. (B) Representative flow cytometry profiles of GM09237 cells harvested at the end of FdU treatment, or the end of EdU labeling.

A

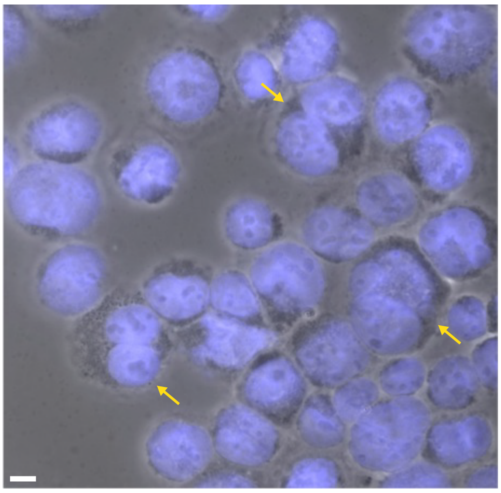

B

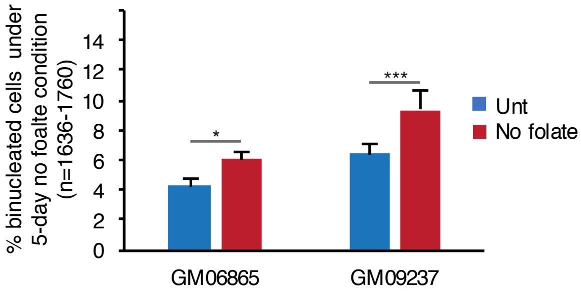

Fig. S6 (relates to Fig. 4). Extended folate deprivation causes an increase in the frequency of binucleated cells. (A) Representative images of cells with one or two nuclei. Yellow arrows indicate binucleated cells. Scale bar, 5 µm. B) Quantification of binucleation in normal and FM cells cultured in medium with or without folic acid for 5 days. (*denotes p<0.05, and ***denotes p<0.001)

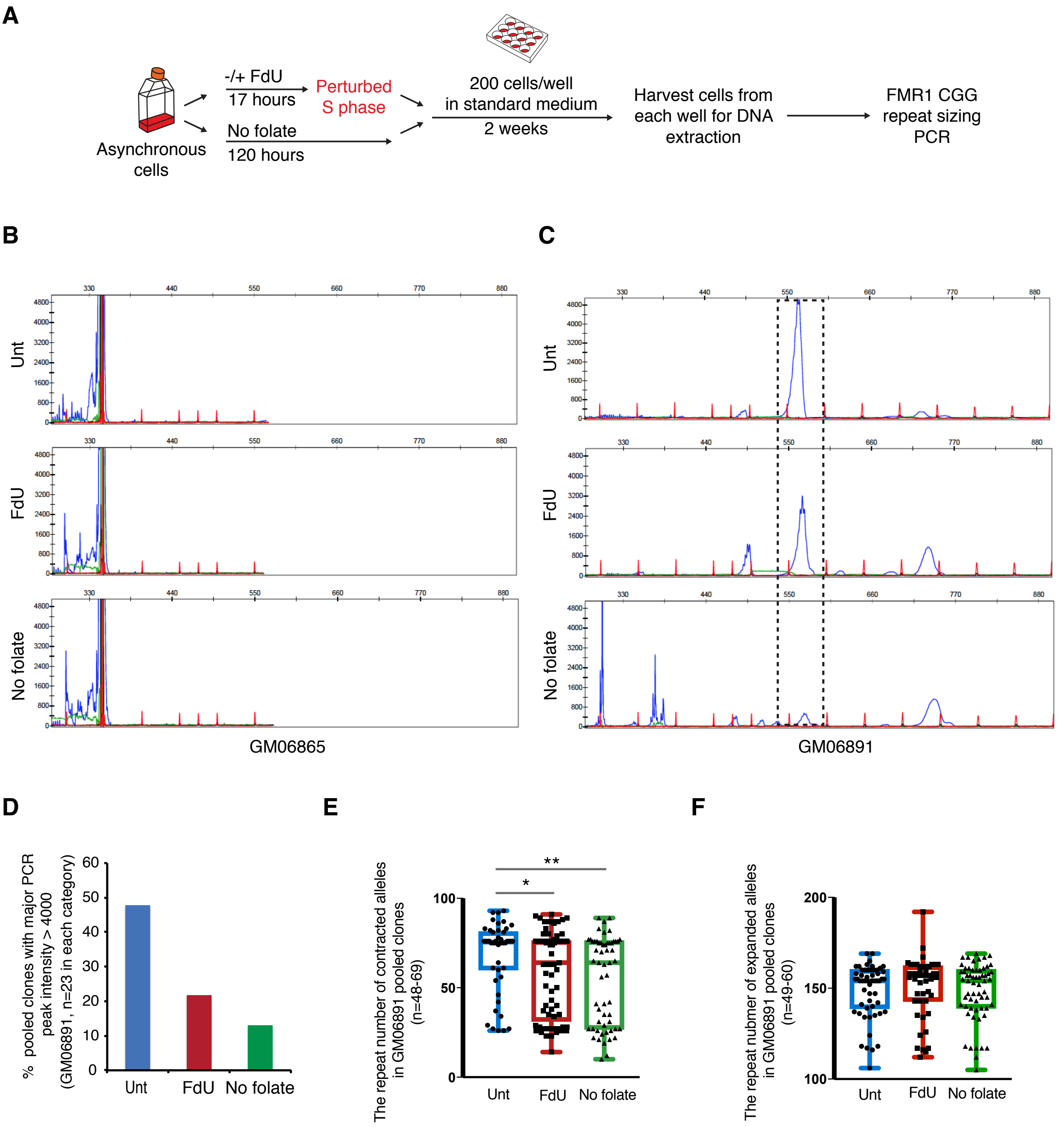

Fig. S7. The PM allele in GM06891 cells becomes unstable during folate stress. (A) Experimental workflow of the analysis of the FRAXA allele in PM cells (pooled clones) exposed to the indicated treatments. Representative images of the CGG repeat sizing PCR peaks in either normal (B) or PM (C) cell lines under the indicated treatment conditions. The x-axis indicates the PCR product size (bp) and the y-axis indicates the Relative Fluorescence Units (RFU) of the FAM-labeled PCR products. The major PCR peaks in PM cell line samples are highlighted in a dashed box. These peaks were quantified in (D). (D) Quantification of pooled clones with a major PCR peak above 4000 RFU after different treatment. Quantification and statistical analysis of the number of CGG repeats in contracted PM alleles (less than 98 repeats, set according to the minimum length of the expected repeat length in untreated samples; E) or expanded PM alleles (more than 103 repeats, set according to the maximum length of the expected repeat length in untreated samples; F) following the indicated treatments. (* denotes p<0.05, and **denotes p<0.01)

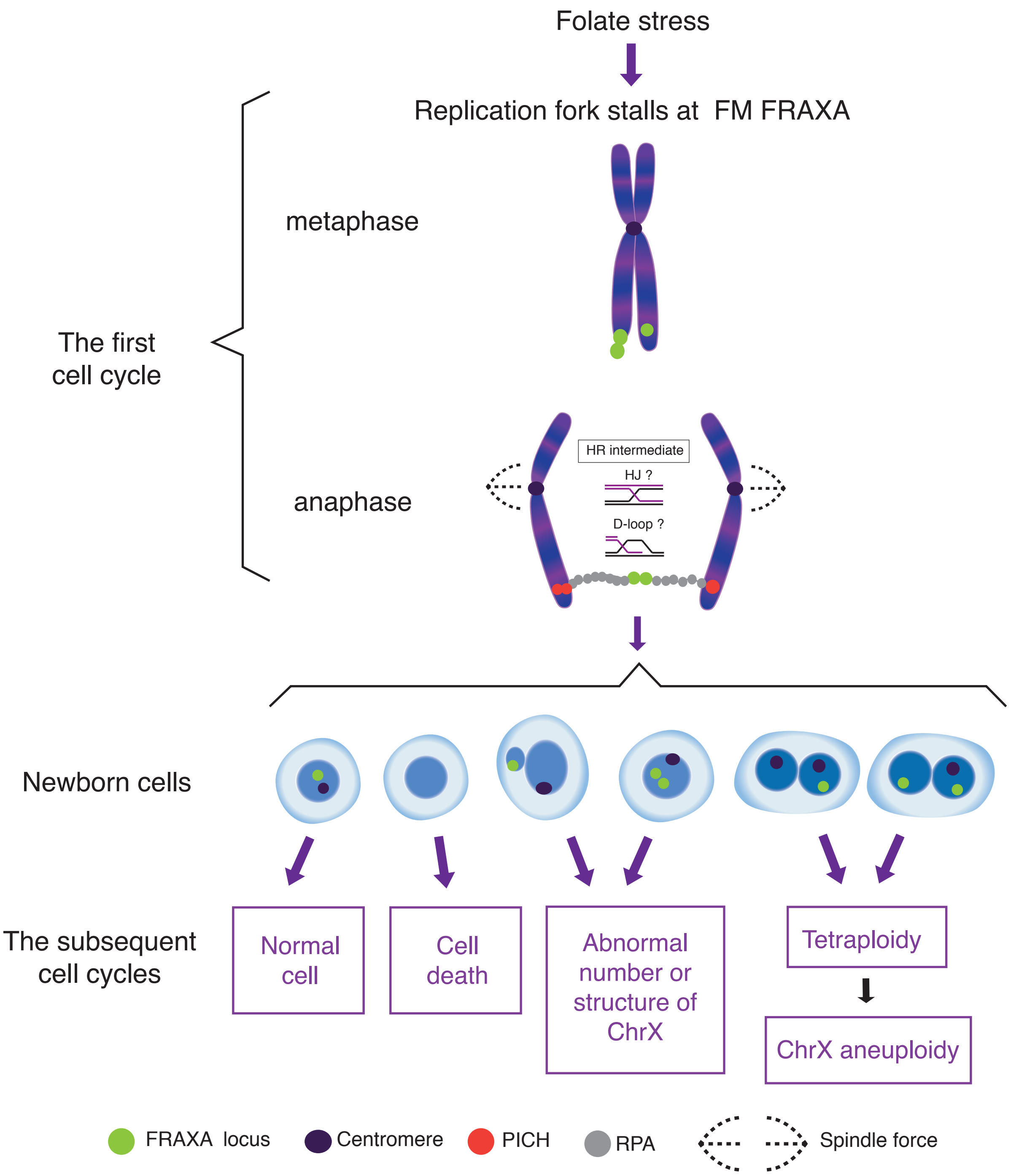

Fig. S8. The consequence of folate stress in cells with a mutant FRAXA allele.
In S phase, the replication of the FM allele is perturbed, leading to a greater chance of collapse of replication forks within FRAXA. This fork breakage initiates HR repair late in the cell cycle. A proportion of the HR intermediates (Holliday junctions, HJ; or D-loops) fail to be resolved prior to anaphase, leading to the formation of ssDNA bridges bound by RPA. This failure might be driven by the inability of the HR-derived DNA synthesis to progress through the CGG repeats. The FRAXA-containing bridges are ultimately either lost or retained in micronuclei in the next generation of G1 cells. Unresolved bridges might also trigger cytokinesis failure and hence promote the generation of binucleated cells, which tend to generate aneuploid progeny.

Table S1. A summary of the size of PCR products (bp) and the corresponding CGG repeat numbers for the alleles amplified from each clone-pools. PM: cell line GM06891 with a PM allele. N: cell line GM06865 with a normal allele. Unt: untreated. No folate: folate deprivation for 5 days. FdU: FdU treatment for 17 hours. R62: a control DNA sample.

Treatment	Sample Name	PCR Size1	PCR Size2	PCR Size3	PCR Size4	PCR Size5	PCR Size6	PCR Size7	PCR Size8	PCR Size9	CCG Size1	CCG Size2	CCG Size3	CCG Size4	CCG Size5	CCG Size6	CCG Size7	CCG Size8	CCG Size9
Unt	PM1	**564**	491	693	730	763					**98**	74	157	154	165				
Unt	PM2	**564**	544	503	677	724					**98**	92	78	136	152				
Unt	PM3	**564**	495	620	743						**98**	75	117	158					
Unt	PM4	**578**	495	748							**103**	75	160						
Unt	PM5	**564**	733								**98**	155							
Unt	PM6	**564**	732								**98**	154							
Unt	PM7	**564**	494	530	546	717	731	741			**98**	75	87	92	149	154	157		
Unt	PM8	**563**	493	731							**98**	75	154						
Unt	PM9	**570**	371	395	494	680	755				**100**	34	42	75	137	162			
Unt	PM10	**568**	495	709	732	748					**100**	92	147	149	154				
Unt	PM11	**568**	357	450	498	515	672	749			**100**	29	60	76	82	134	160		
Unt	PM12	**567**	452	497	517	640	689	733	749		**99**	61	76	83	124	140	155	160	
Unt	PM13	**570**	460	497	533	755					**100**	64	76	88	162				
Unt	PM14	**568**	349	497	587	623	675	735			**100**	27	76	106	118	135	155		
Unt	PM15	**567**	347	500	519	698	730	745			**99**	26	77	83	143	154	159		
Unt	PM16	**566**	431	496	695	735	757				**99**	54	76	142	155	163			
Unt	PM17	**452**	497	570	624	754	776				**61**	76	100	118	162	169			
Unt	PM18	**498**	571	737	756						**76**	101	156	162					
Unt	PM19	**568**	380	495	511	710	744				**100**	37	75	81	147	158			
Unt	PM20	**570**	408	497	618	681	732	750			**100**	46	76	116	137	154	160		
Unt	PM21	**568**	349	497	514	549	670	687	741		**100**	27	76	82	93	134	139	157	
Unt	PM22	**571**	438	495	527	732	753				**101**	56	75	86	154	161			
Unt	PM23	**567**	347	513	524	678	749	776			**99**	26	81	85	136	160	169		
No folate	PM24	**570**	373	383	478	515	535	677	743		**100**	35	38	70	82	89	136	158	
No folate	PM25	**568**	481	710	733	751					**100**	71	147	155	161				
No folate	PM26	**571**	348	495	682	705	732				**101**	26	75	138	145	154			
No folate	PM27	**569**	349	482	668	702	736	754			**100**	27	71	133	144	156	162		
No folate	PM28	**569**	350	498	705	740					**100**	27	76	145	157				
No folate	PM29	**569**	325	464	498	711	745	769			**100**	19	65	76	147	159	167		
No folate	PM30	**571**	346	493	747						**101**	26	75	159					
No folate	PM31	**568**	356	464	481	730					**100**	29	65	71	154				
No folate	PM32	**570**	299	345	461	687	724	776			**100**	10	25	64	139	152	169		
No folate	PM33	**568**	392	511	605	691	739	769			**100**	41	81	112	141	157	167		
No folate	PM34	**498**	304	349	436	518	572	749			**76**	12	27	56	83	101	160		
No folate	PM35	**569**	332	373	499	685	714	745	770		**100**	21	35	77	139	148	159	167	
No folate	PM36	**569**	459	499	674	750					**100**	63	77	135	160				
No folate	PM37	**571**	361	394	462	496	620	737	750		**101**	31	42	64	76	117	156	160	
No folate	PM38	**498**	361	529	568	699	752	774			**76**	31	87	100	143	161	168		
No folate	PM39	**568**	336	440	495	739					**100**	22	57	75	157				
No folate	PM40	**567**	391	492	709	752					**99**	41	74	147	161				
No folate	PM41	**568**	495	622	687	736					**100**	75	118	139	156				
No folate	PM42	**568**	349	493	583	690	758				**100**	27	75	105	140	163			
No folate	PM43	**571**	342	499	659	739	765				**101**	24	77	130	157	165			
No folate	PM44	**571**	331	496	619	672	729				**101**	21	76	117	134	153			
No folate	PM45	**571**	335	492	619	705	758				**101**	22	74	117	145	163			
No folate	PM46	**568**	535	619	691	733	767				**100**	89	117	141	155	166			
FdU	PM47	**568**	498	518	741						**100**	76	83	157					
FdU	PM48	**571**	312	418	458	492	531	741			**101**	14	50	63	74	87	157		
FdU	PM49	**571**	353	398	498	532	673	754	784		**101**	28	43	76	88	135	162	172	
FdU	PM50	**571**	350	496	741	770					**101**	27	76	157	167				
FdU	PM51	**569**	351	444	496	714	736	756			**100**	27	58	76	148	156	162		
FdU	PM52	**571**	414	526	698	750					**101**	48	86	143	160				
FdU	PM53	**569**	349	370	496	619	740				**100**	27	34	76	117	157			
FdU	PM54	**498**	375	388	459	531	569	670	699	740	**76**	35	40	63	87	100	134	143	157
FdU	PM55	**568**	350	496	642	678	710	742			**100**	27	76	124	136	147	158		
FdU	PM56	**568**	345	373	537	755					**100**	25	35	89	162				
FdU	PM57	**568**	346	498	757						**100**	26	76	163					
FdU	PM58	**568**	343	462	498	540	757				**100**	25	64	76	90	163			
FdU	PM59	**569**	354	495	535	613	743				**100**	28	75	89	115	158			
FdU	PM60	**568**	355	506	619	734					**100**	29	79	117	155				
FdU	PM61	**567**	338	441	492	530	697	728	757	845	**99**	23	57	74	87	143	153	163	192
FdU	PM62	**568**	422	495	509	647	743	757			**100**	51	75	80	126	158	163		
FdU	PM63	**568**	450	495	708	746					**100**	60	75	146	159				
FdU	PM64	**567**	458	497	530	731					**99**	63	76	87	154				
FdU	PM65	**498**	346	381	577	743					**76**	26	37	103	158				
FdU	PM66	**568**	339	499	759						**100**	23	77	163					
FdU	PM67	**571**	348	389	496	605	734	759			**101**	26	40	76	112	155	163		
FdU	PM68	**569**	350	382	509	542	573	743	756		**100**	27	38	80	91	101	158	162	
FdU	PM69	**568**	353	476	509	616	709	737	761		**100**	28	69	80	116	147	156	164	
Unt	N2	**345**									**25**								
Unt	N4	**345**									**25**								
Unt	N5	**345**									**25**								
Unt	N6	**345**									**25**								
Unt	N10	**346**									**26**								
Unt	N13	**345**									**25**								
Unt	N14	**345**									**25**								
Unt	N16	**345**									**25**								
Unt	N17	**345**									**25**								
Unt	N18	**345**									**25**								
Unt	N19	**347**									**26**								
Unt	N20	**345**									**25**								
Unt	N23	**345**									**25**								
No folate	N24	**349**									**27**								
No folate	N25	**345**									**25**								
No folate	N28	**347**									**26**								
No folate	N29	**345**									**25**								
No folate	N31	**345**									**25**								
No folate	N32	**344**									**25**								
No folate	N33	**345**									**25**								
No folate	N34	**346**									**26**								
No folate	N41	**345**									**25**								
No folate	N43	**345**									**25**								
No folate	N44	**349**									**27**								
No folate	N46	**348**									**26**								
FdU	N48	**344**									**25**								
FdU	N49	**345**									**25**								
FdU	N51	**345**									**25**								
FdU	N52	**345**									**25**								
FdU	N54	**345**									**25**								
FdU	N57	**345**									**25**								
FdU	N60	**345**									**25**								
FdU	N67	**345**									**25**								
FdU	N68	**344**									**25**								
n/a	R62*	**344**									**25**								

Supplementary References

1. Vincent A, et al. (1991) Abnormal pattern detected in fragile-X patients by pulsed-field gel electrophoresis. Nature 349(6310):624-626.
2. Seneca S, et al. (2012) Reliable and sensitive detection of fragile X (expanded) alleles in clinical prenatal DNA samples with a fast turnaround time. J Mol Diagn 14(6):560-568.
3. Hayward BE, Zhou Y, Kumari D, & Usdin K (2016) A Set of Assays for the Comprehensive Analysis of FMR1 Alleles in the Fragile X-Related Disorders. J Mol Diagn 18(5):762-774.
