# Supplementary material for: Folate deficiency drives mitotic missegregation of the human FRAXA locus
Source: Proc Natl Acad Sci U S A. 2018 Dec 3;115(51):13003–8. doi: 10.1073/pnas.1808377115 (PMC6304931; doi:10.1073/pnas.1808377115)

PNAS
www.pnas.org

Folate deficiency drives mitotic missegregation of the human *FRAXA* locus

Victoria A. Bjerregaard, Lorenza Garribba, Cynthia T. McMurray, Ian D. Hickson, and Ying Liu

Corresponding author: Ying Liu
Email: ying@sund.ku.dk

This PDF file includes:

Dataset S1: The output of the FMR1 CGG sizing PCR capillary electrophoresis in all of the samples analyzed (26 pages in total). The results and nature of the samples are summarized in Table S1.

www.pnas.org/cgi/doi/10.1073/pnas.180377115

20180922CGG

Sample File	Sample Name	Panel	OS	SQ
6-65-PM1_A09.fsa	6-65-PM1	R62_150-1500		

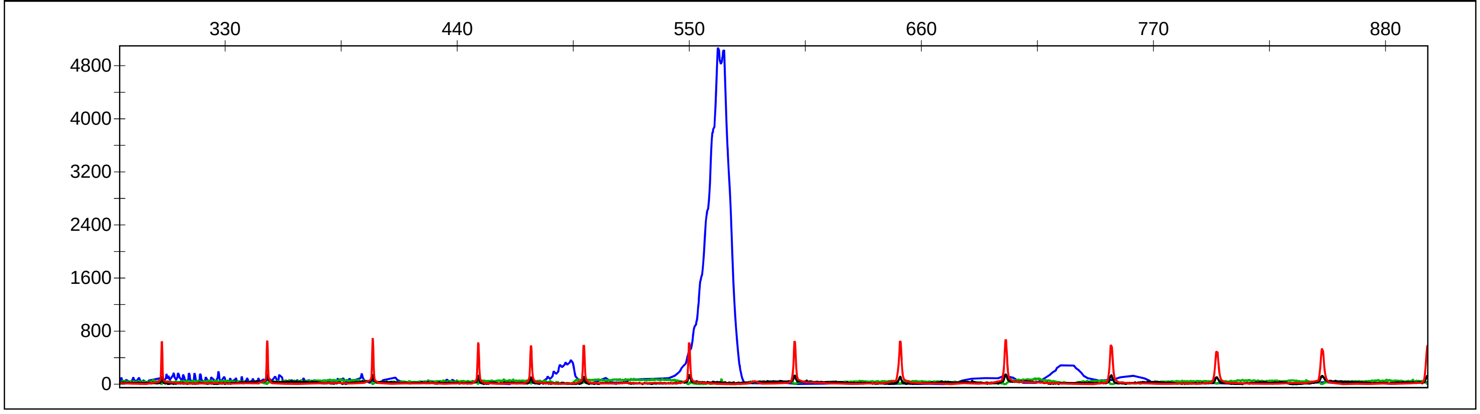

Sample File	Sample Name	Panel	OS	SQ
6-66-PM2_B09.fsa	6-66-PM2	R62_150-1500		

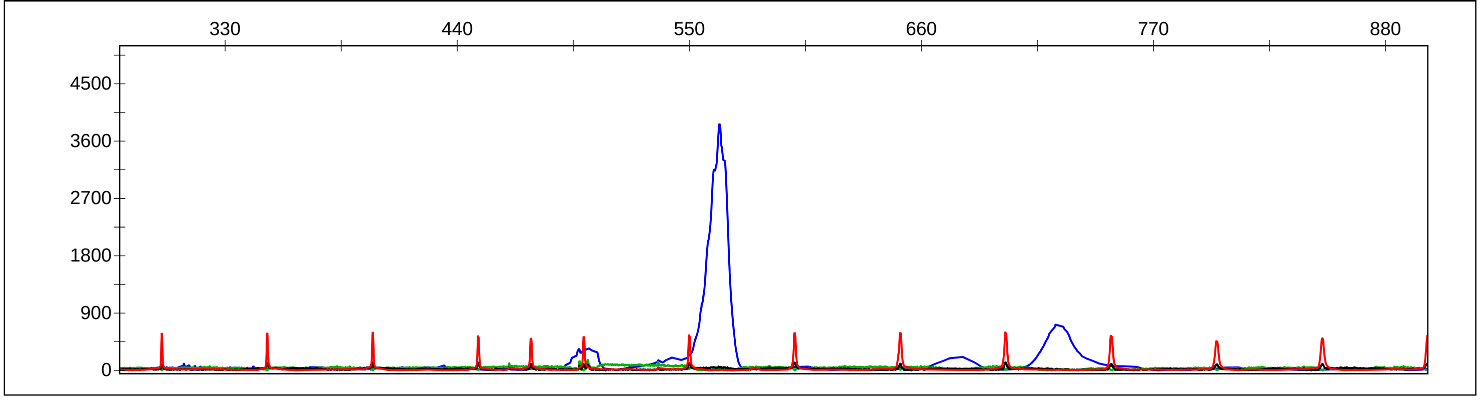

Sample File	Sample Name	Panel	OS	SQ
6-67-PM3_C09.fsa	6-67-PM3	R62_150-1500		

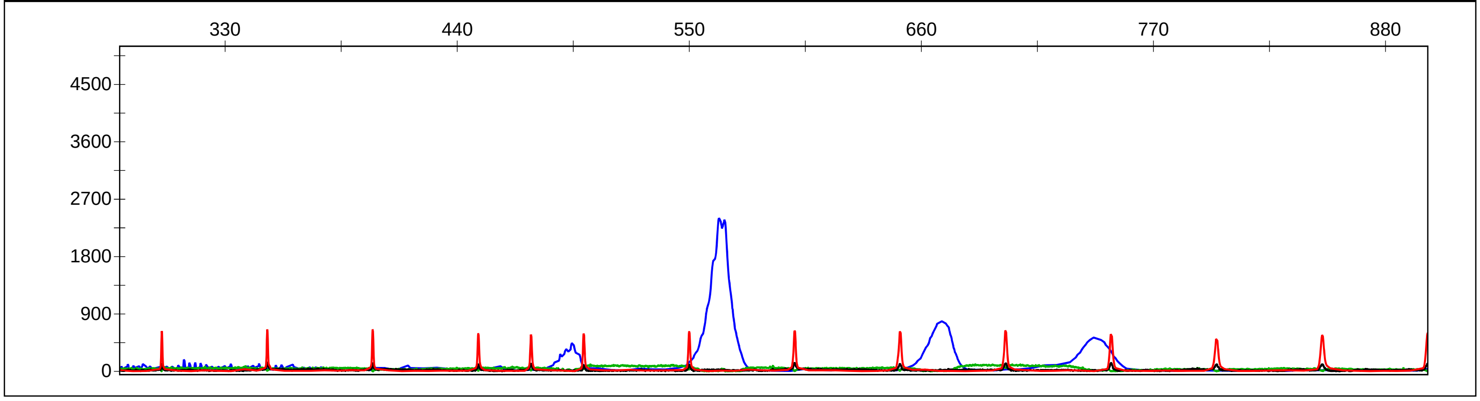

Sample File	Sample Name	Panel	OS	SQ
6-68-PM4_D09.fsa	6-68-PM4	R62_150-1500		

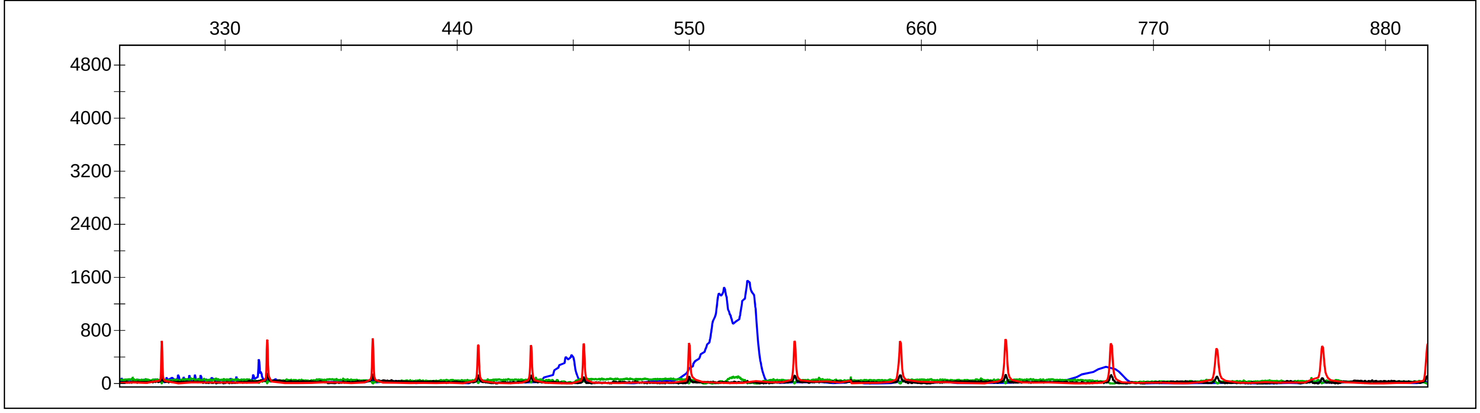

Sample File	Sample Name	Panel	OS	SQ
6-69-PM5_E09.fsa	6-69-PM5	R62_150-1500		

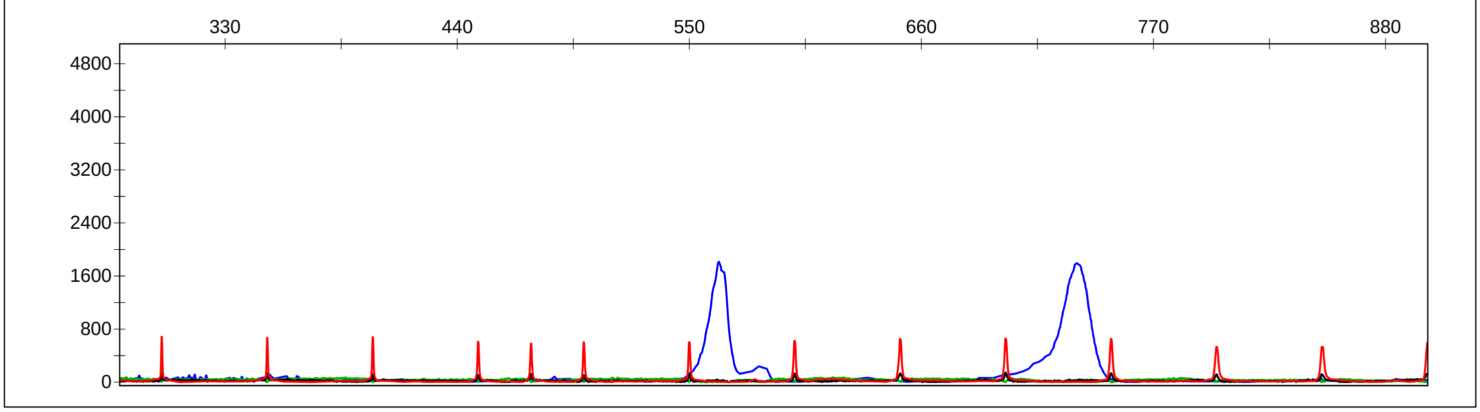

Sample File	Sample Name	Panel	OS	SQ
6-70-PM6_F09.fsa	6-70-PM6	R62_150-1500		

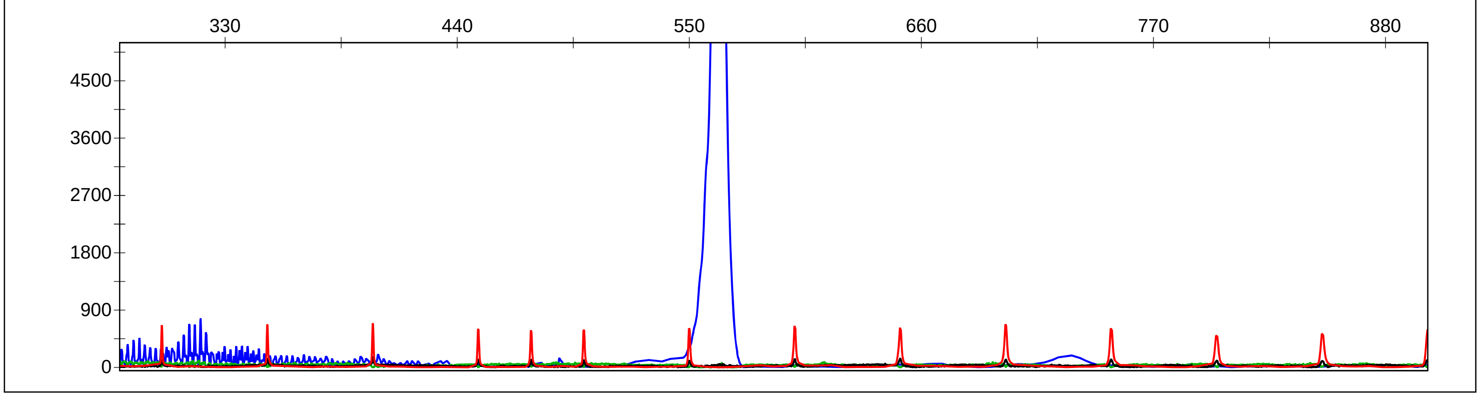

Sample File	Sample Name	Panel	OS	SQ
6-71-PM7_G09.fsa	6-71-PM7	R62_150-1500		

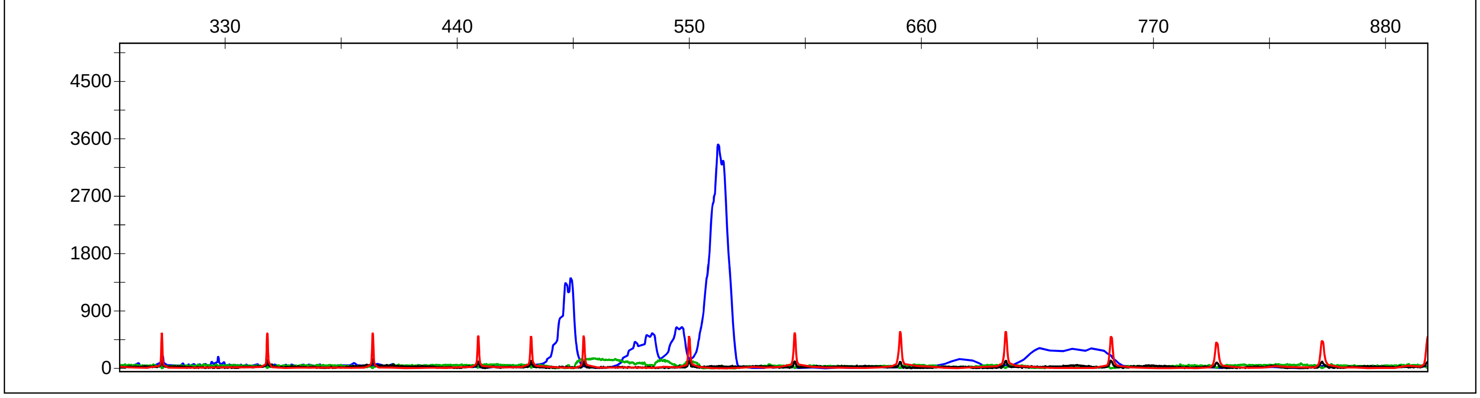

Sample File	Sample Name	Panel	OS	SQ
6-72-PM8_H09.fsa	6-72-PM8	R62_150-1500		

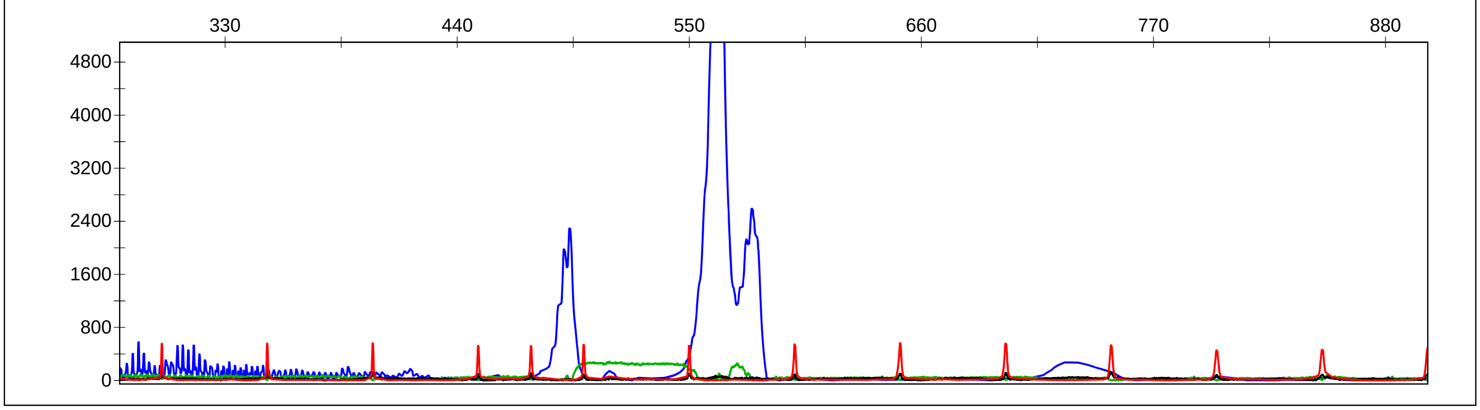

Sample File	Sample Name	Panel	OS	SQ
7-01-PM9_A01.fsa	7-01-PM9	R62_150-1500		

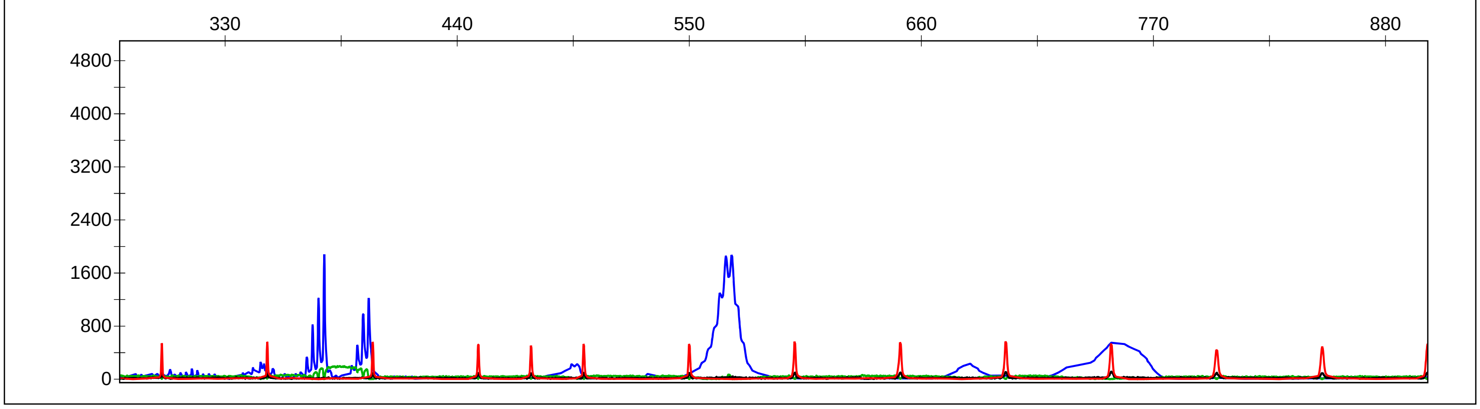

Sample File	Sample Name	Panel	OS	SQ
7-02-PM10_B01.fsa	7-02-PM10	R62_150-1500		

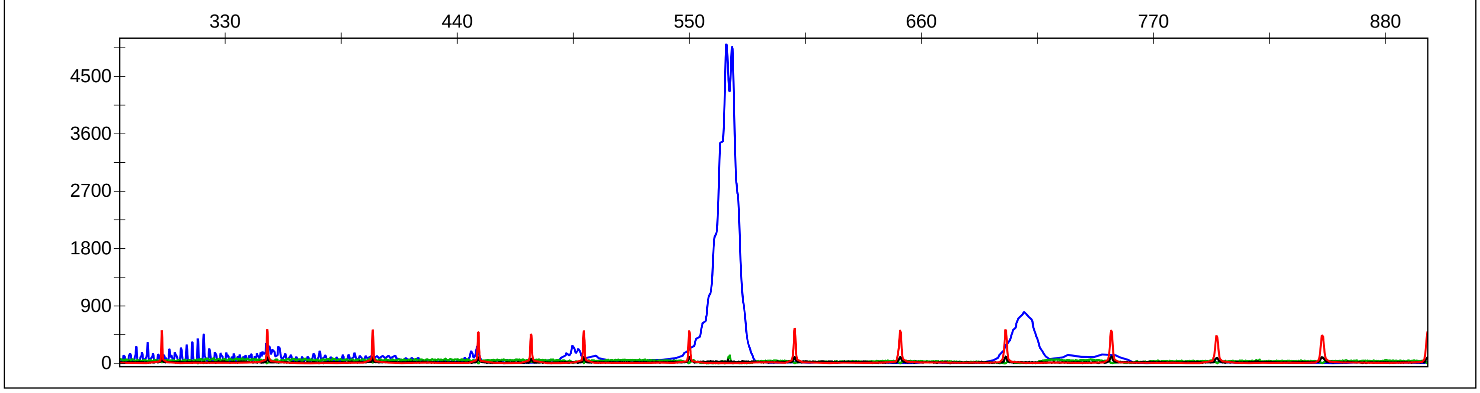

Sample File	Sample Name	Panel	OS	SQ
7-03-PM11_C01.fsa	7-03-PM11	R62_150-1500		

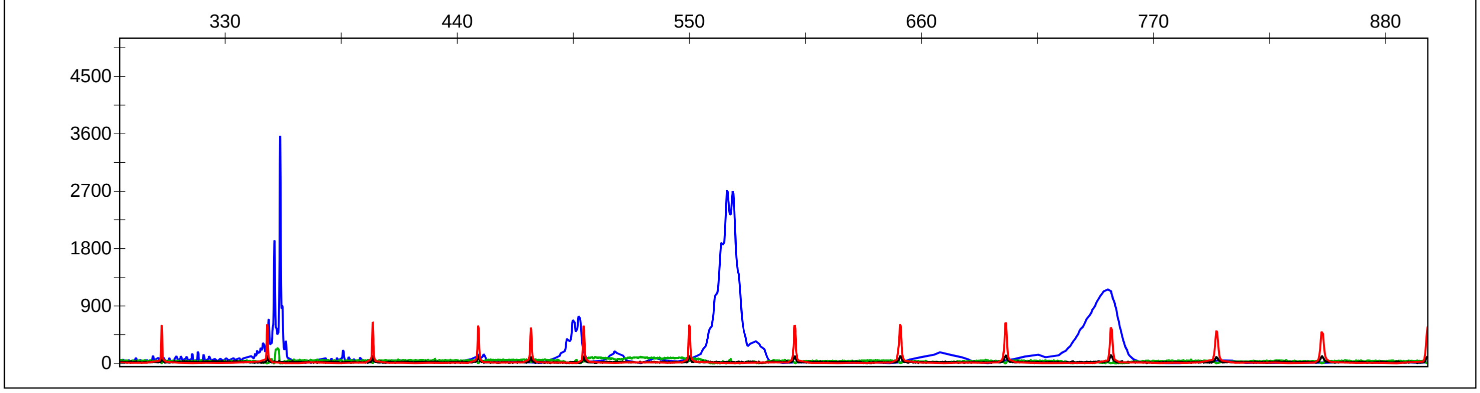

Sample File	Sample Name	Panel	OS	SQ
7-04-PM12_D01.fsa	7-04-PM12	R62_150-1500		

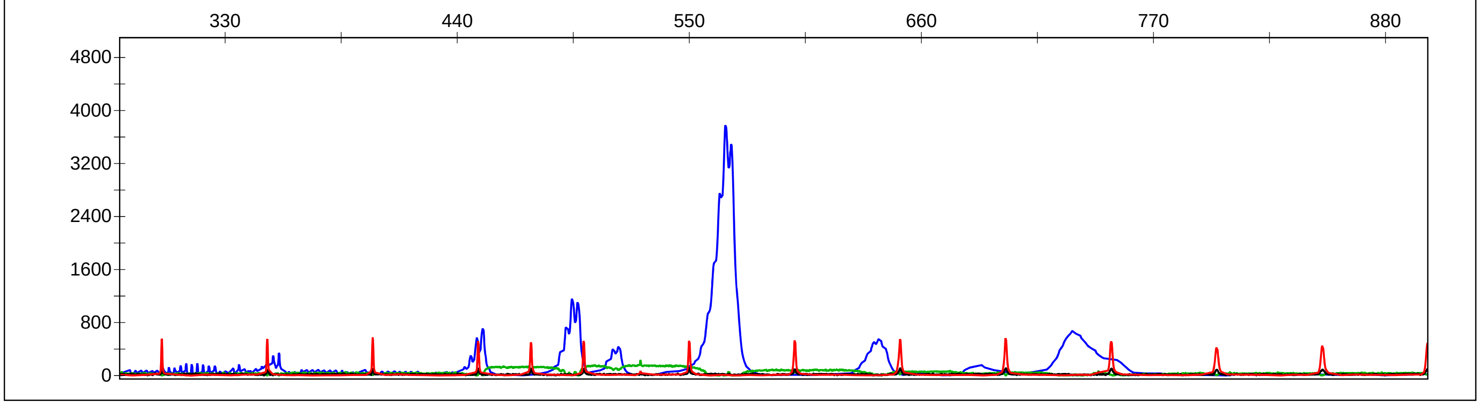

Sample File	Sample Name	Panel	OS	SQ
7-05-PM13_E01.fsa	7-05-PM13	R62_150-1500		

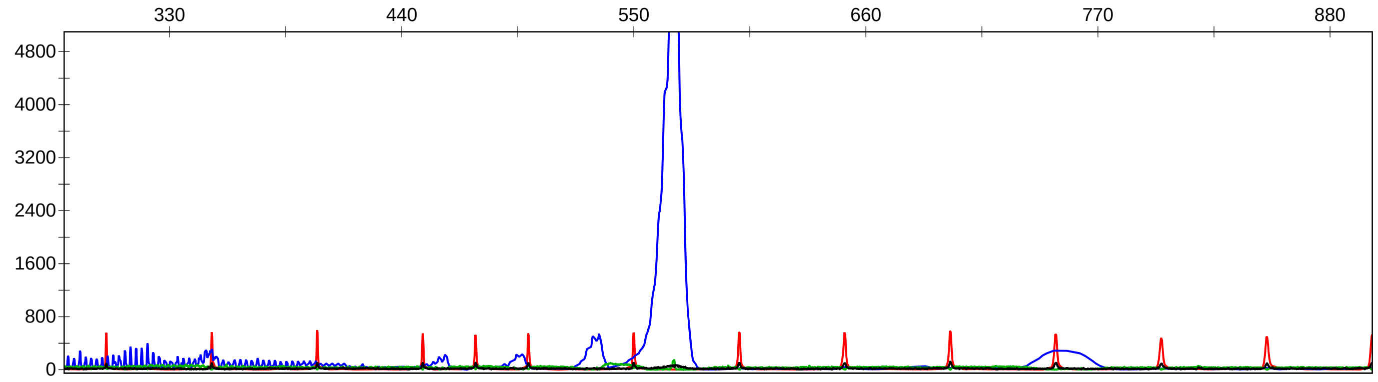

Sample File	Sample Name	Panel	OS	SQ
7-06-PM14_F01.fsa	7-06-PM14	R62_150-1500		

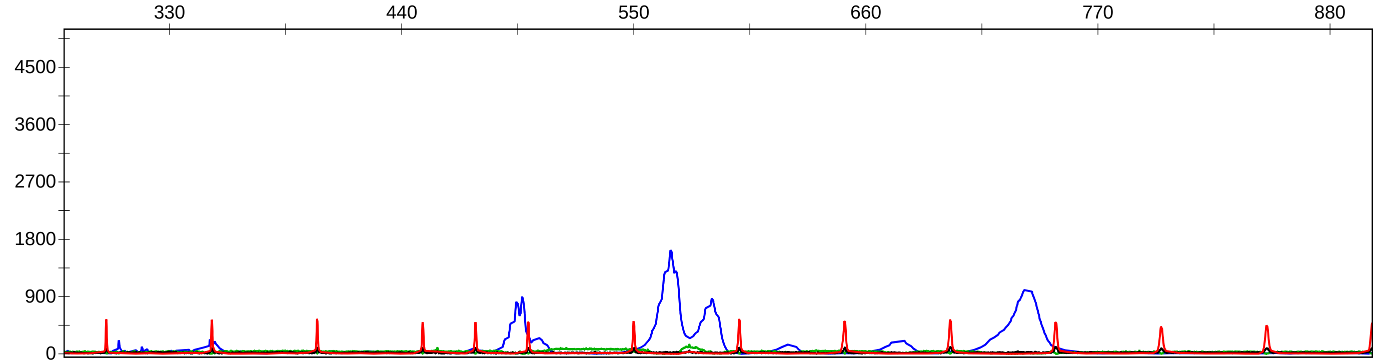

Sample File	Sample Name	Panel	OS	SQ
7-07-PM15_G01.fsa	7-07-PM15	R62_150-1500		

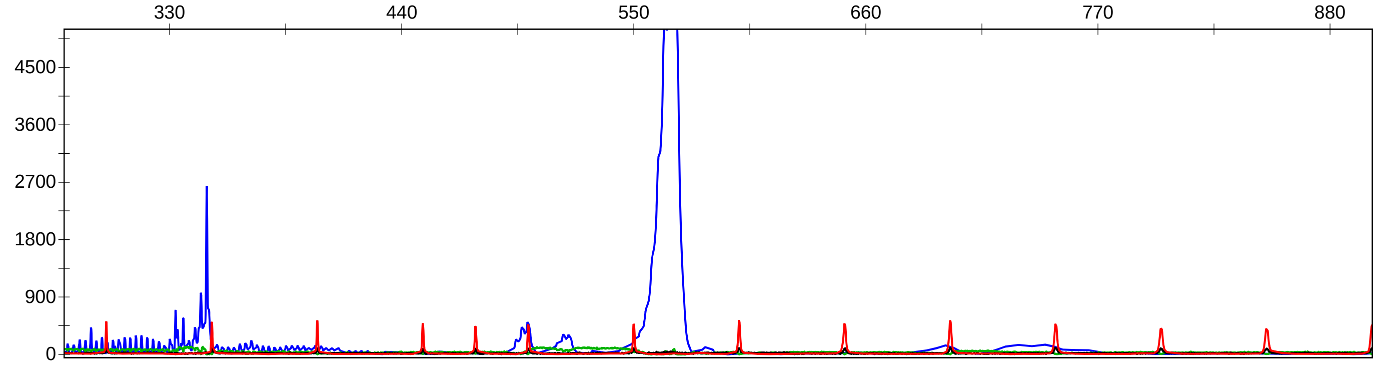

Sample File	Sample Name	Panel	OS	SQ
7-08-PM16_H01.fsa	7-08-PM16	R62_150-1500		

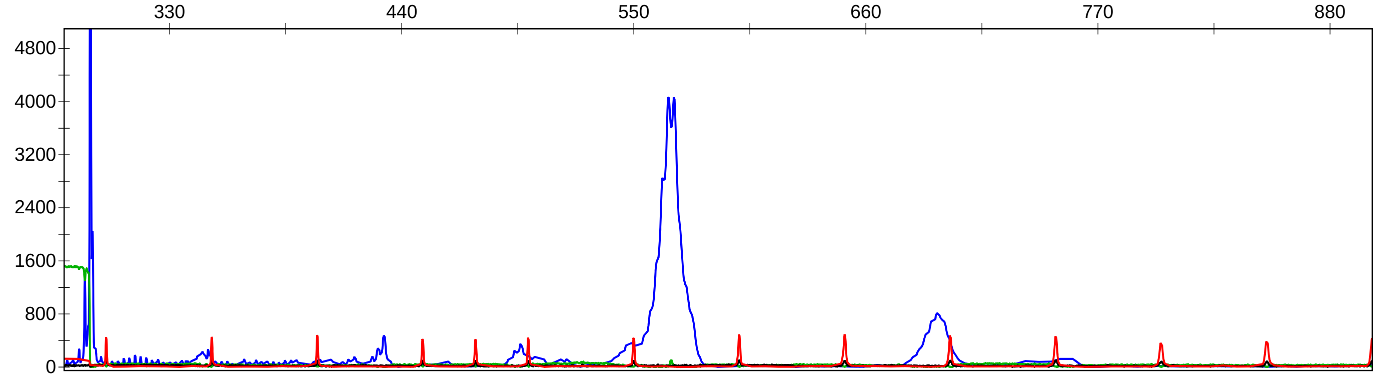

Sample File	Sample Name	Panel	OS	SQ
7-09-PM17_A02.fsa	7-09-PM17	R62_150-1500		

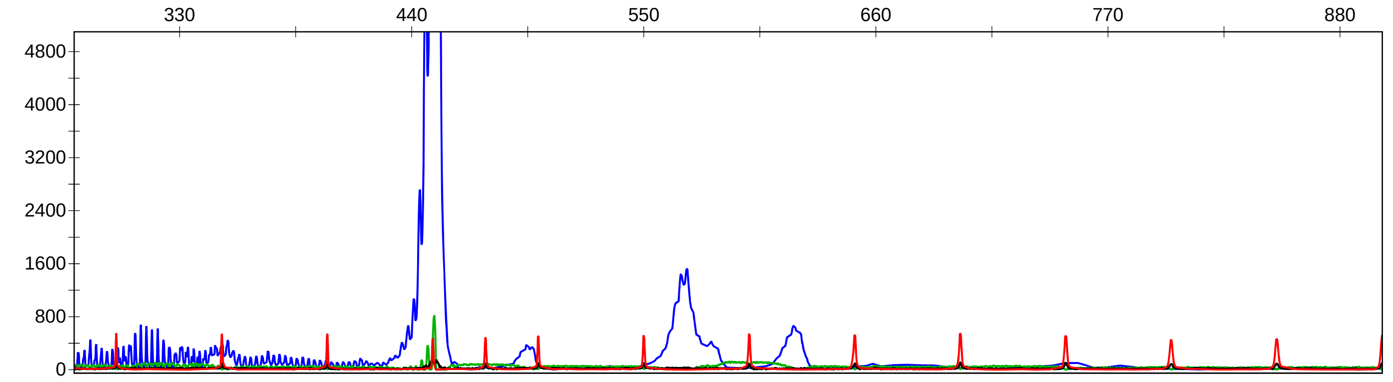

Sample File	Sample Name	Panel	OS	SQ
7-10-PM18_B02.fsa	7-10-PM18	R62_150-1500		

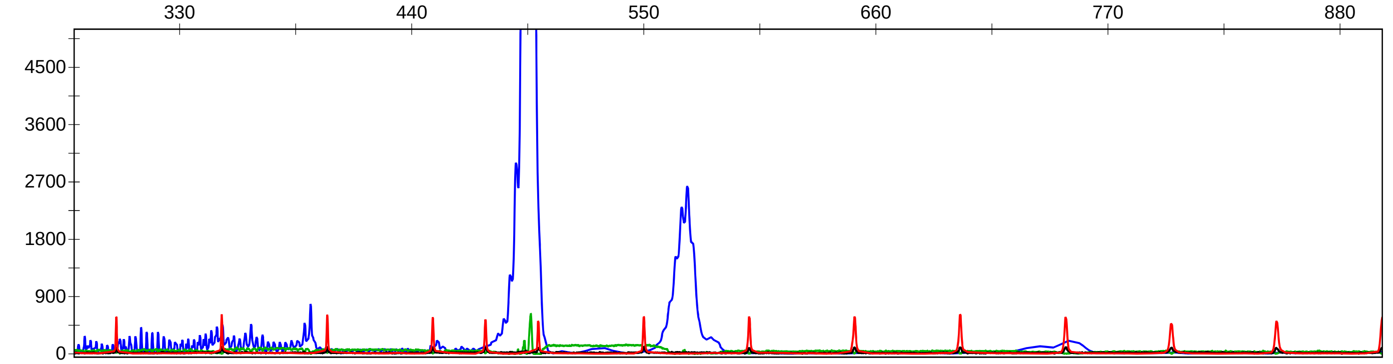

Sample File	Sample Name	Panel	OS	SQ
7-11-PM19_C02.fsa	7-11-PM19	R62_150-1500		

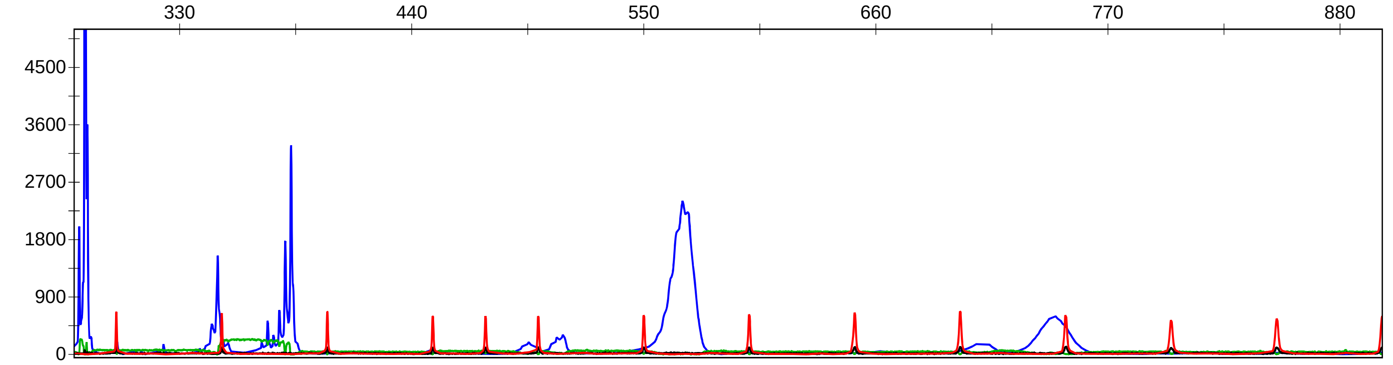

Sample File	Sample Name	Panel	OS	SQ
7-12-PM20_D02.fsa	7-12-PM20	R62_150-1500		

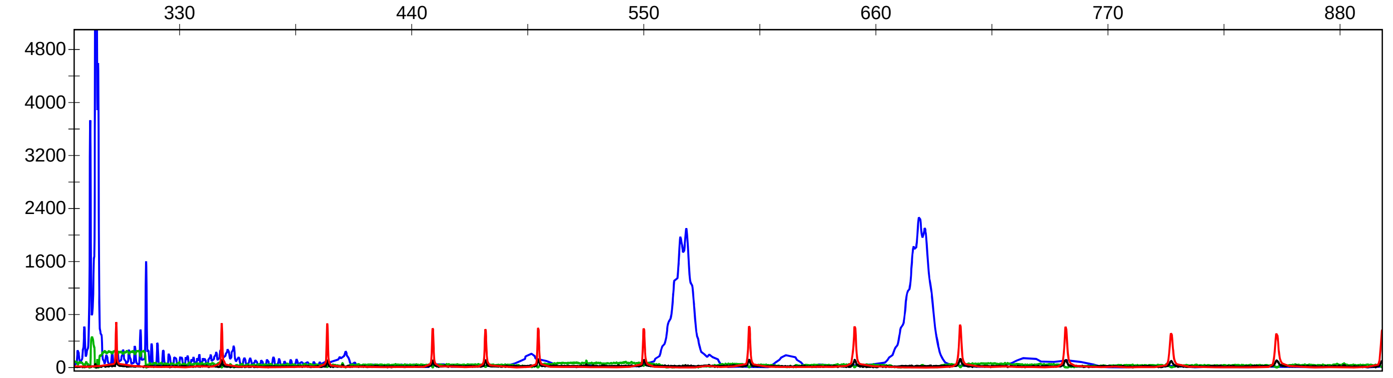

Sample File	Sample Name	Panel	OS	SQ
7-13-PM21_E02.fsa	7-13-PM21	R62_150-1500		

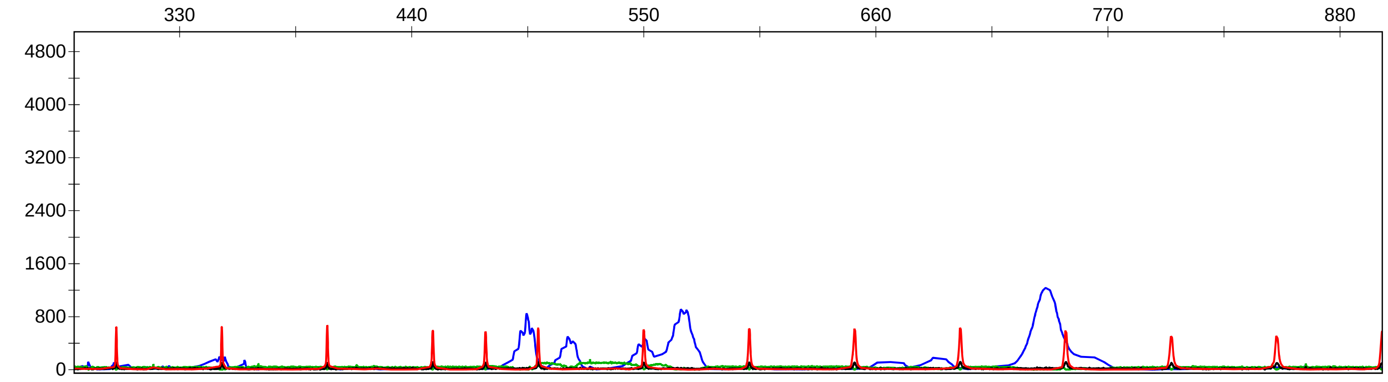

Sample File	Sample Name	Panel	OS	SQ
7-14-PM22_F02.fsa	7-14-PM22	R62_150-1500		

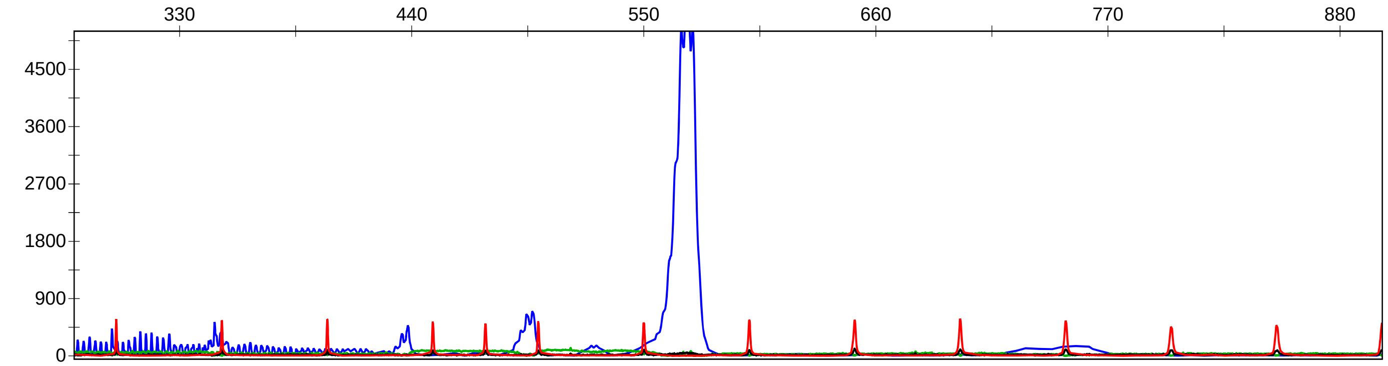

Sample File	Sample Name	Panel	OS	SQ
7-15-PM23_G02.fsa	7-15-PM23	R62_150-1500		

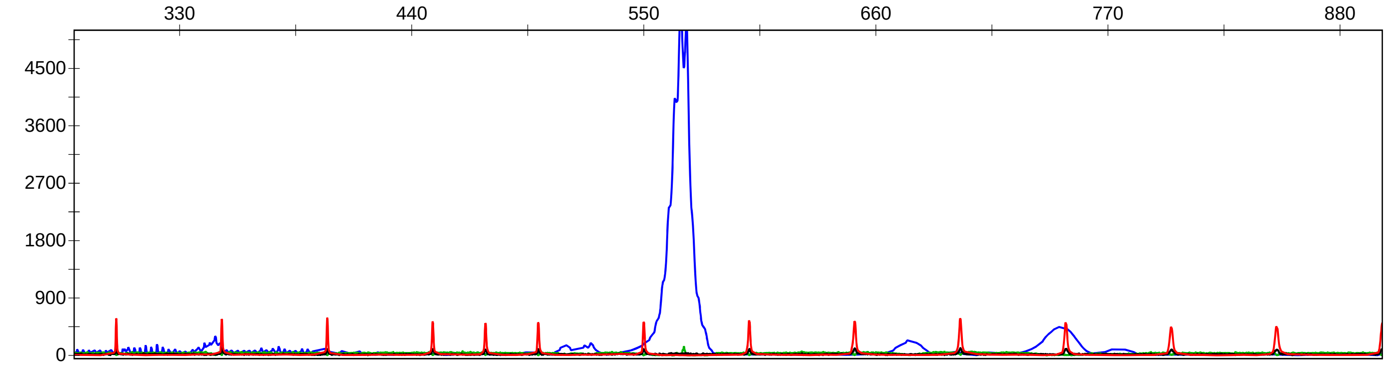

Sample File	Sample Name	Panel	OS	SQ
7-16-PM24_H02.fsa	7-16-PM24	R62_150-1500		

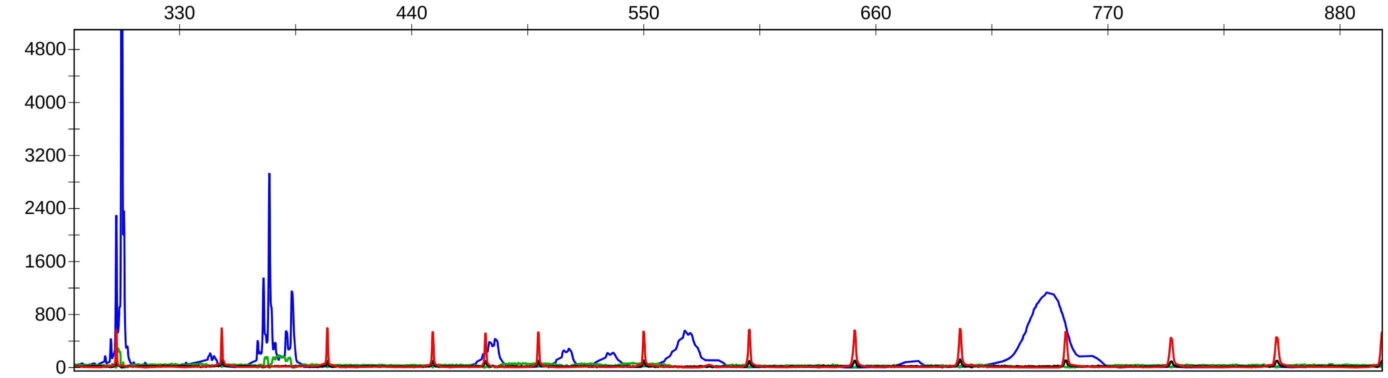

Applied Biosystems

GeneMapper Software 5

20180922CGG

Sample File	Sample Name	Panel	OS	SQ
7-17-PM25_A03.fsa	7-17-PM25	R62_150-1500		

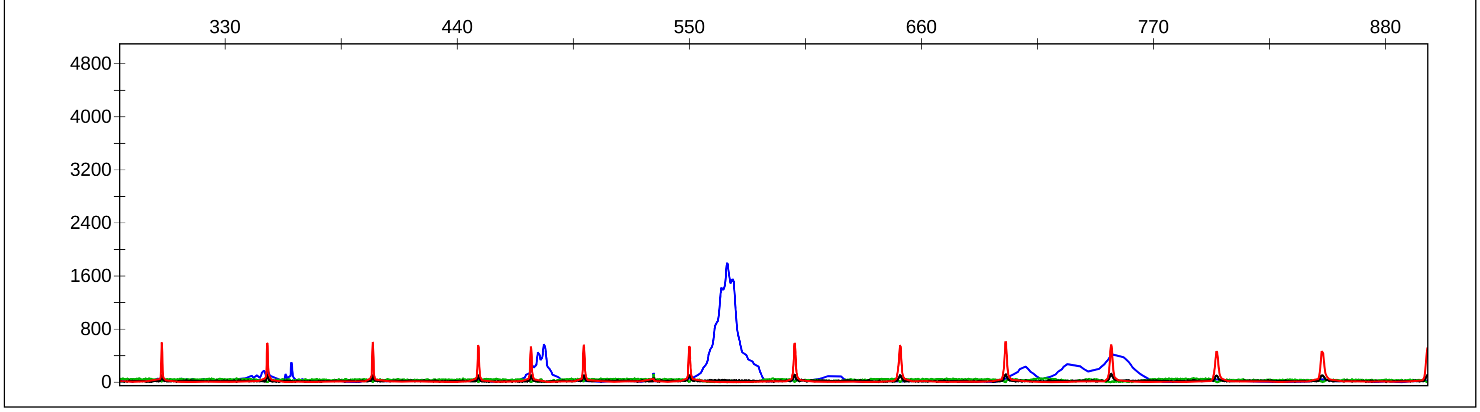

Sample File	Sample Name	Panel	OS	SQ
7-18-PM26_B03.fsa	7-18-PM26	R62_150-1500		

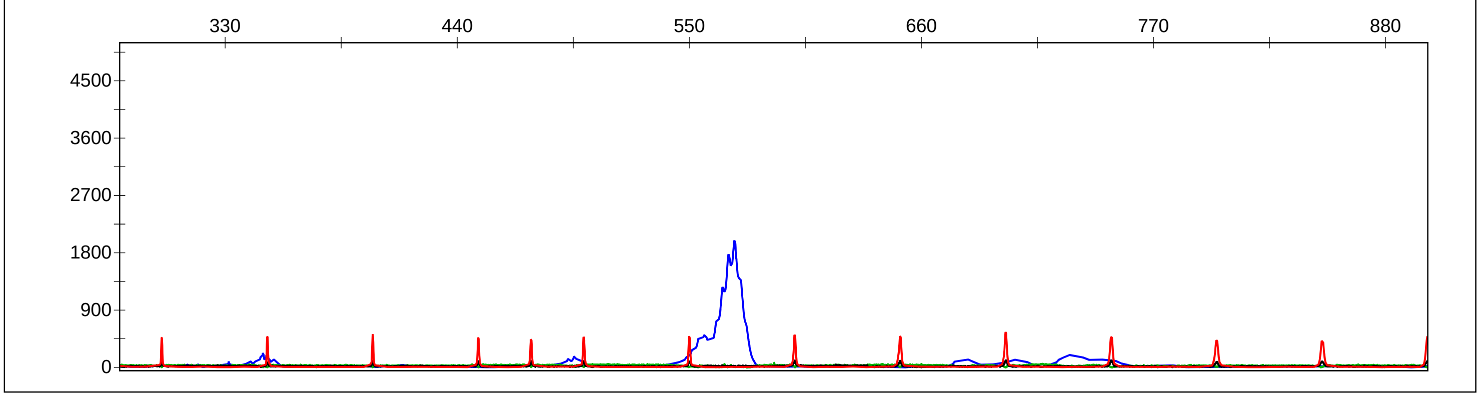

Sample File	Sample Name	Panel	OS	SQ
7-19-PM27_C03.fsa	7-19-PM27	R62_150-1500		

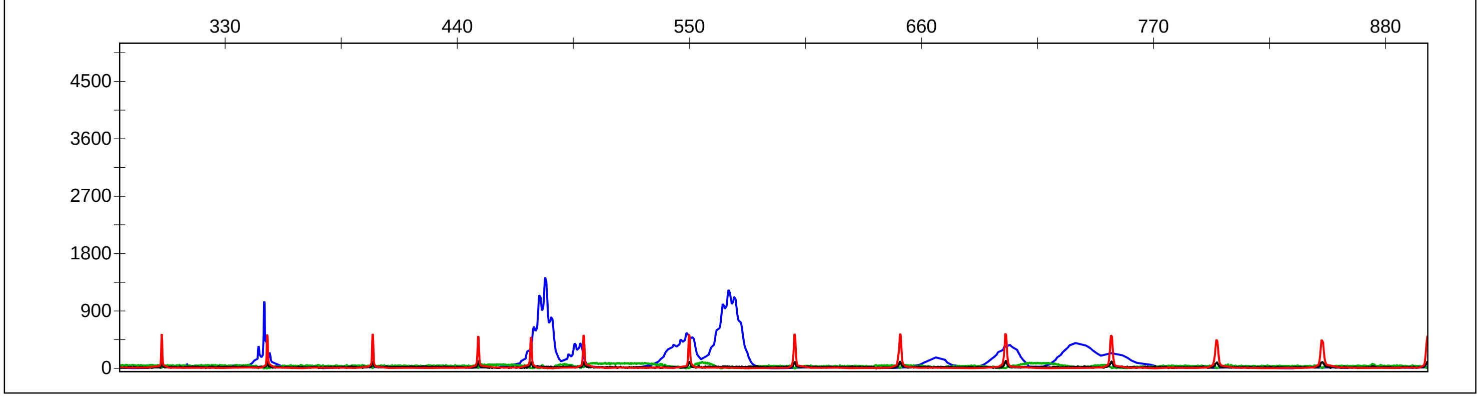

Sample File	Sample Name	Panel	OS	SQ
7-20-PM28_D03.fsa	7-20-PM28	R62_150-1500		

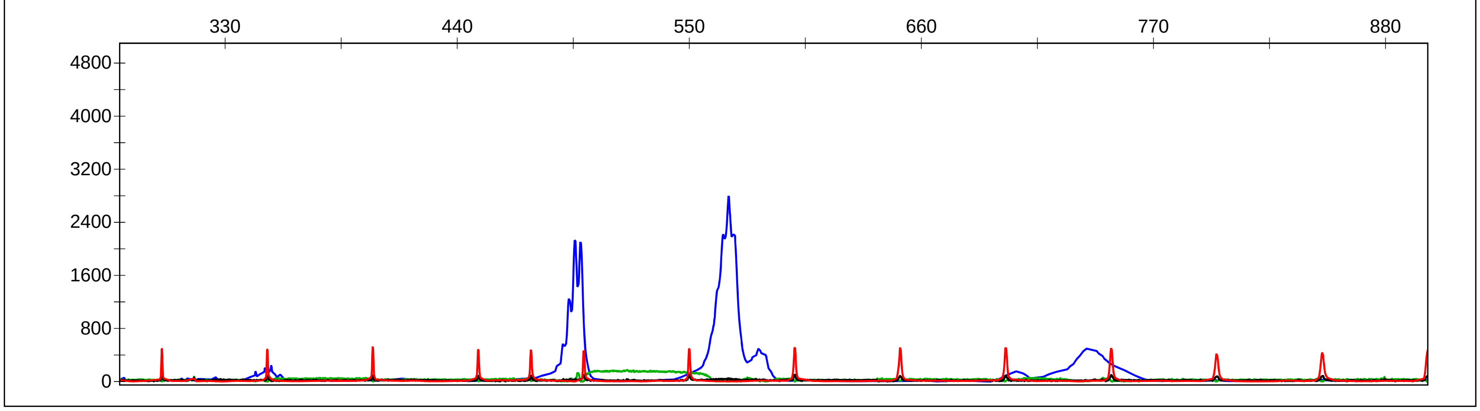

Sample File	Sample Name	Panel	OS	SQ
7-21-PM29_E03.fsa	7-21-PM29	R62_150-1500		

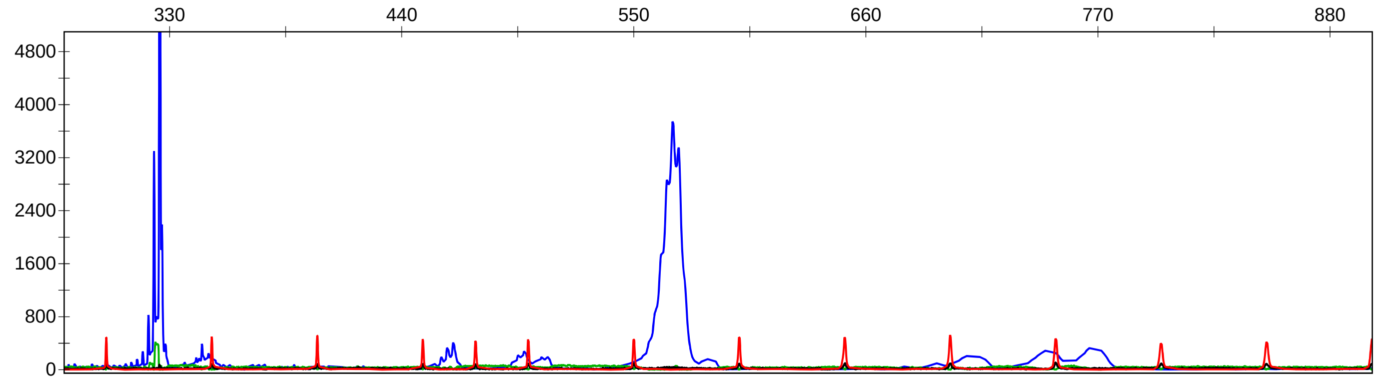

Sample File	Sample Name	Panel	OS	SQ
7-22-PM30_F03.fsa	7-22-PM30	R62_150-1500		

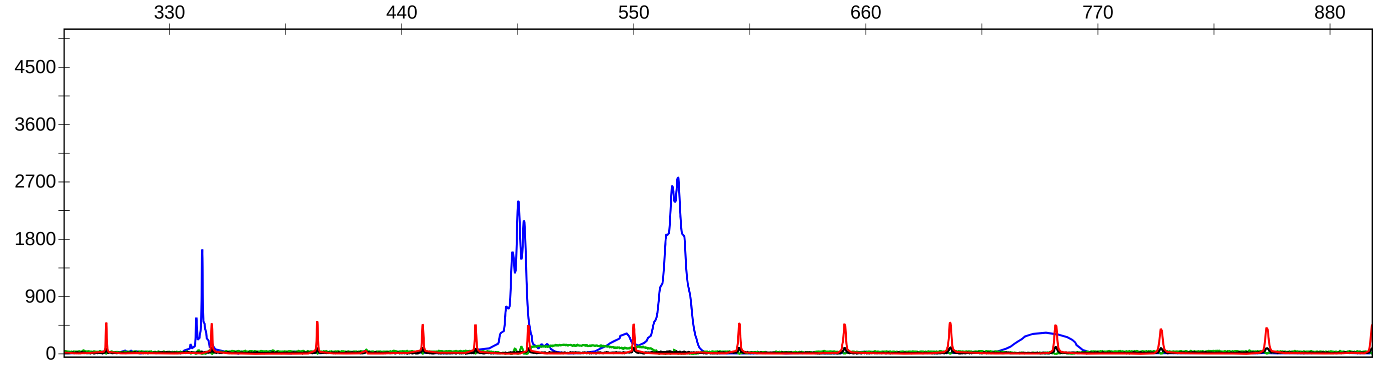

Sample File	Sample Name	Panel	OS	SQ
7-23-PM31_G03.fsa	7-23-PM31	R62_150-1500		

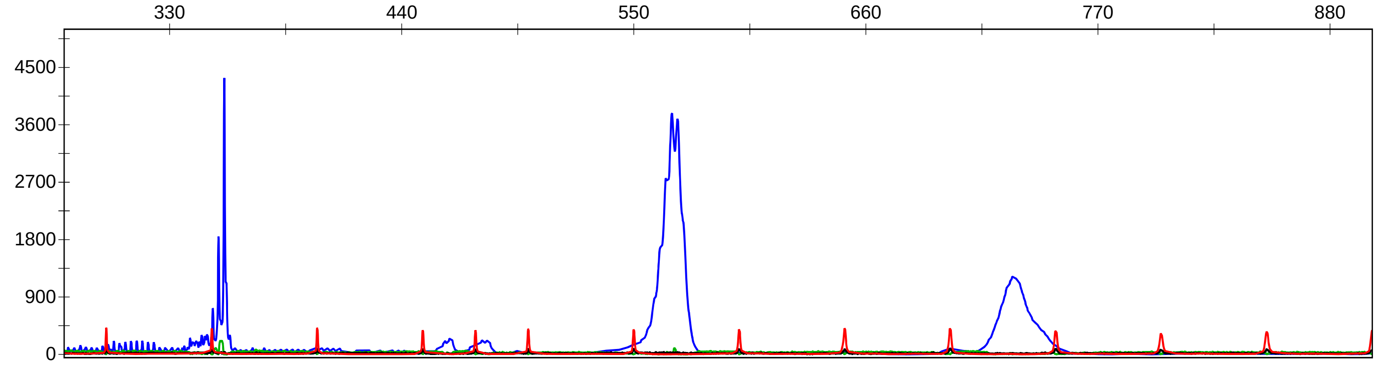

Sample File	Sample Name	Panel	OS	SQ
7-24-PM32_H03.fsa	7-24-PM32	R62_150-1500		

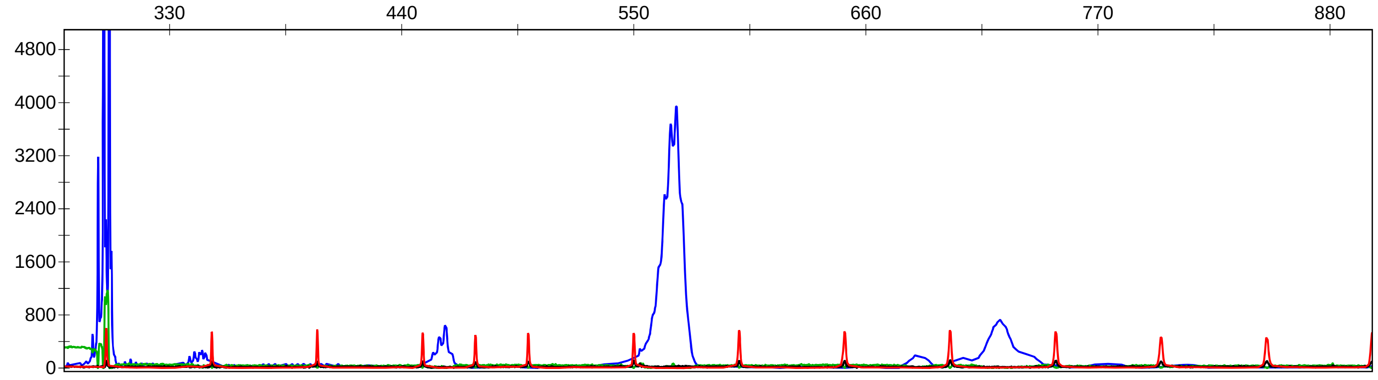

Applied Biosystems
GeneMapper Software 5

20180922CGG

Sample File	Sample Name	Panel	OS	SQ
7-25-PM33_A04.fsa	7-25-PM33	R62_150-1500		

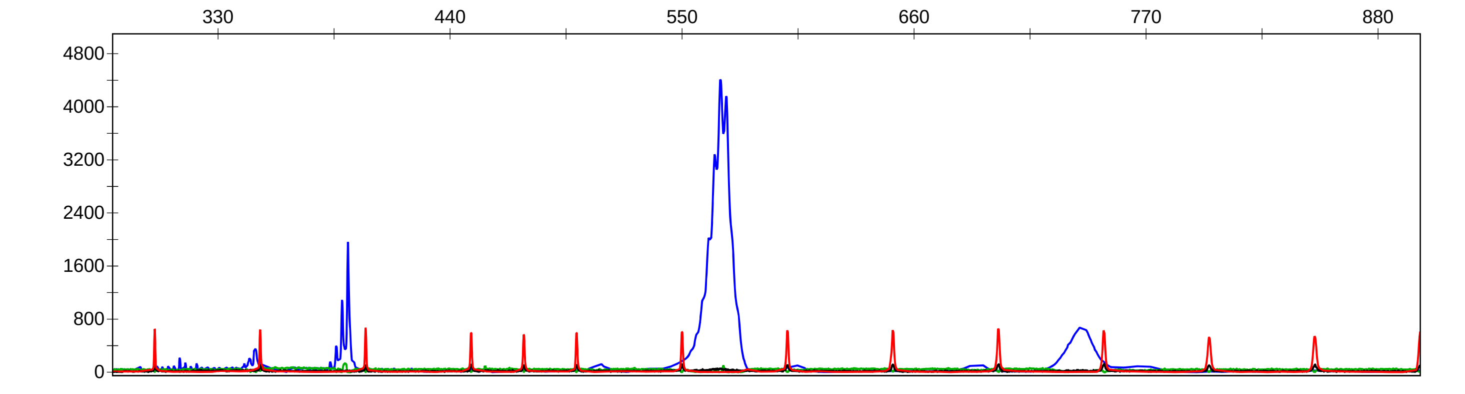

Sample File	Sample Name	Panel	OS	SQ
7-26-PM34_B04.fsa	7-26-PM34	R62_150-1500		

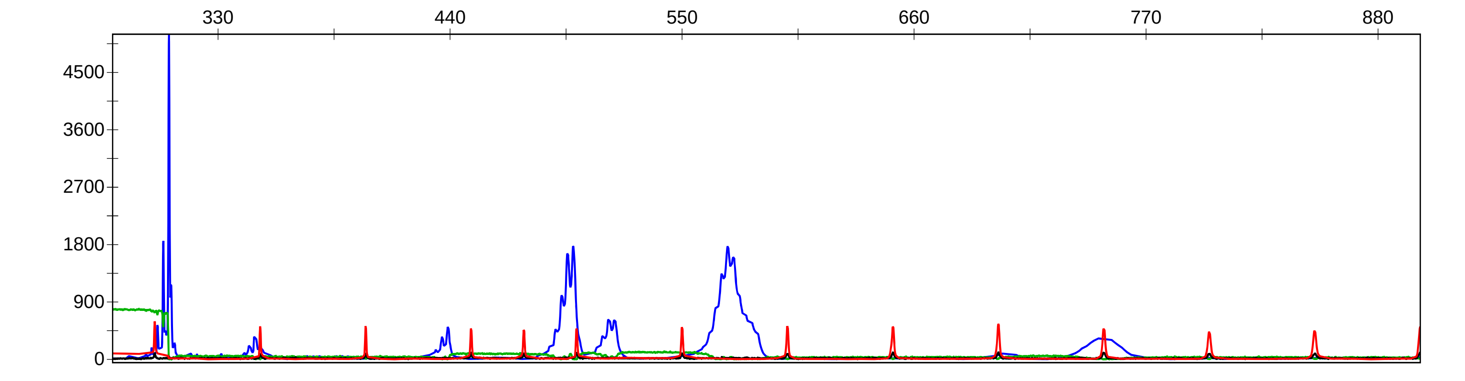

Sample File	Sample Name	Panel	OS	SQ
7-27-PM35_C04.fsa	7-27-PM35	R62_150-1500		

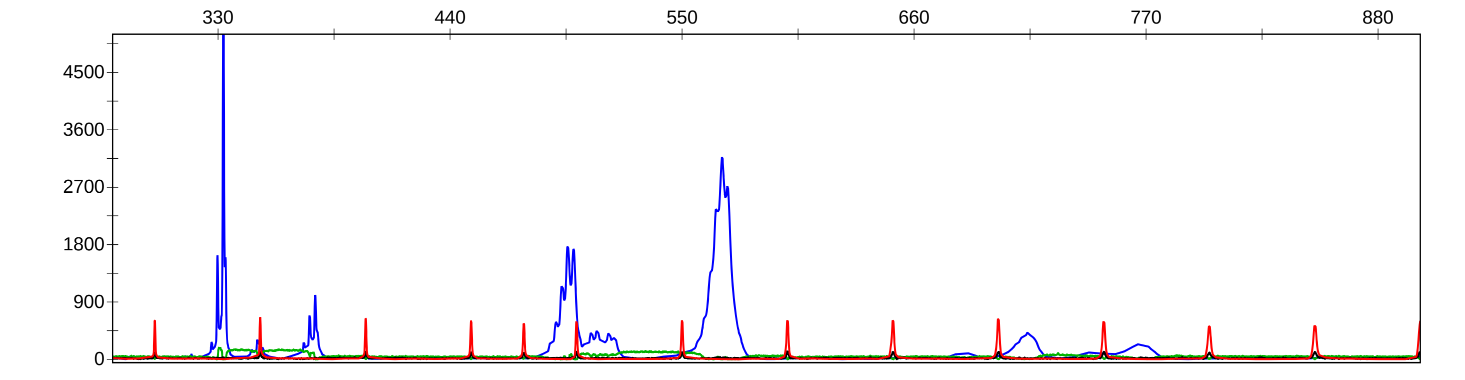

Sample File	Sample Name	Panel	OS	SQ
7-28-PM36_D04.fsa	7-28-PM36	R62_150-1500		

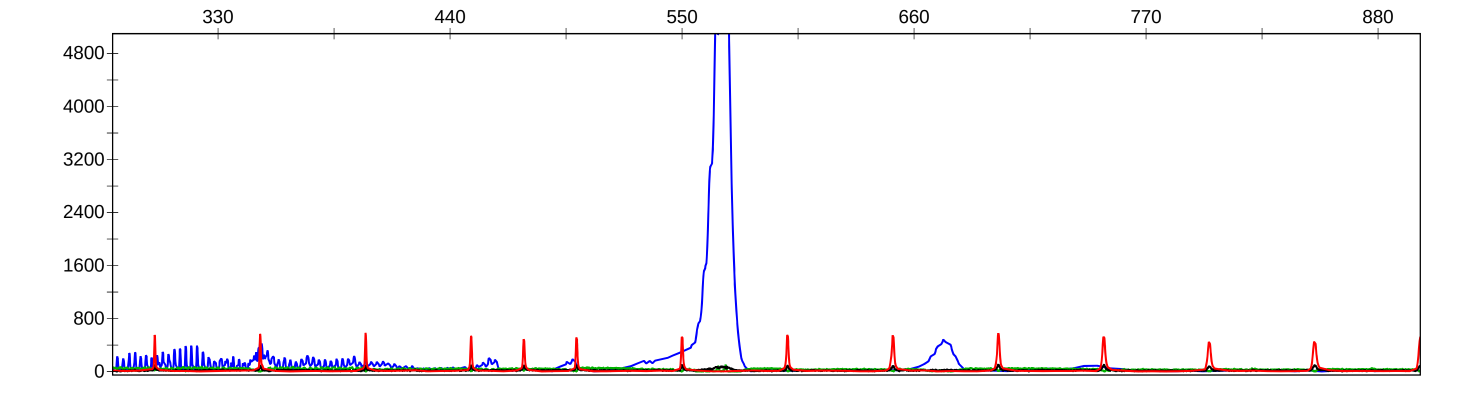

Sample File	Sample Name	Panel	OS	SQ
7-29-PM37_E04.fsa	7-29-PM37	R62_150-1500		

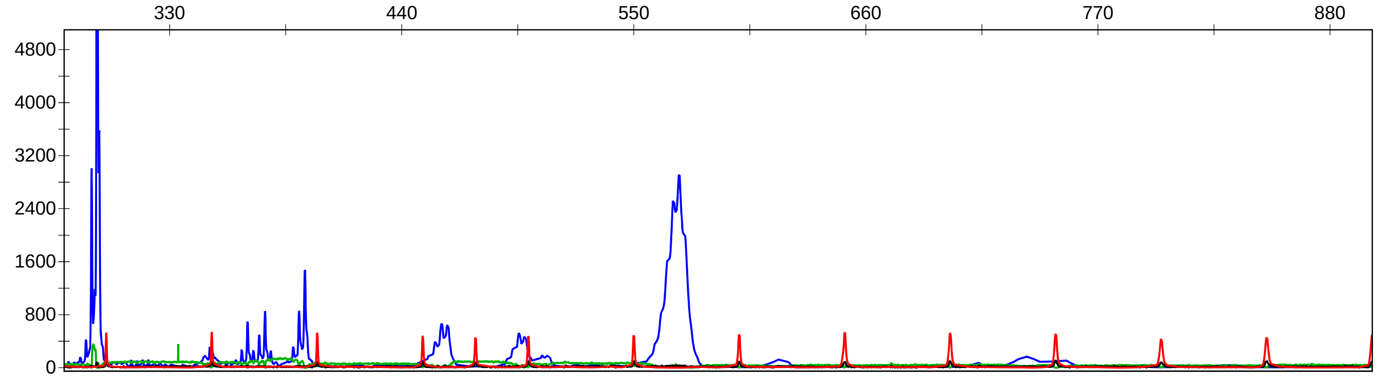

Sample File	Sample Name	Panel	OS	SQ
7-30-PM38_F04.fsa	7-30-PM38	R62_150-1500		

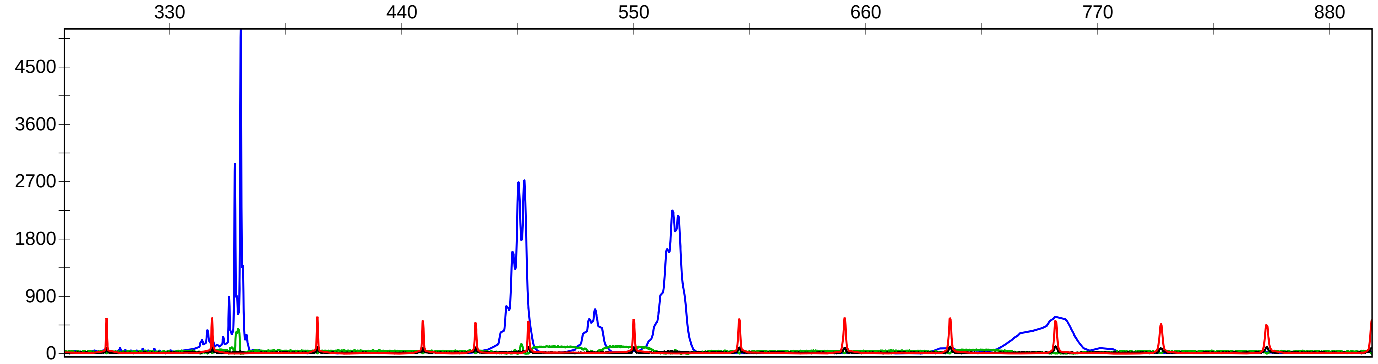

Sample File	Sample Name	Panel	OS	SQ
7-31-PM39_G04.fsa	7-31-PM39	R62_150-1500		

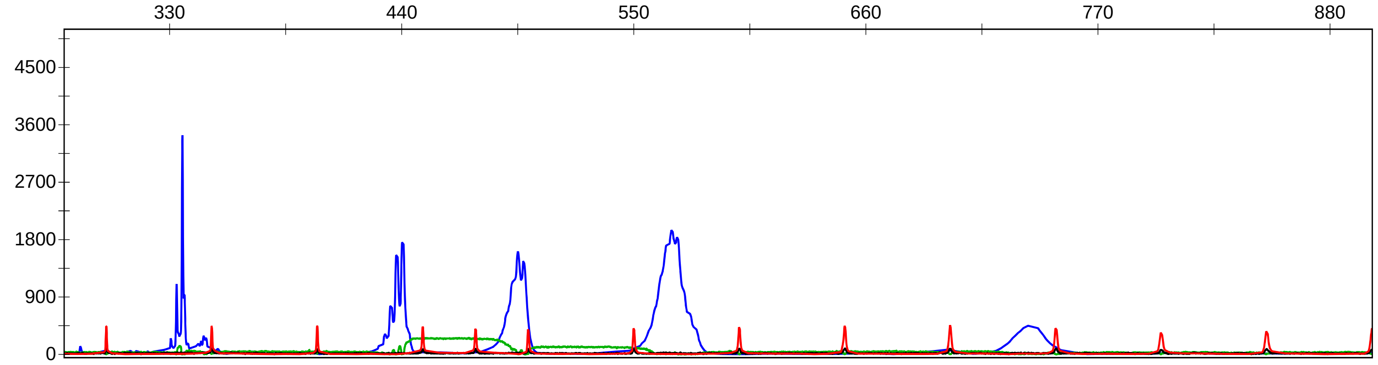

Sample File	Sample Name	Panel	OS	SQ
7-32-PM40_H04.fsa	7-32-PM40	R62_150-1500		

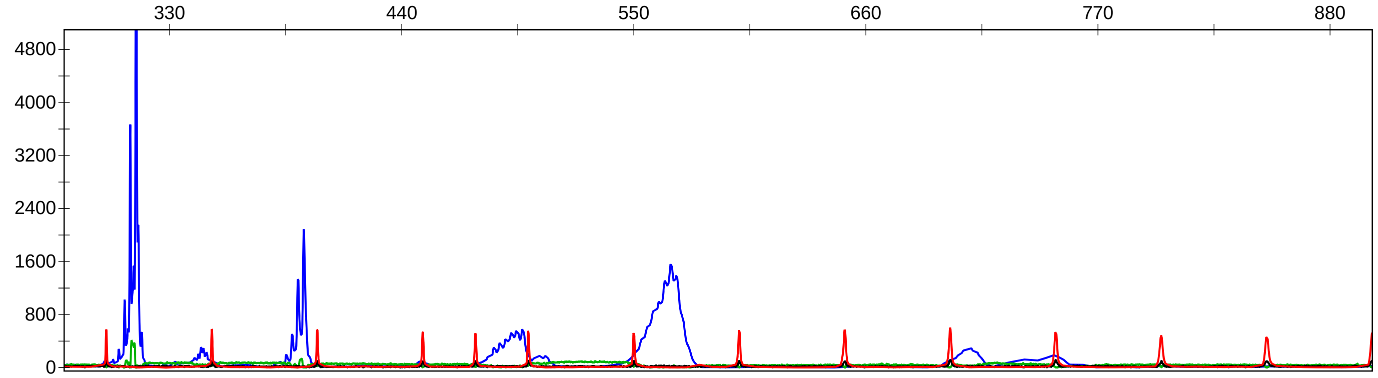

Applied Biosystems

GeneMapper Software 5

20180922CGG

Sample File	Sample Name	Panel	OS	SQ
7-33-PM41_A05.fsa	7-33-PM41	R62_150-1500		

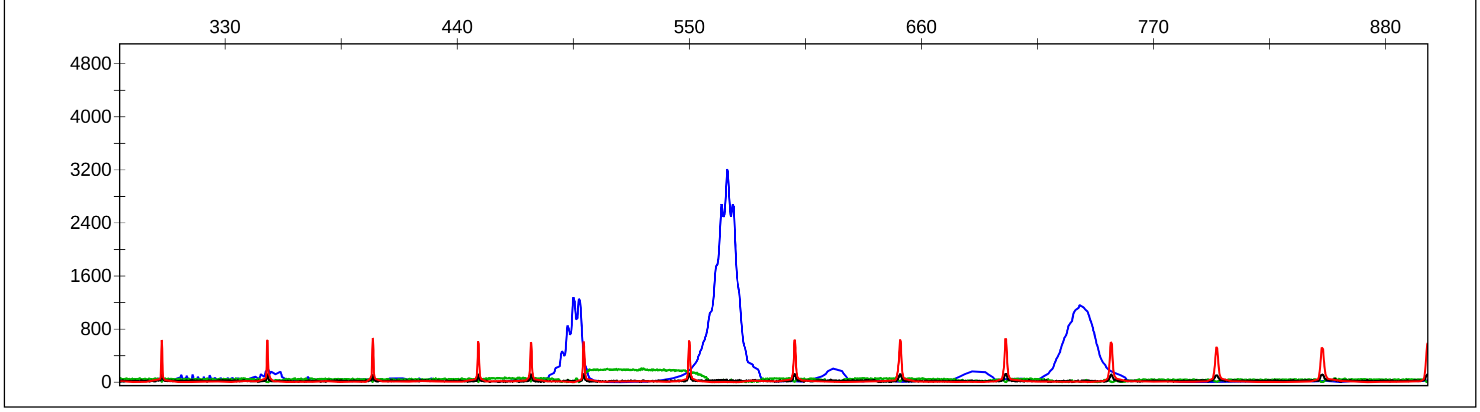

7-34-PM42_B05.fsa	7-34-PM42	R62_150-1500		

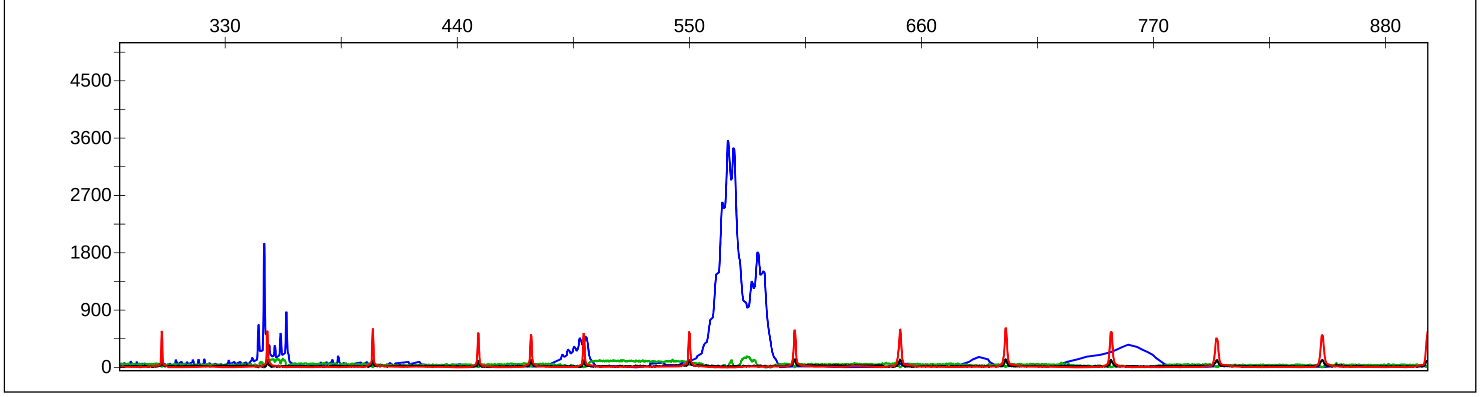

7-35-PM43_C05.fsa	7-35-PM43	R62_150-1500		

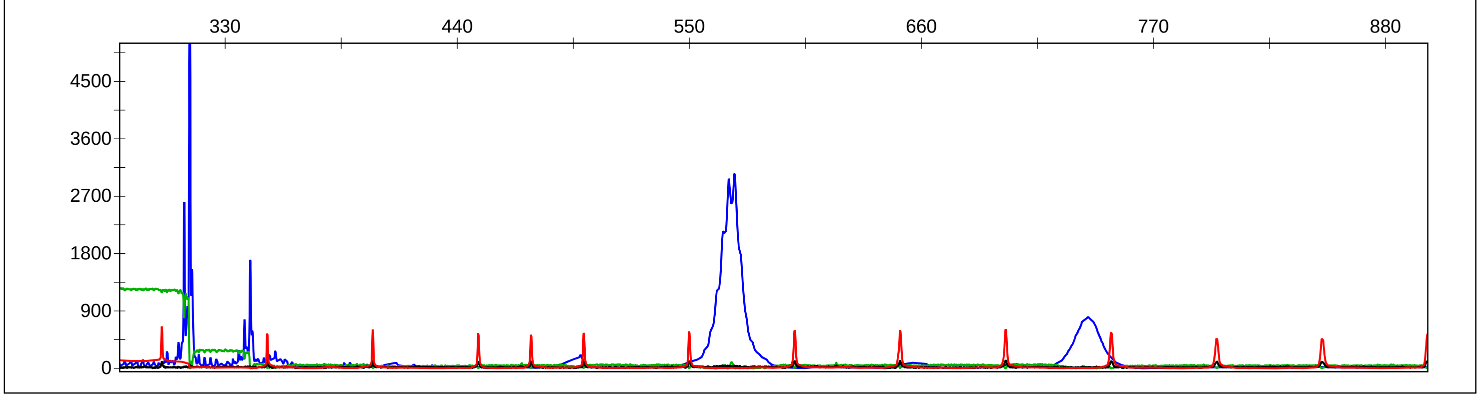

7-36-PM44_D05.fsa	7-36-PM44	R62_150-1500		

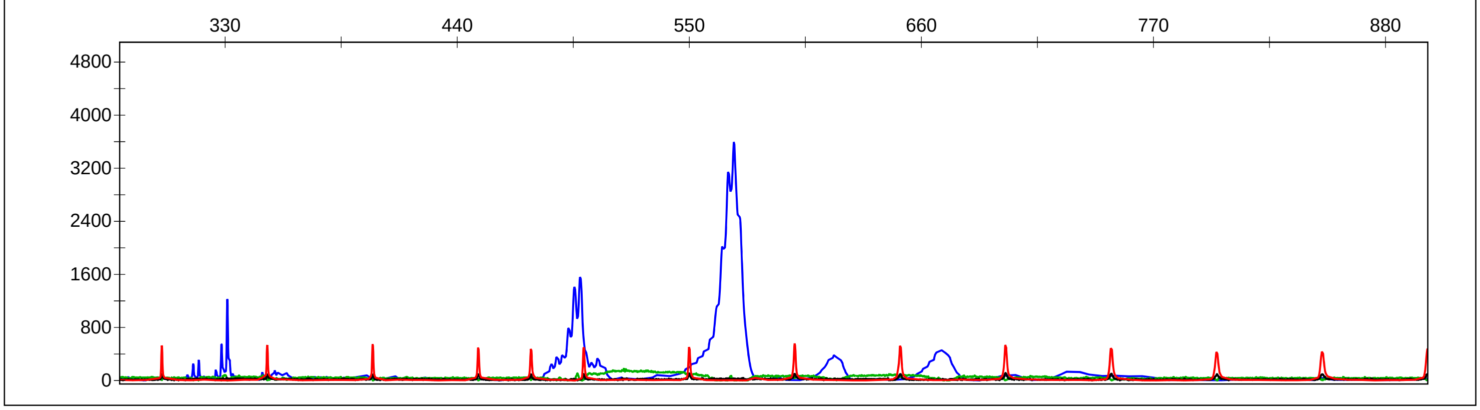

Applied Biosystems

GeneMapper Software 5

20180922CGG

Sample File	Sample Name	Panel	OS	SQ
7-37-PM45_E05.fsa	7-37-PM45	R62_150-1500		

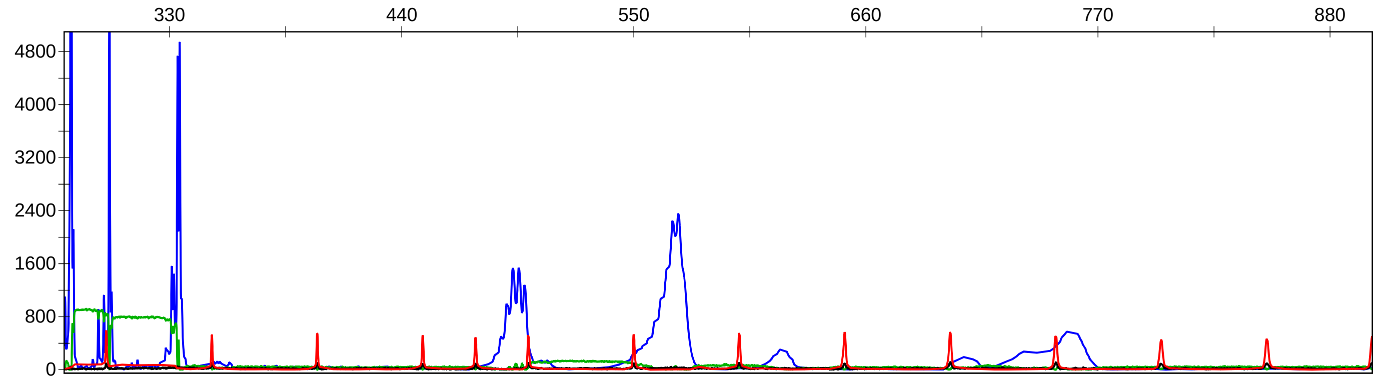

Sample File	Sample Name	Panel	OS	SQ
7-38-PM46_F05.fsa	7-38-PM46	R62_150-1500		

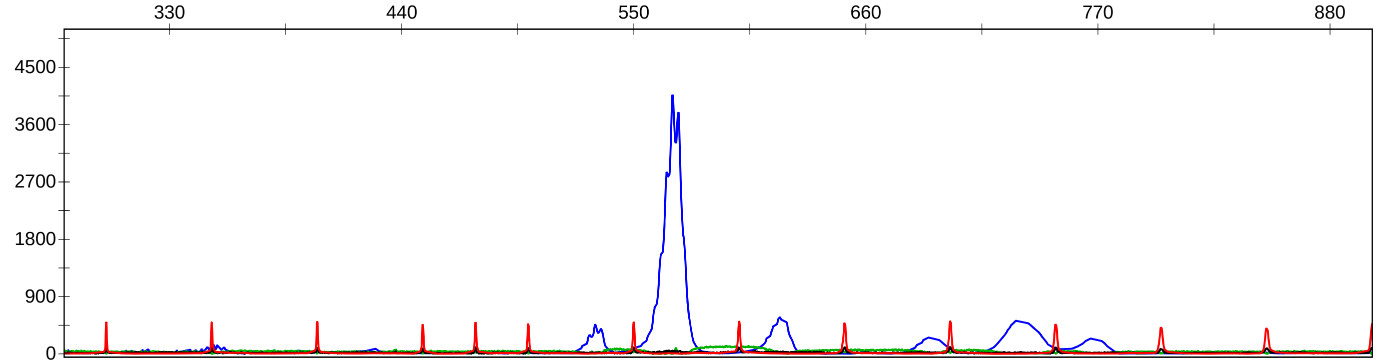

Sample File	Sample Name	Panel	OS	SQ
7-39-PM47_G05.fsa	7-39-PM47	R62_150-1500		

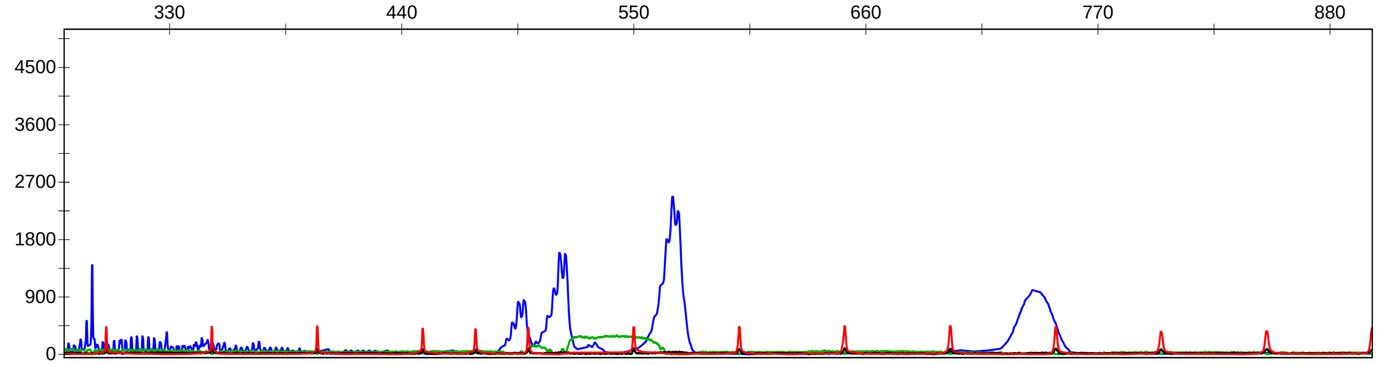

Sample File	Sample Name	Panel	OS	SQ
7-40-PM48_H05.fsa	7-40-PM48	R62_150-1500		

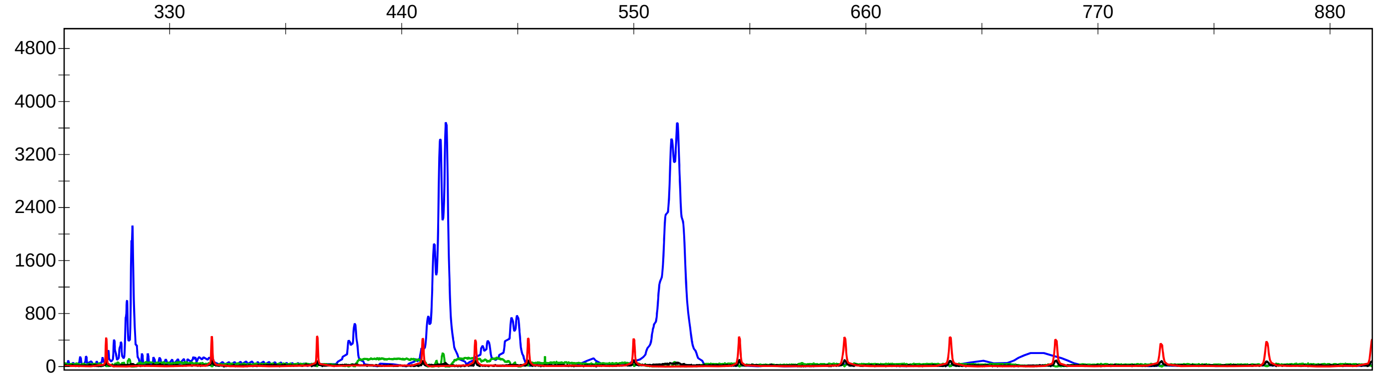

Applied Biosystems

GeneMapper Software 5

20180922CGG

Sample File	Sample Name	Panel	OS	SQ
7-41-PM49_A06.fsa	7-41-PM49	R62_150-1500		

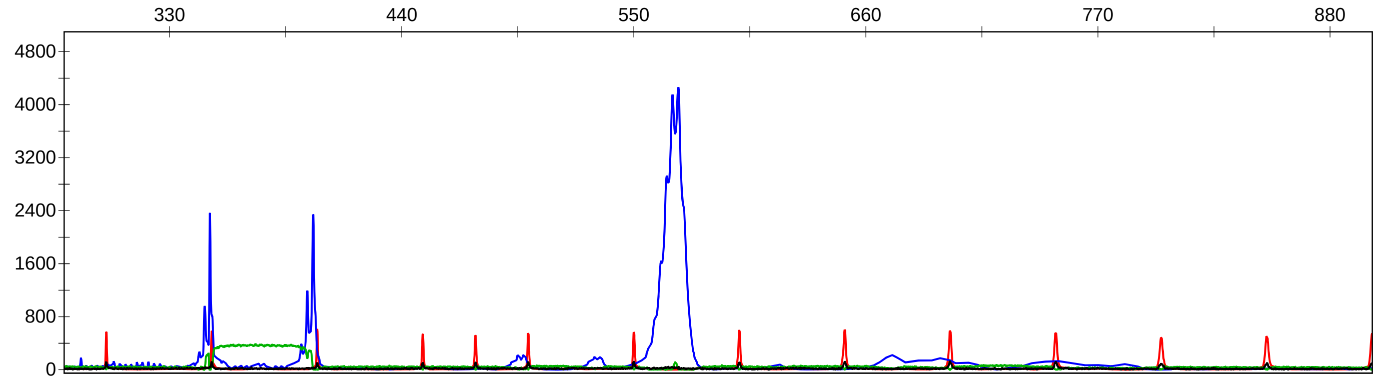

7-42-PM50_B06.fsa	7-42-PM50	R62_150-1500		

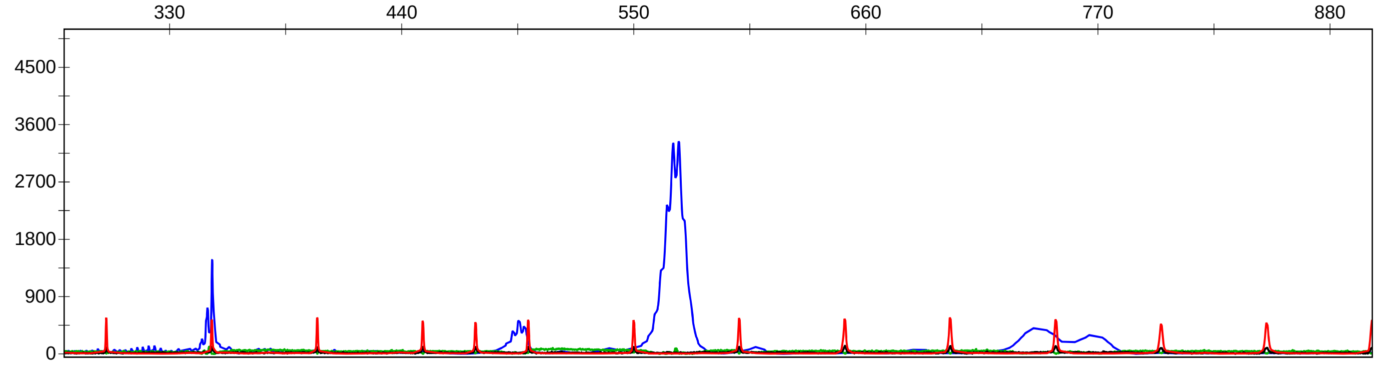

7-43-PM51_C06.fsa	7-43-PM51	R62_150-1500		

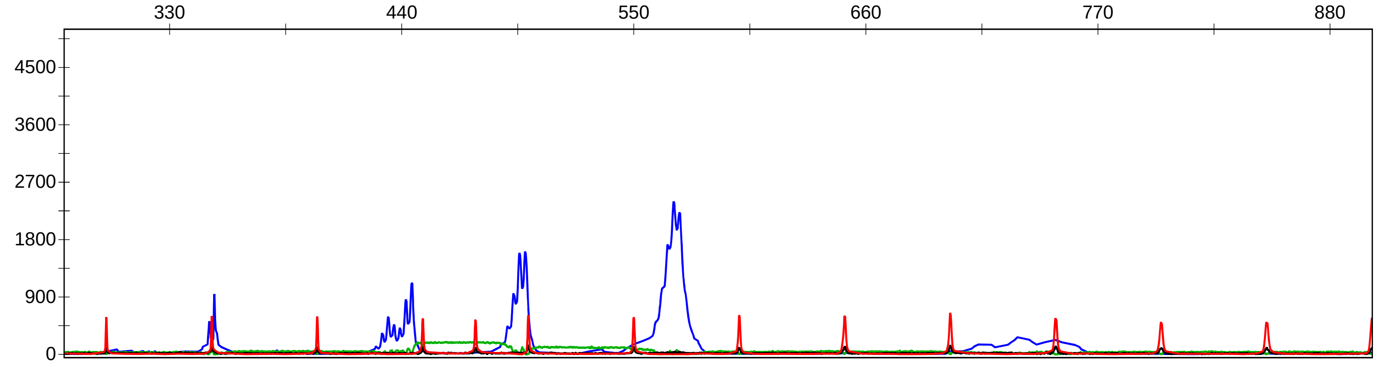

7-44-PM52_D06.fsa	7-44-PM52	R62_150-1500		

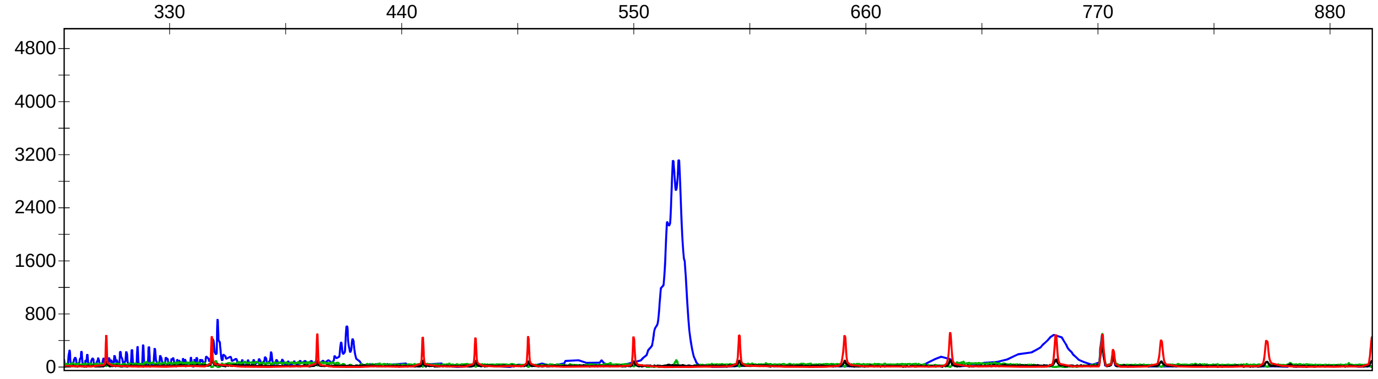

Sample File	Sample Name	Panel	OS	SQ
7-45-PM53_E06.fsa	7-45-PM53	R62_150-1500		

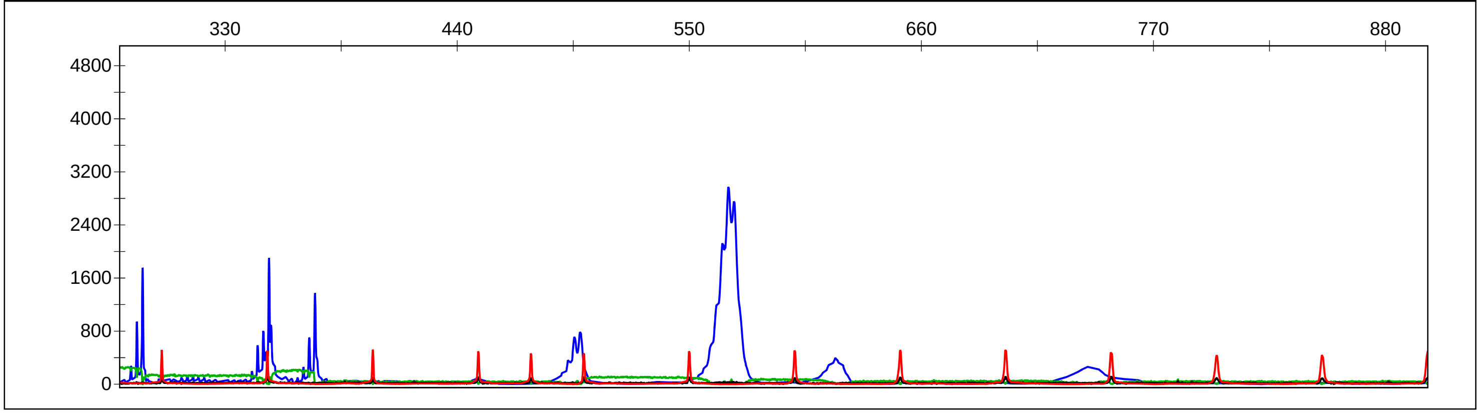

Sample File	Sample Name	Panel	OS	SQ
7-46-PM54_F06.fsa	7-46-PM54	R62_150-1500		

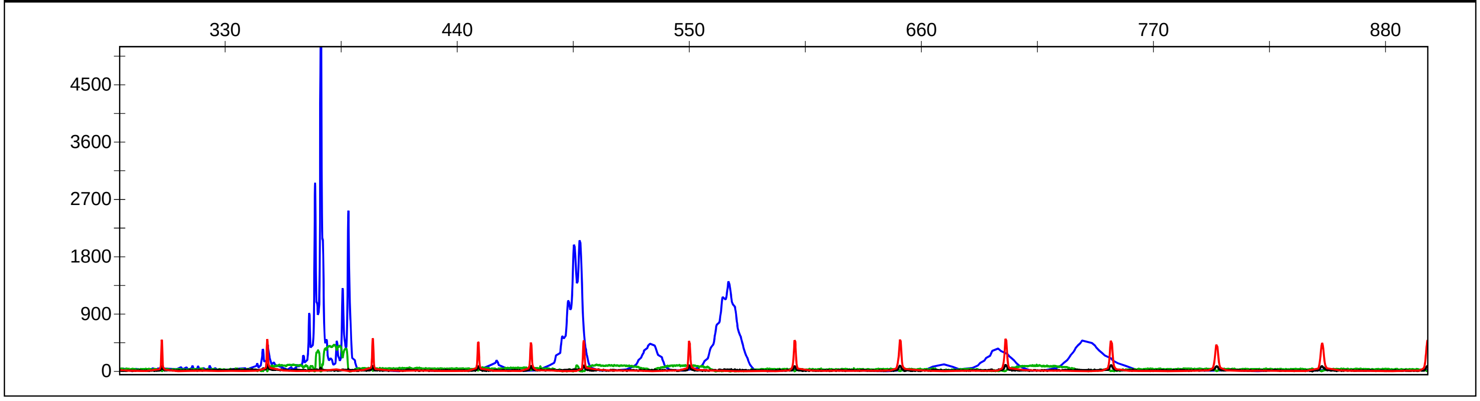

Sample File	Sample Name	Panel	OS	SQ
7-47-PM55_G06.fsa	7-47-PM55	R62_150-1500		

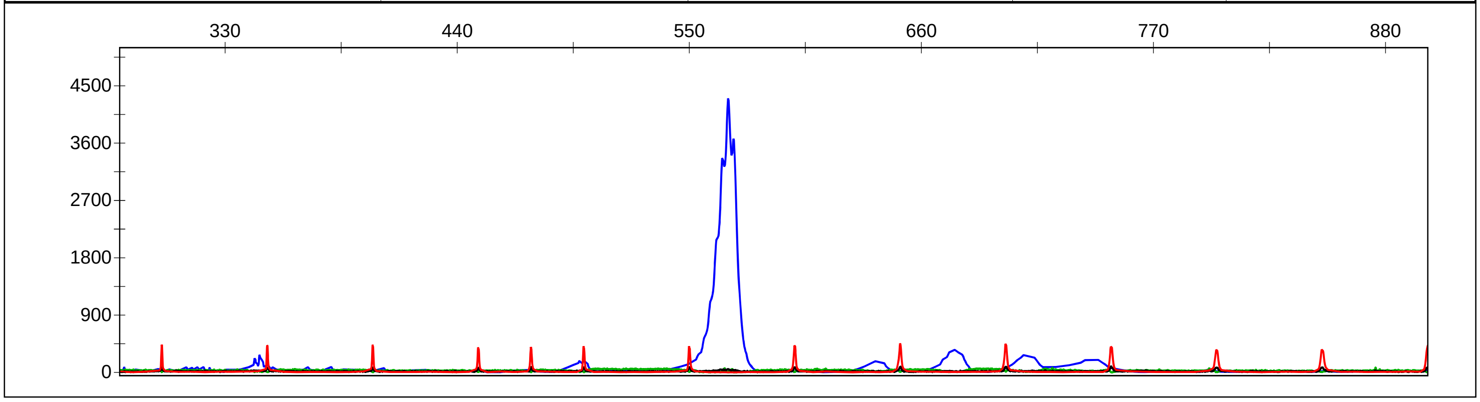

Sample File	Sample Name	Panel	OS	SQ
7-48-PM56_H06.fsa	7-48-PM56	R62_150-1500		

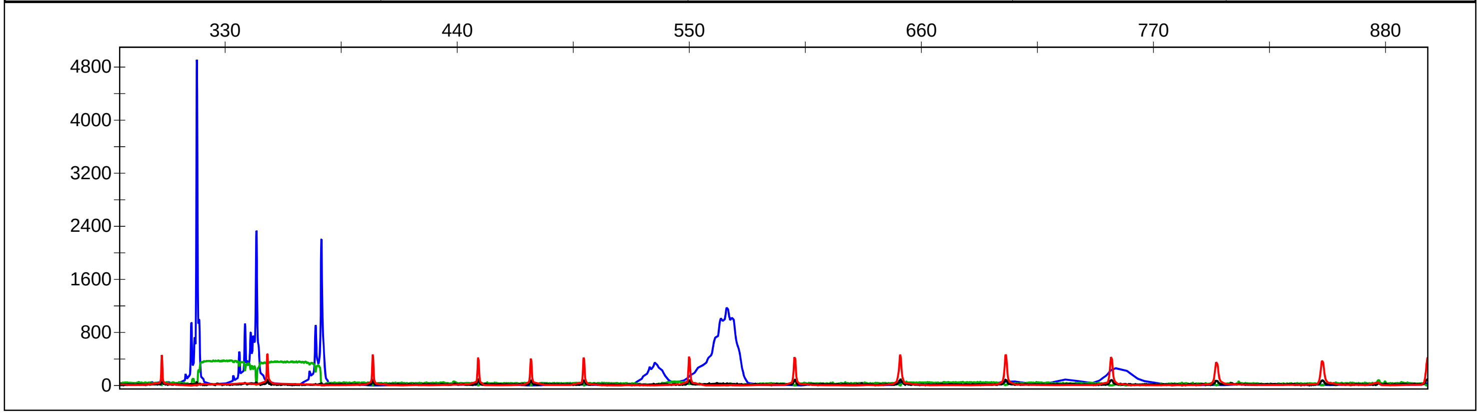

Sample File	Sample Name	Panel	OS	SQ
7-49-PM57_A07.fsa	7-49-PM57	R62_150-1500		

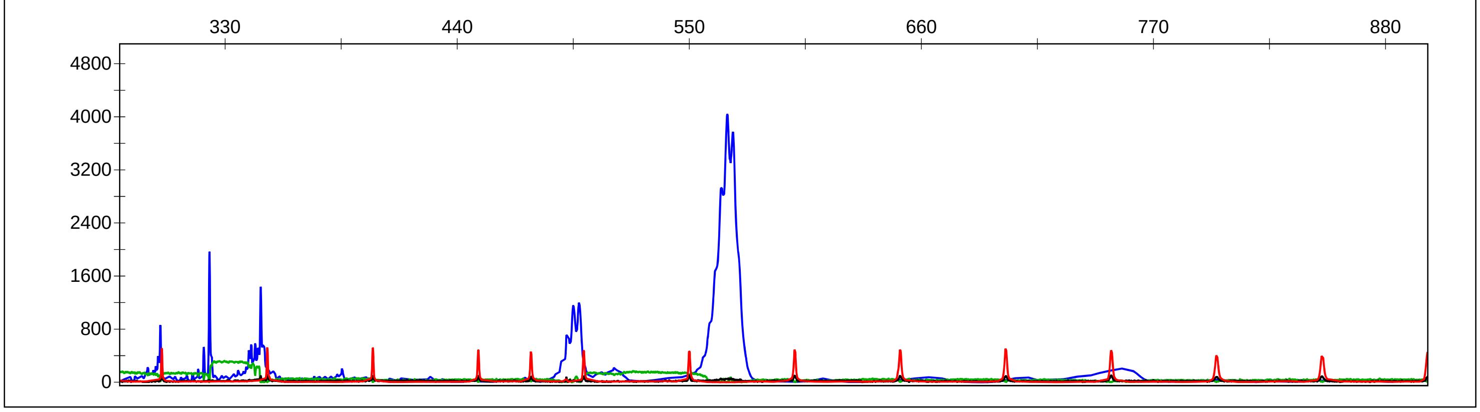

Sample File	Sample Name	Panel	OS	SQ
7-50-PM58_B07.fsa	7-50-PM58	R62_150-1500		

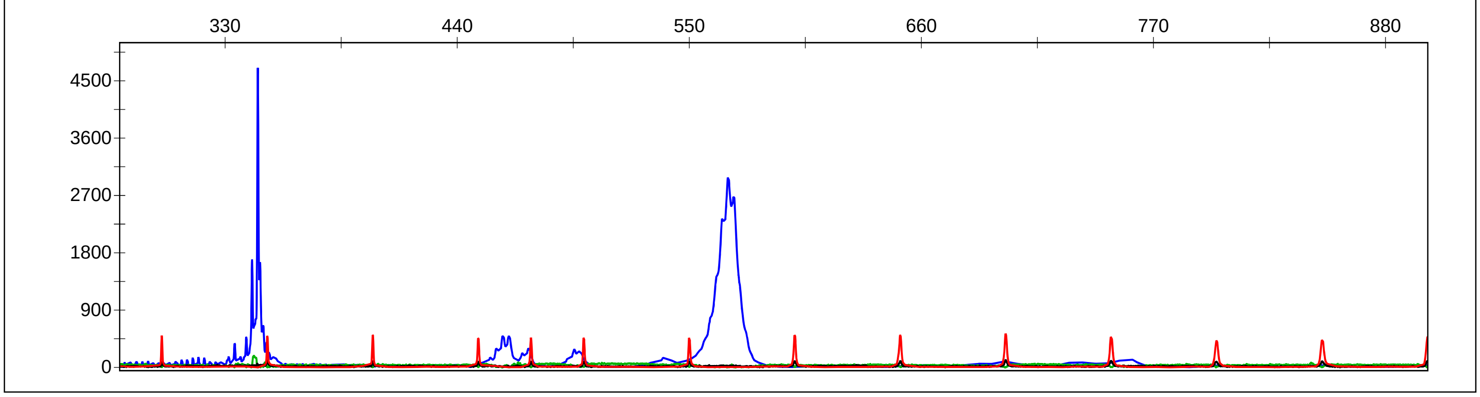

Sample File	Sample Name	Panel	OS	SQ
7-51-PM59_C07.fsa	7-51-PM59	R62_150-1500		

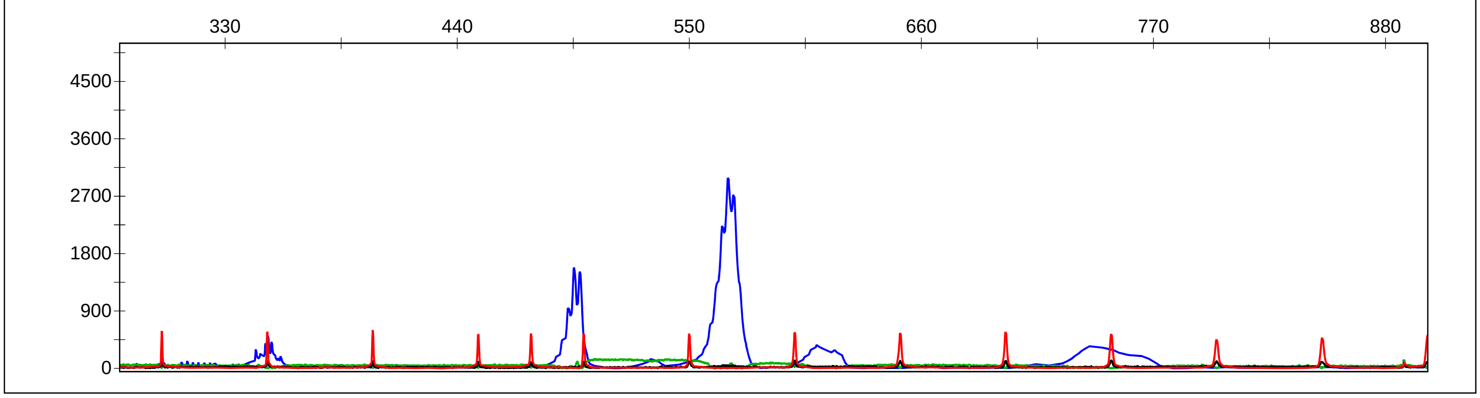

Sample File	Sample Name	Panel	OS	SQ
7-52-PM60_D07.fsa	7-52-PM60	R62_150-1500		

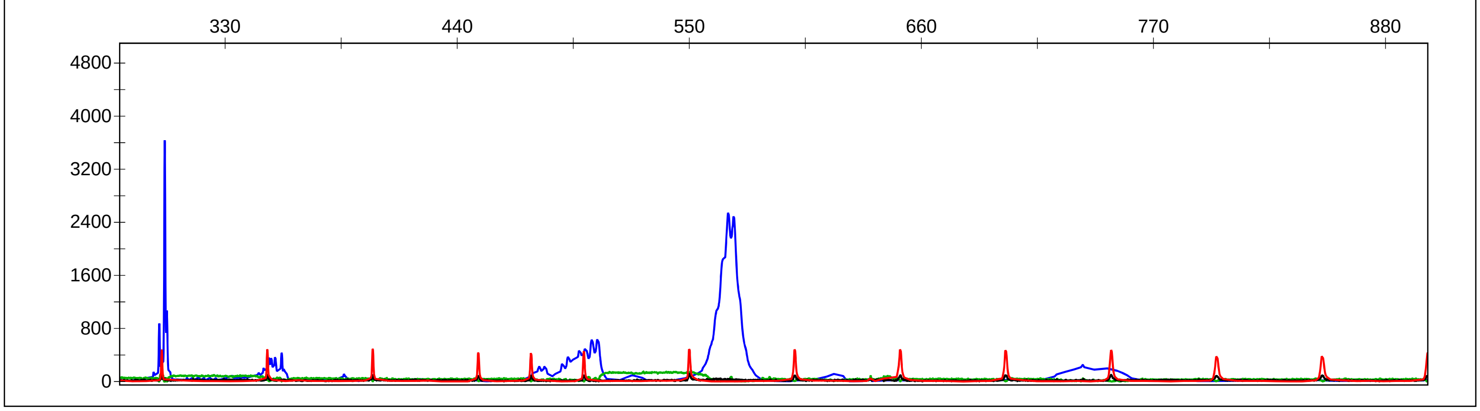

Sample File	Sample Name	Panel	OS	SQ
7-53-PM61_E07.fsa	7-53-PM61	R62_150-1500		

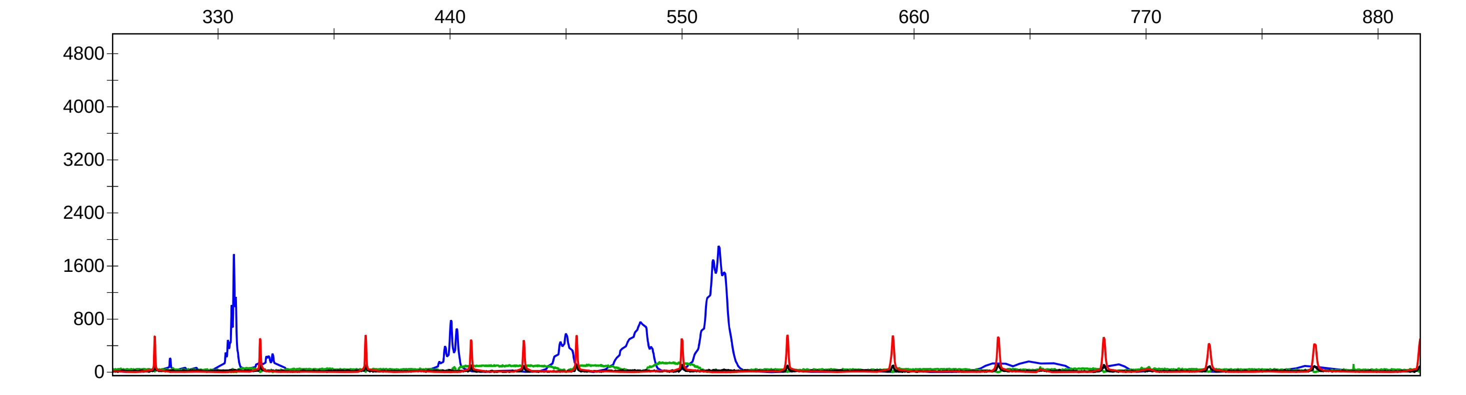

Sample File	Sample Name	Panel	OS	SQ
7-54-PM62_F07.fsa	7-54-PM62	R62_150-1500		

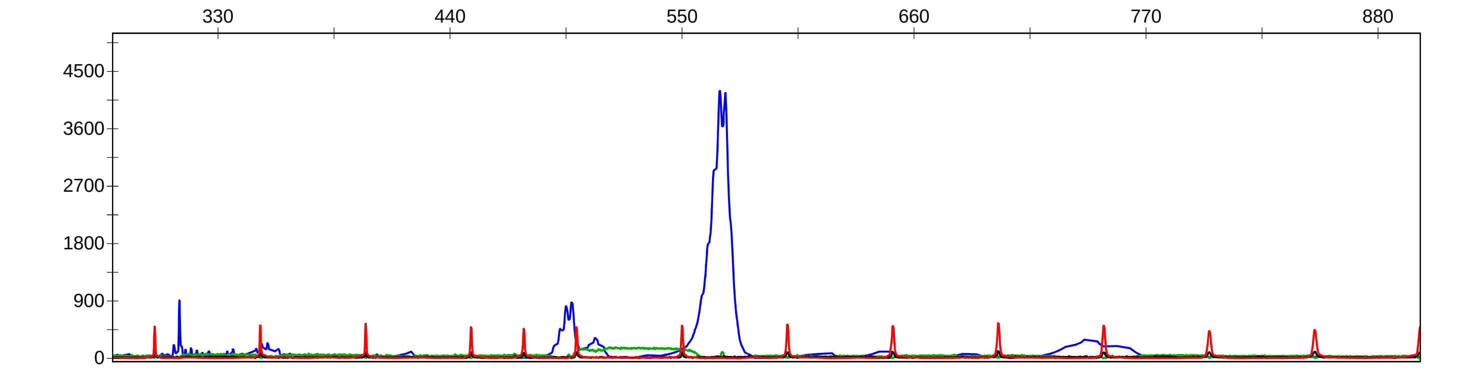

Sample File	Sample Name	Panel	OS	SQ
7-55-PM63_G07.fsa	7-55-PM63	R62_150-1500		

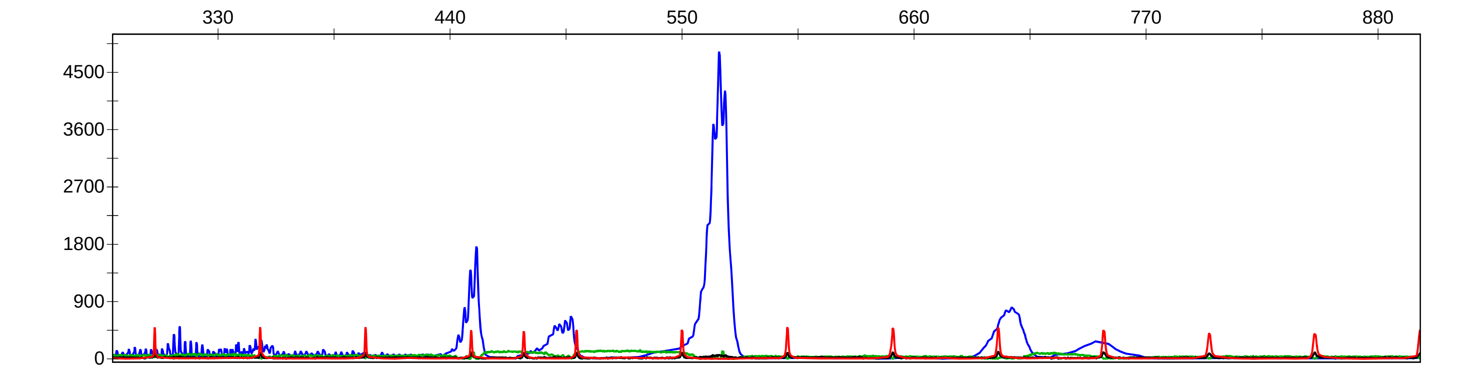

Sample File	Sample Name	Panel	OS	SQ
7-56-PM64_H07.fsa	7-56-PM64	R62_150-1500		

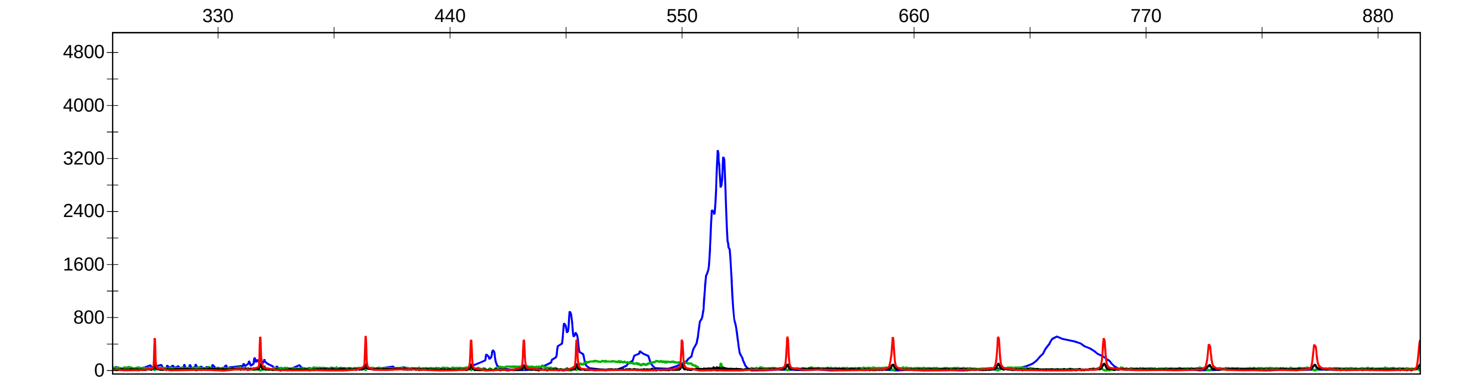

Sample File	Sample Name	Panel	OS	SQ
7-57-PM65_A08.fsa	7-57-PM65	R62_150-1500		

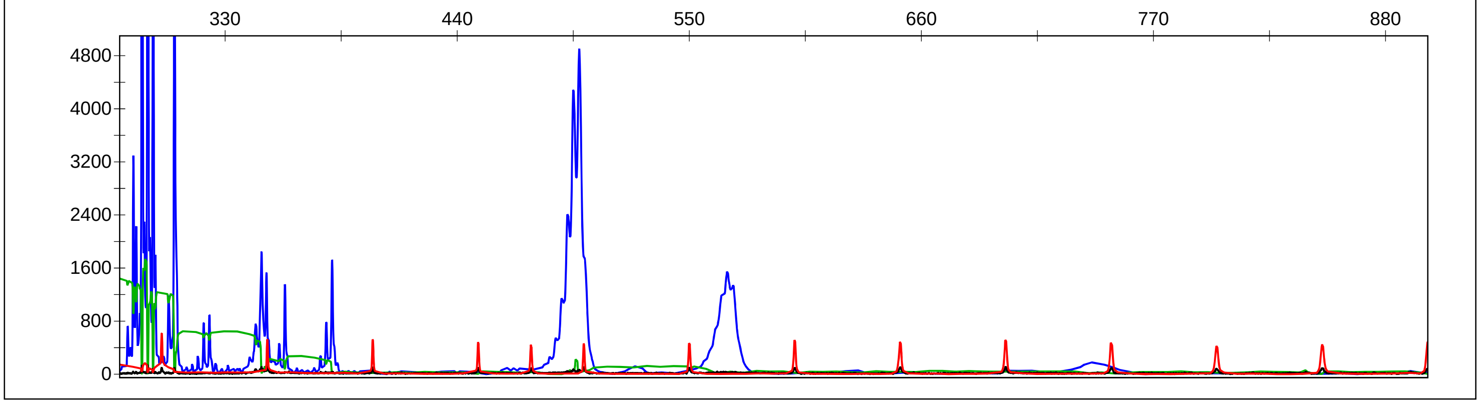

Sample File	Sample Name	Panel	OS	SQ
7-58-PM66_B08.fsa	7-58-PM66	R62_150-1500		

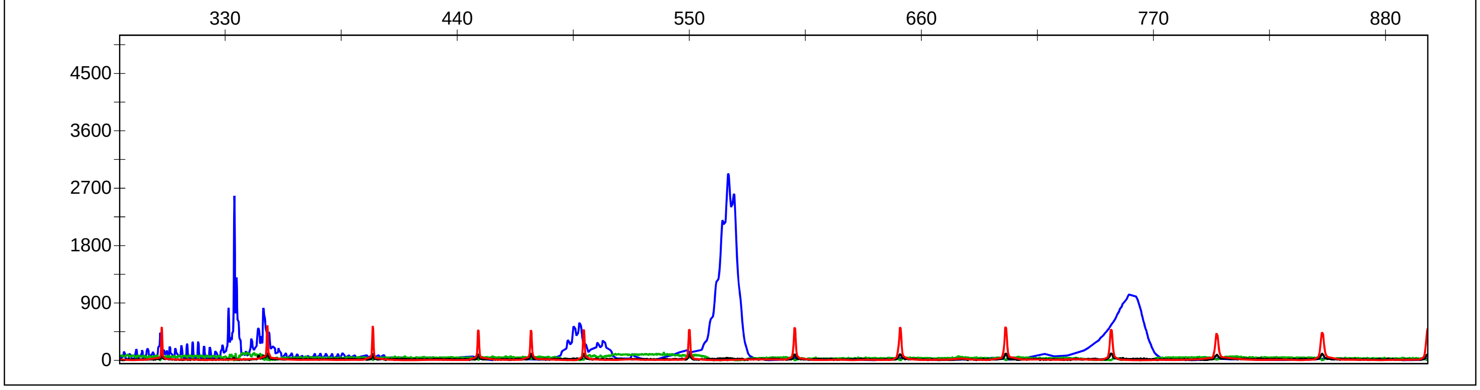

Sample File	Sample Name	Panel	OS	SQ
7-59-PM67_C08.fsa	7-59-PM67	R62_150-1500		

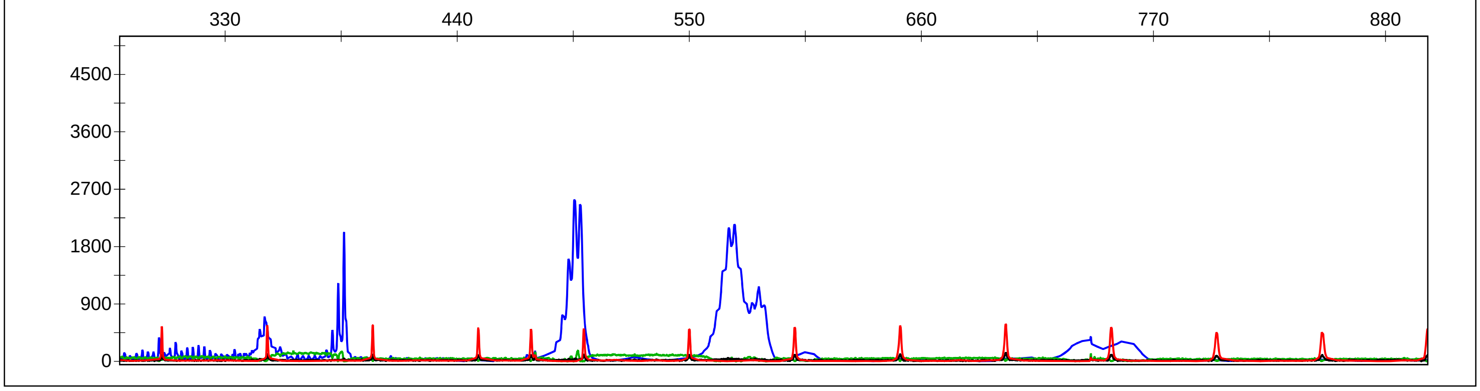

Sample File	Sample Name	Panel	OS	SQ
7-60-PM68_D08.fsa	7-60-PM68	R62_150-1500		

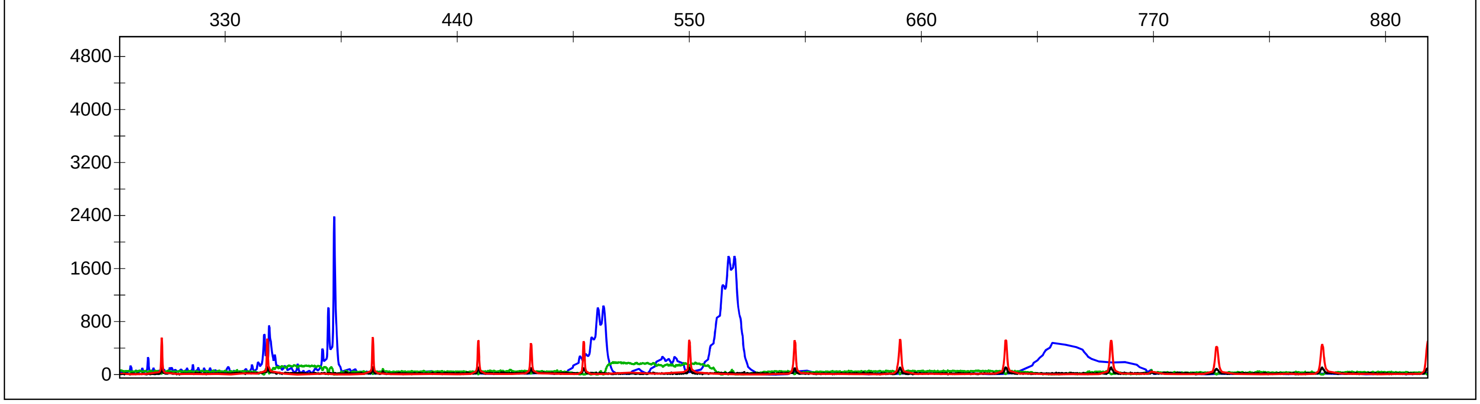

Sample File	Sample Name	Panel	OS	SQ
7-61-PM69_E08.fsa	7-61-PM69	R62_150-1500		

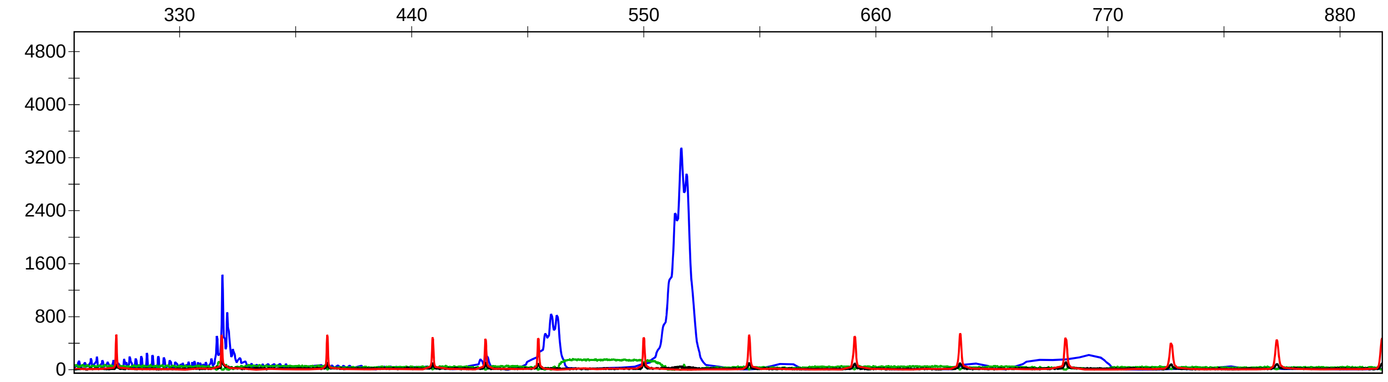

Sample File	Sample Name	Panel	OS	SQ
7-62-N2_F08.fsa	7-62-N2	R62_150-550		

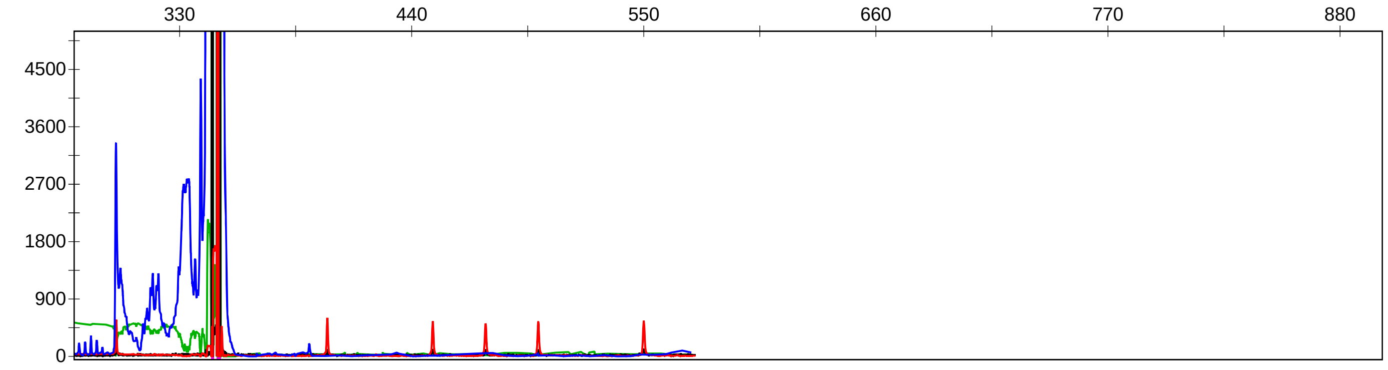

Sample File	Sample Name	Panel	OS	SQ
7-63-N4_G08.fsa	7-63-N4	R62_150-550		

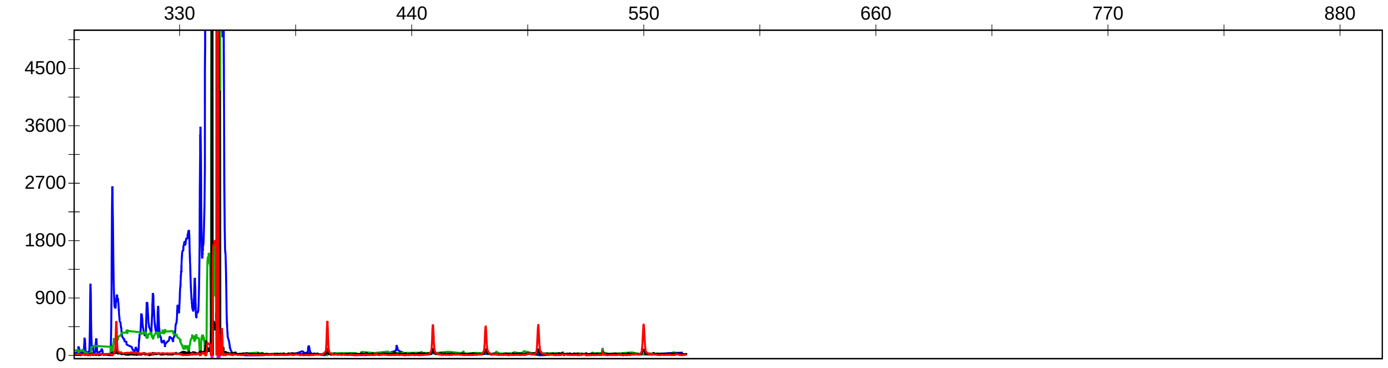

Sample File	Sample Name	Panel	OS	SQ
7-64-N5_H08.fsa	7-64-N5	R62_150-550		

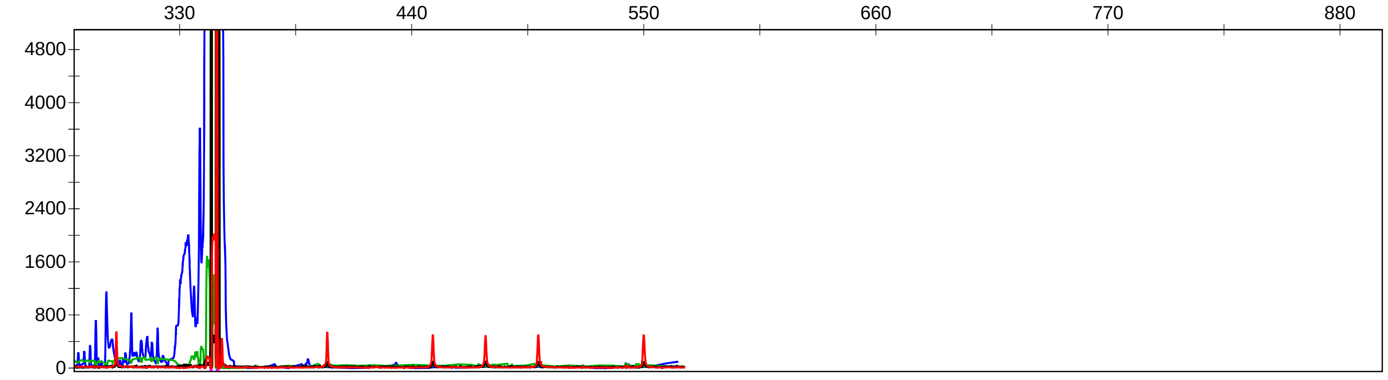

GeneMapper Software 5

20180922CGG

Sample File	Sample Name	Panel	OS	SQ
7-65-N6_A09.fsa	7-65-N6	R62_150-550		

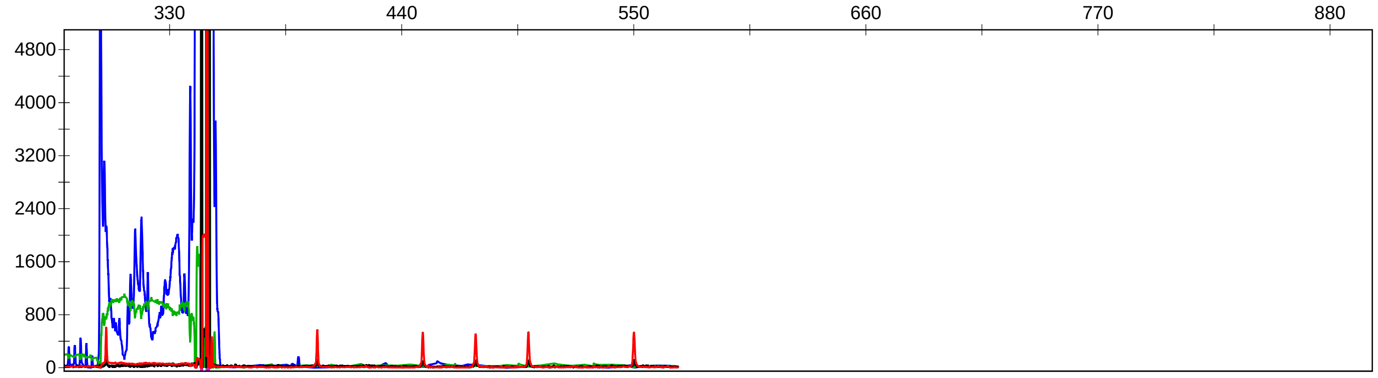

Sample File	Sample Name	Panel	OS	SQ
7-66-N10_B09.fsa	7-66-N10	R62_150-550		

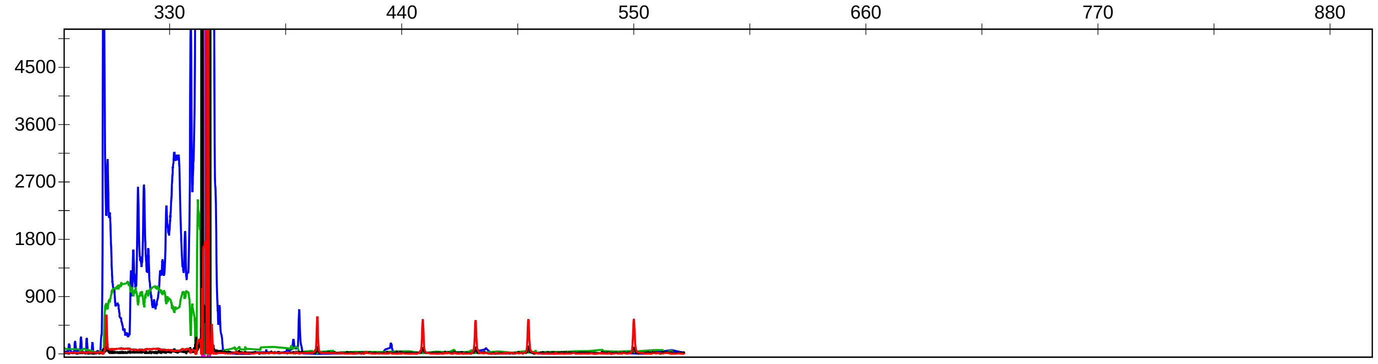

Sample File	Sample Name	Panel	OS	SQ
7-67-N13_C09.fsa	7-67-N13	R62_150-550		

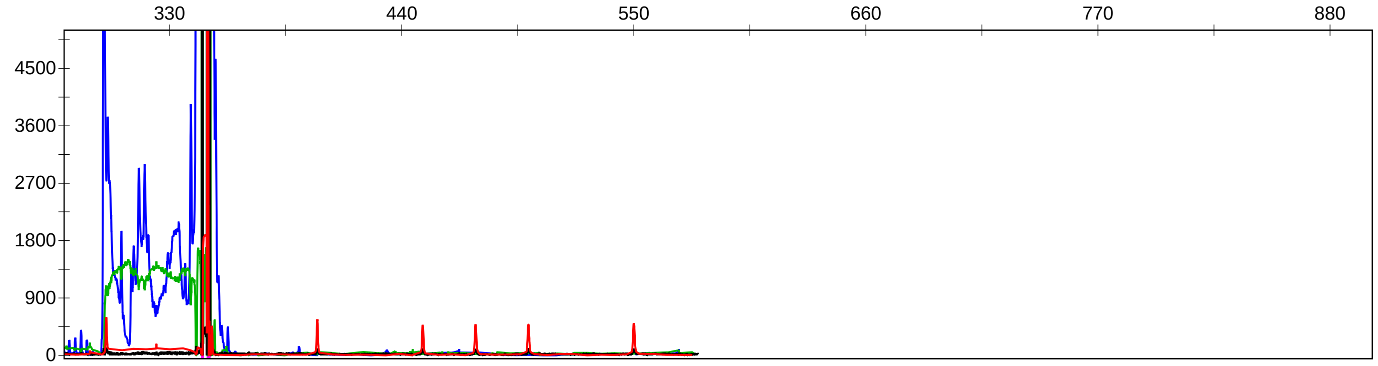

Sample File	Sample Name	Panel	OS	SQ
7-68-N14_D09.fsa	7-68-N14	R62_150-550		

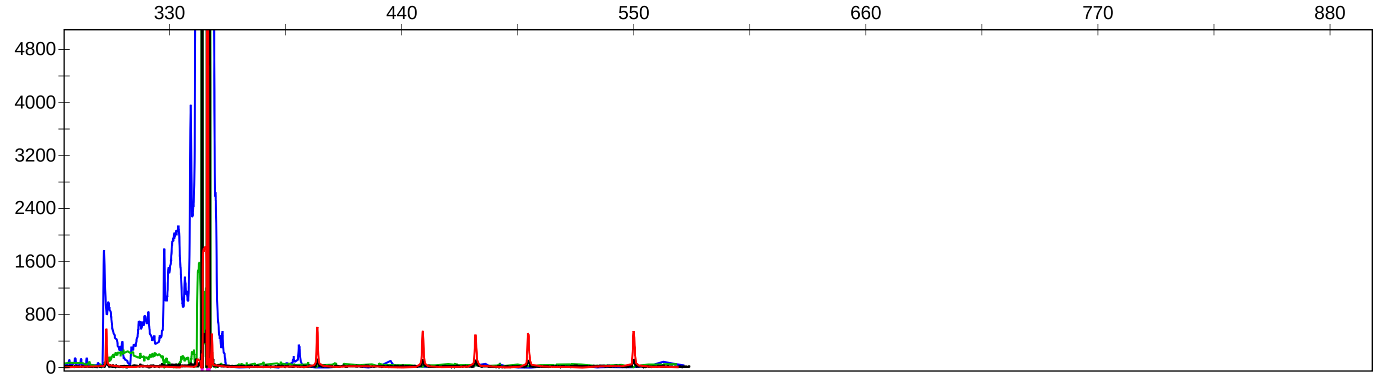

Sample File	Sample Name	Panel	OS	SQ
7-69-N16_E09.fsa	7-69-N16	R62_150-550		

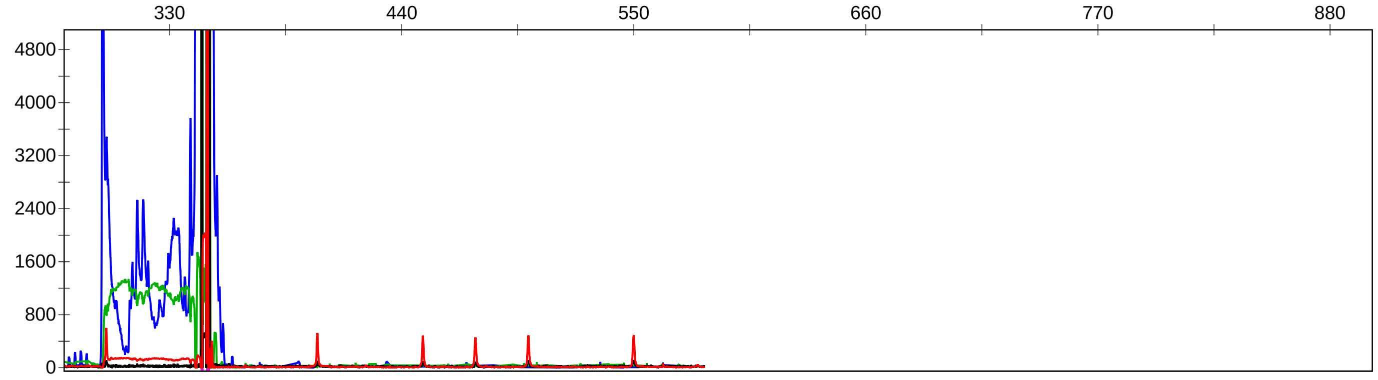

Sample File	Sample Name	Panel	OS	SQ
7-70-N17_F09.fsa	7-70-N17	R62_150-550		

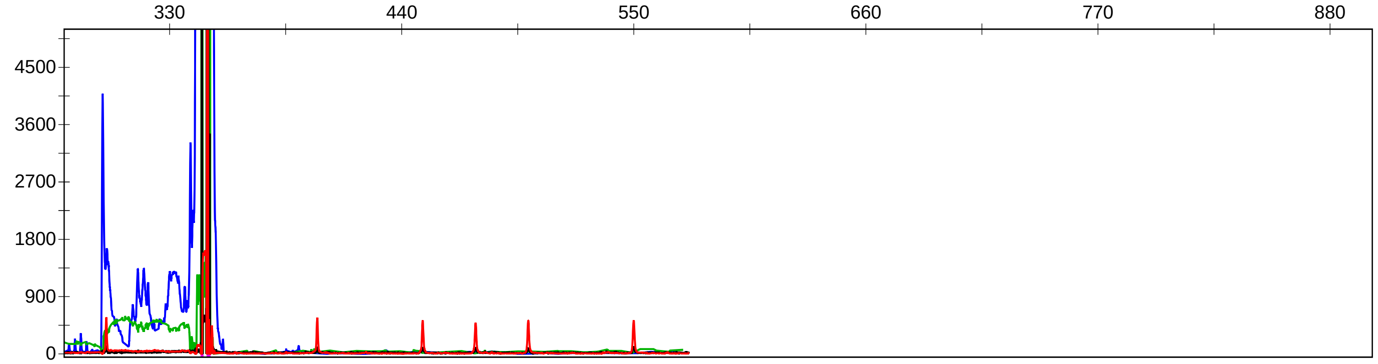

Sample File	Sample Name	Panel	OS	SQ
7-71-N18_G09.fsa	7-71-N18	R62_150-550		

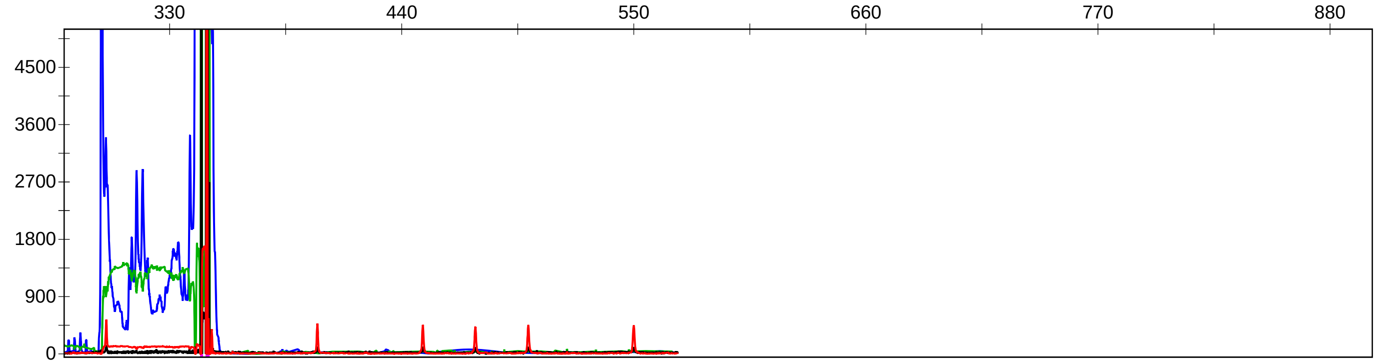

Sample File	Sample Name	Panel	OS	SQ
7-72-N19_H09.fsa	7-72-N19	R62_150-550		

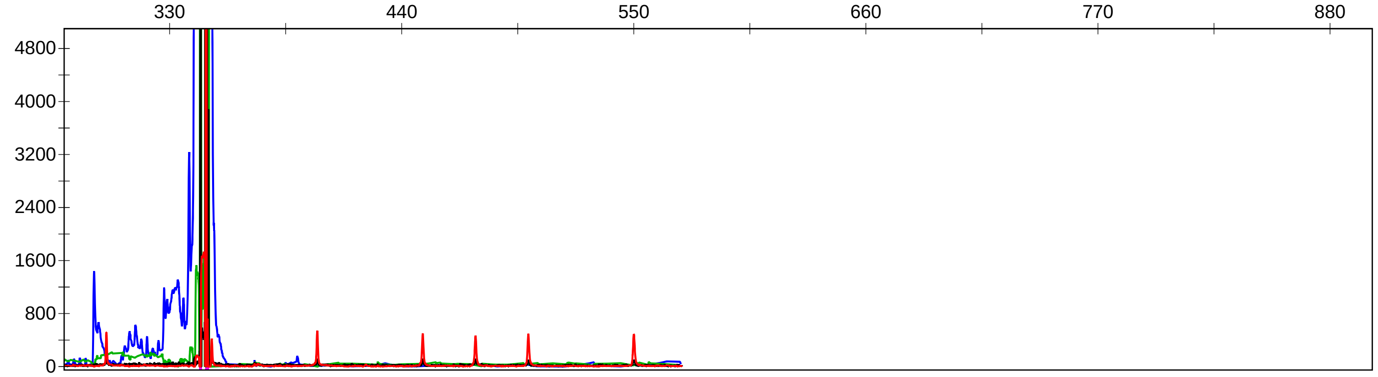

Sample File	Sample Name	Panel	OS	SQ
7-73-N20_A10.fsa	7-73-N20	R62_150-550		

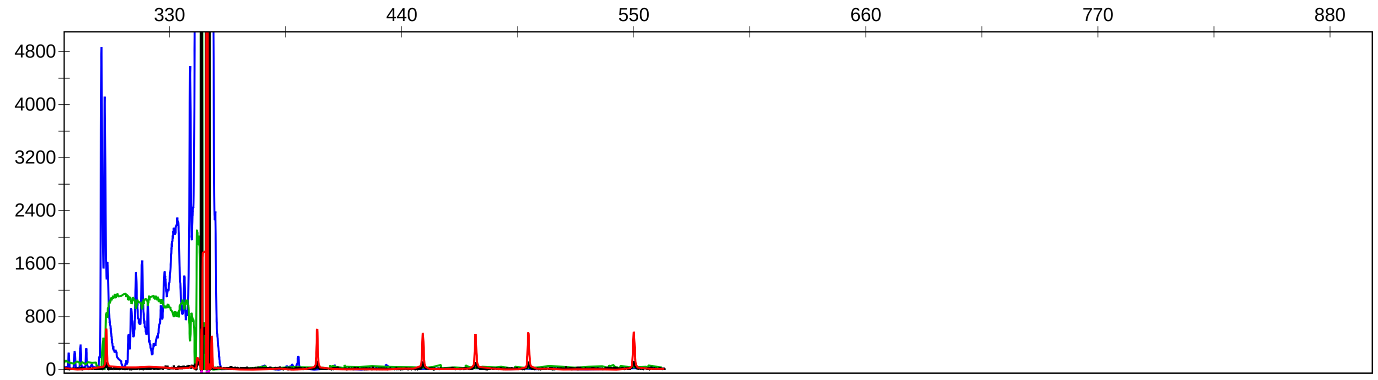

Sample File	Sample Name	Panel	OS	SQ
7-74-N23_B10.fsa	7-74-N23	R62_150-550		

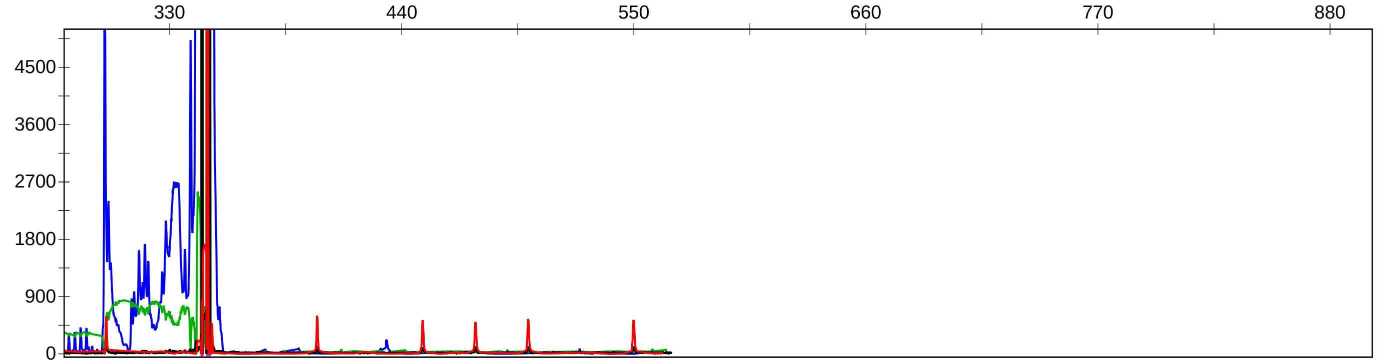

Sample File	Sample Name	Panel	OS	SQ
7-75-N24_C10.fsa	7-75-N24	R62_150-550		

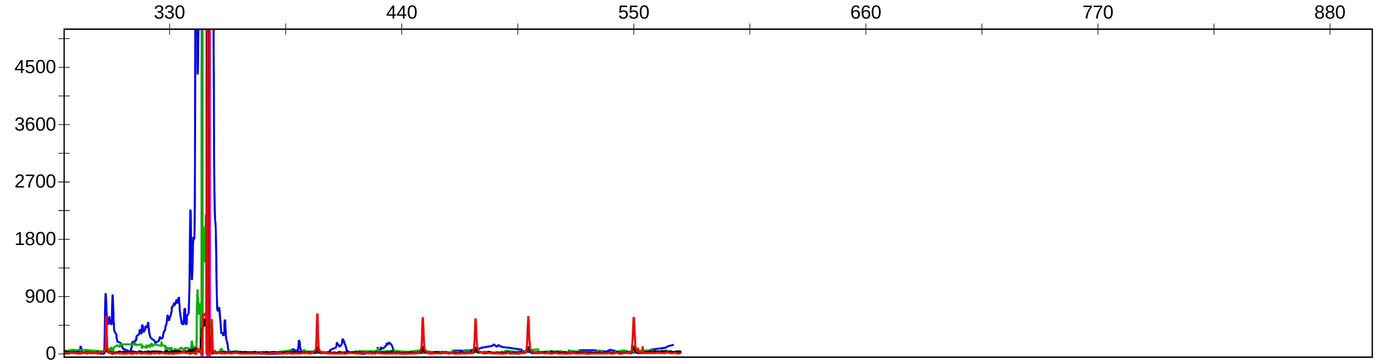

Sample File	Sample Name	Panel	OS	SQ
7-76-N25_D10.fsa	7-76-N25	R62_150-550		

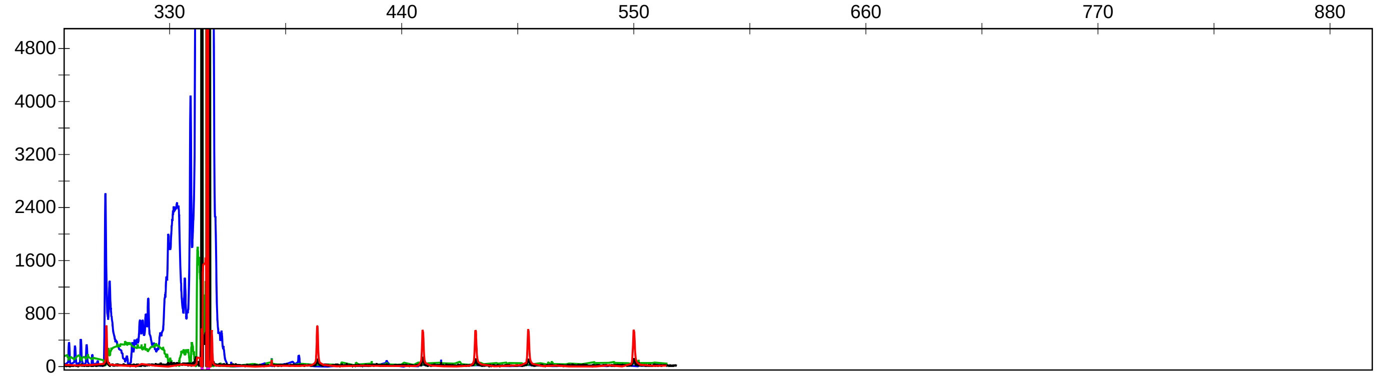

Sample File	Sample Name	Panel	OS	SQ
7-77-N28_E10.fsa	7-77-N28	R62_150-550		

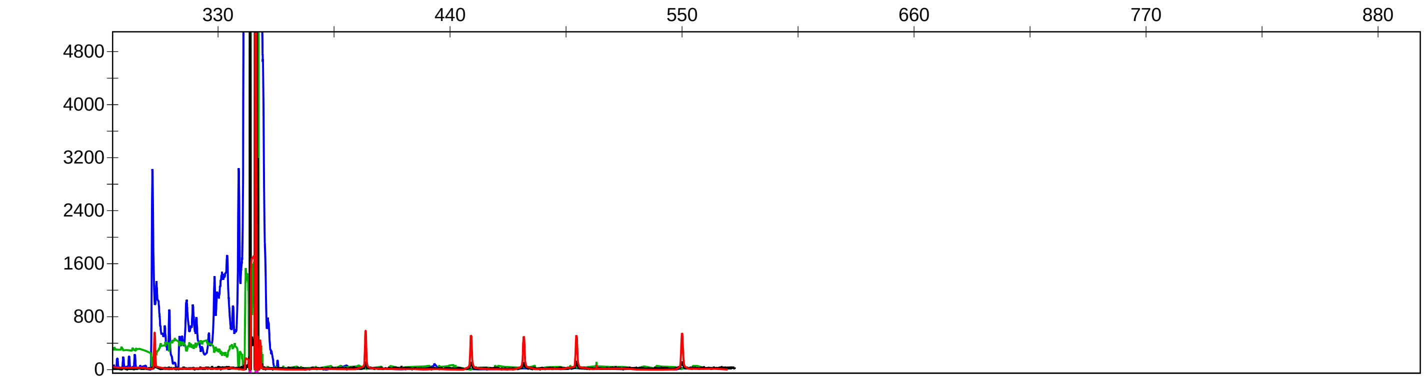

Sample File	Sample Name	Panel	OS	SQ
7-78-N29_F10.fsa	7-78-N29	R62_150-550		

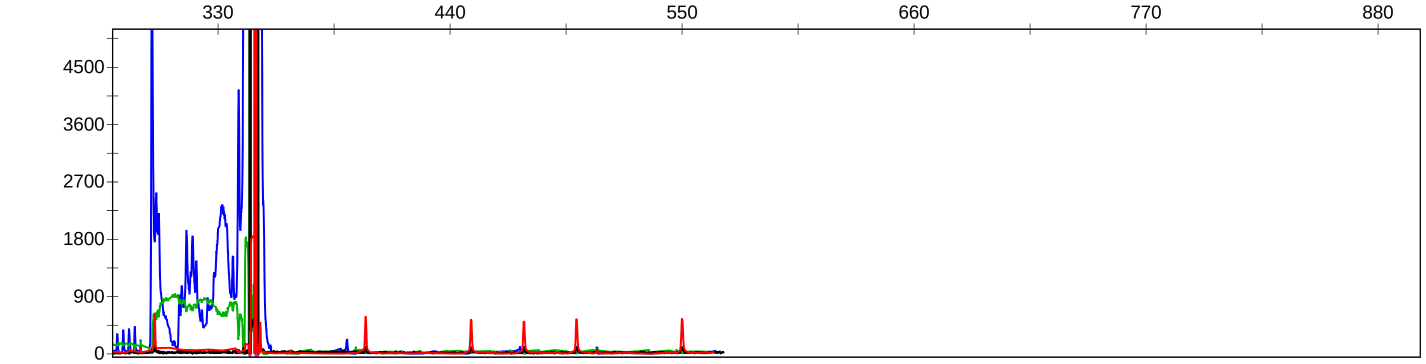

Sample File	Sample Name	Panel	OS	SQ
7-79-N31_G10.fsa	7-79-N31	R62_150-550		

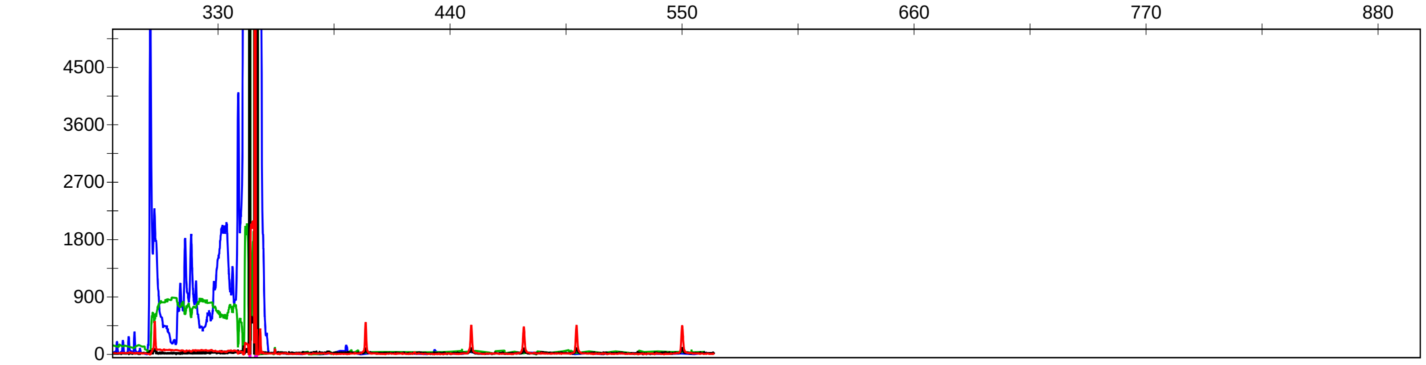

Sample File	Sample Name	Panel	OS	SQ
7-80-N32_H10.fsa	7-80-N32	R62_150-550		

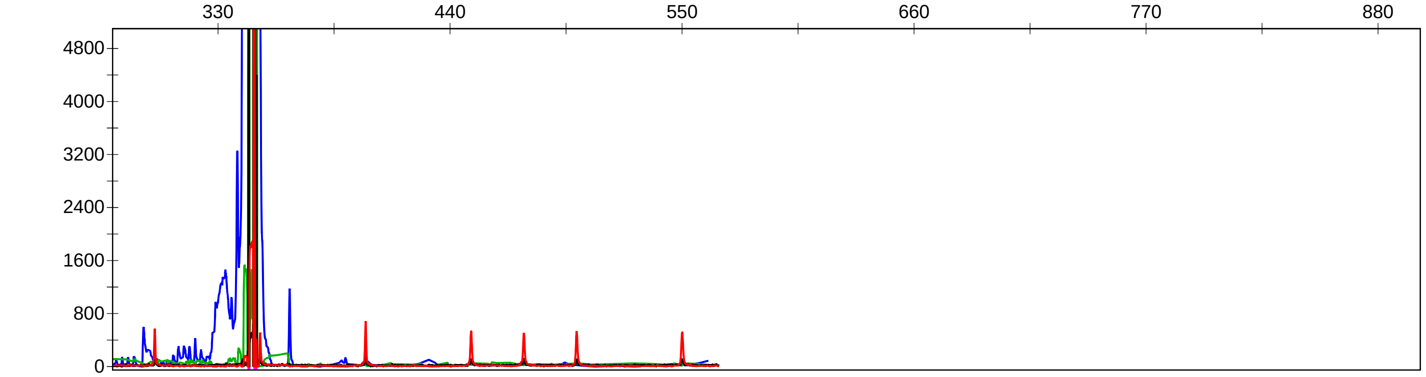

Sample File	Sample Name	Panel	OS	SQ
7-81-N33_A11.fsa	7-81-N33	R62_150-550		

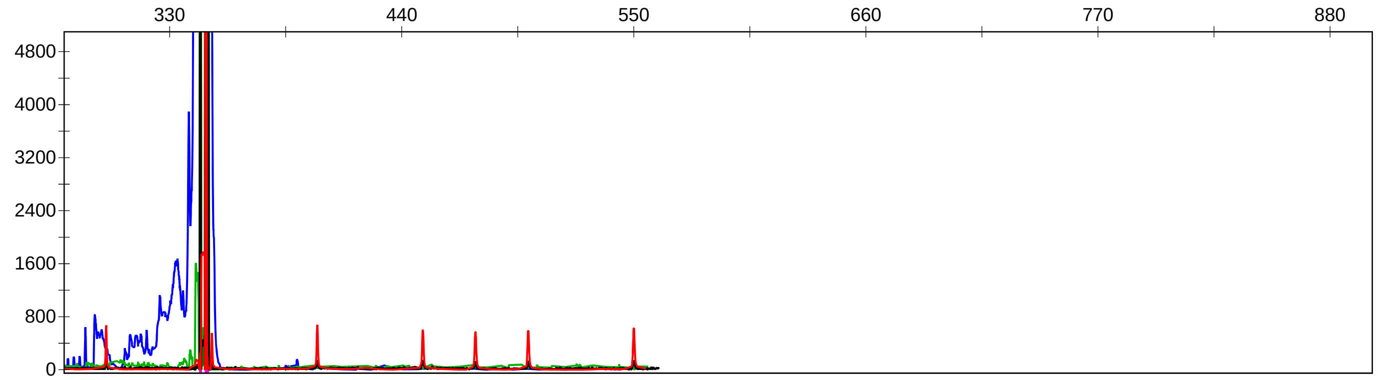

Sample File	Sample Name	Panel	OS	SQ
7-82-N34_B11.fsa	7-82-N34	R62_150-550		

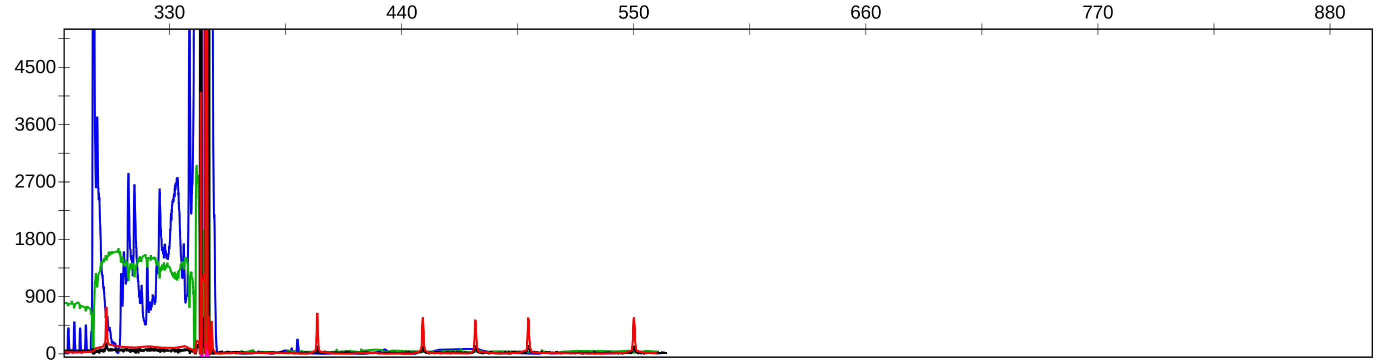

Sample File	Sample Name	Panel	OS	SQ
7-83-N41_C11.fsa	7-83-N41	R62_150-550		

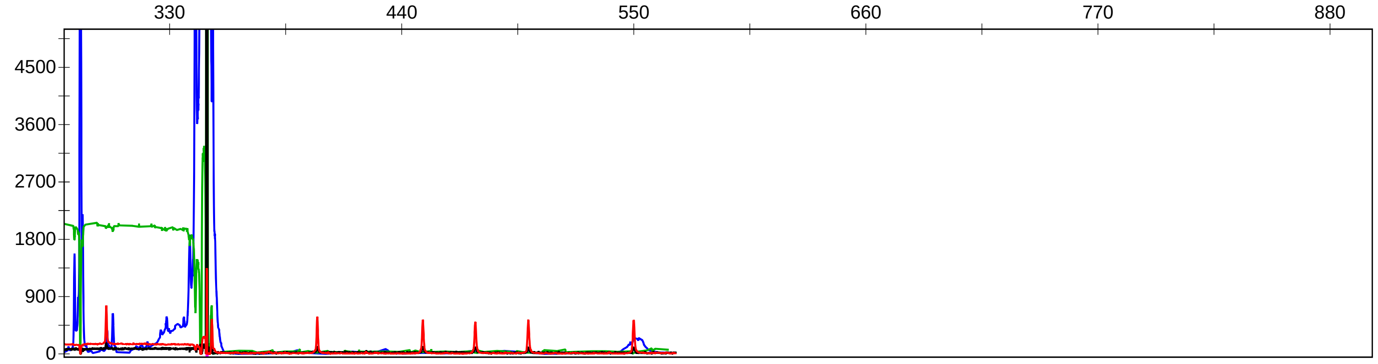

Sample File	Sample Name	Panel	OS	SQ
7-84-N43_D11.fsa	7-84-N43	R62_150-550		

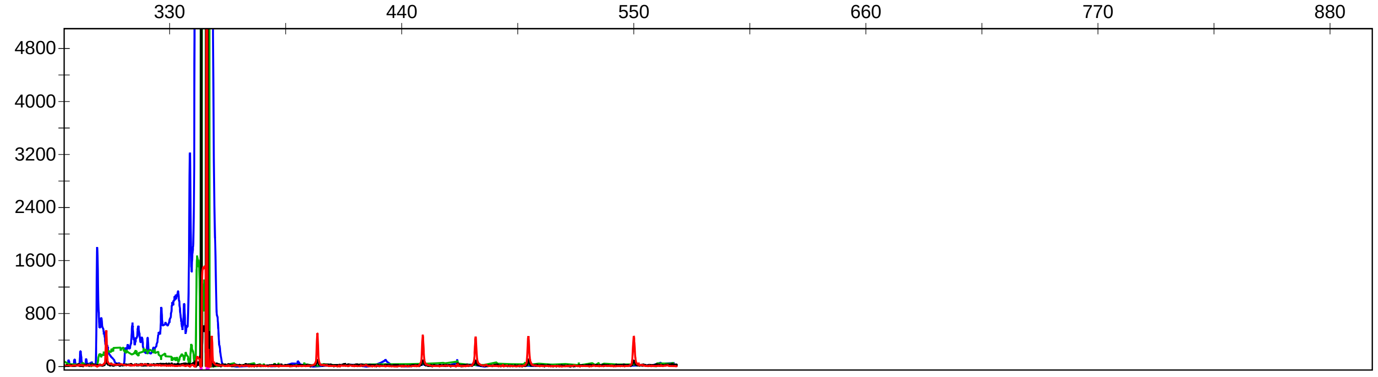

Sample File	Sample Name	Panel	OS	SQ
7-85-N44_E11.fsa	7-85-N44	R62_150-550		

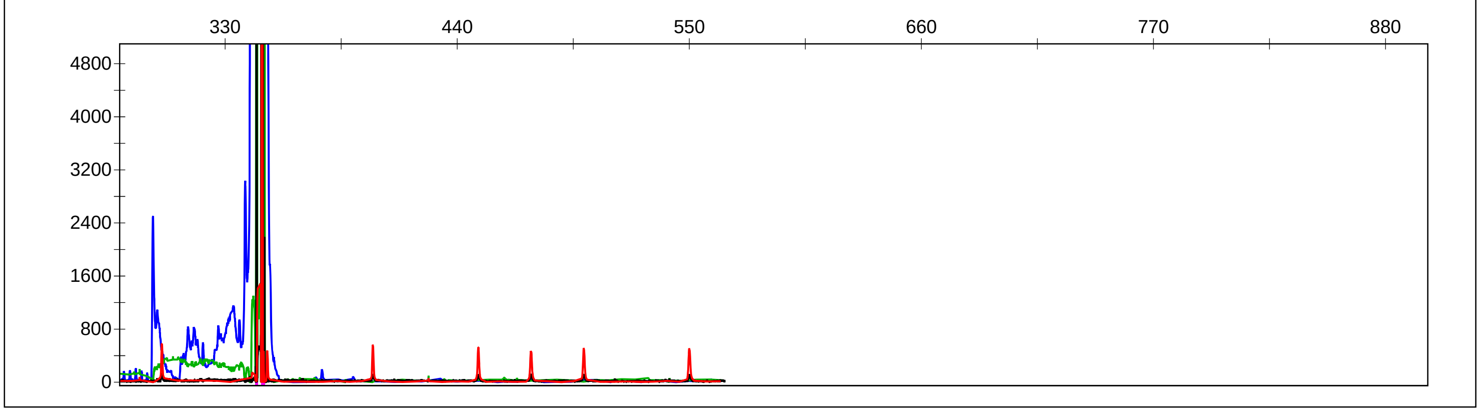

Sample File	Sample Name	Panel	OS	SQ
7-86-N46_F11.fsa	7-86-N46	R62_150-550		

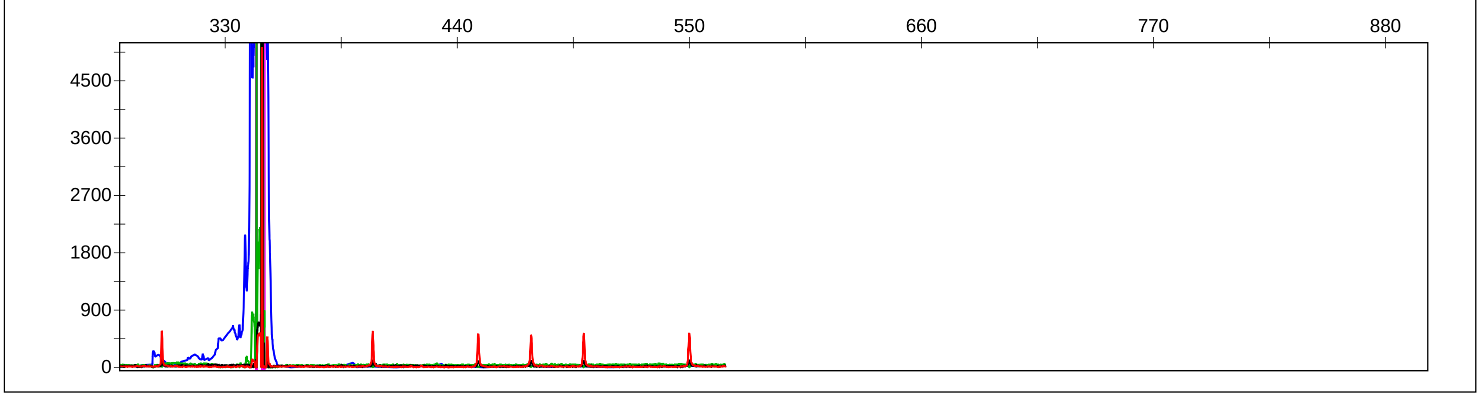

Sample File	Sample Name	Panel	OS	SQ
7-87-N48_G11.fsa	7-87-N48	R62_150-550		

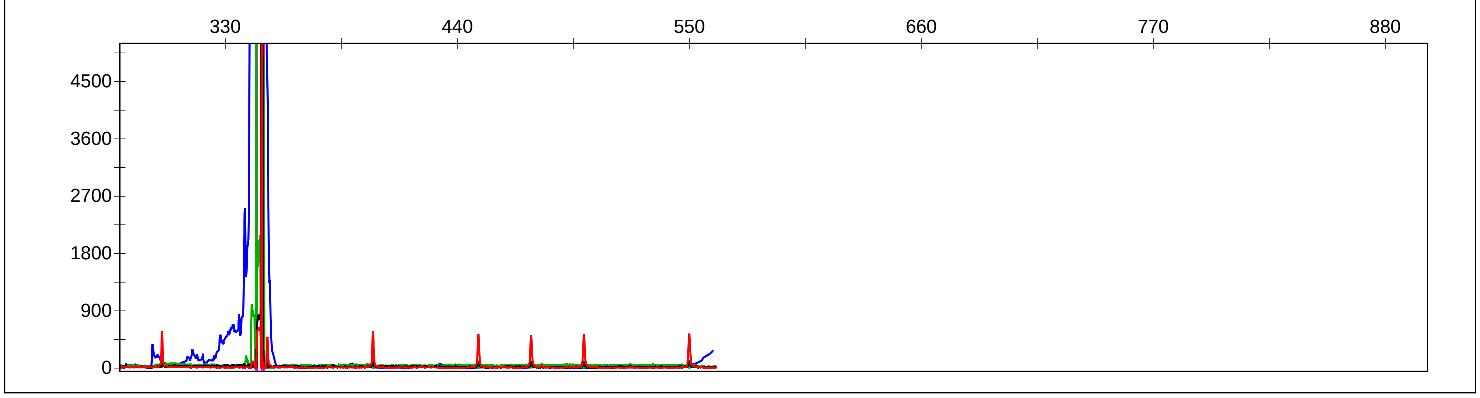

Sample File	Sample Name	Panel	OS	SQ
7-88-N49_H11.fsa	7-88-N49	R62_150-550		

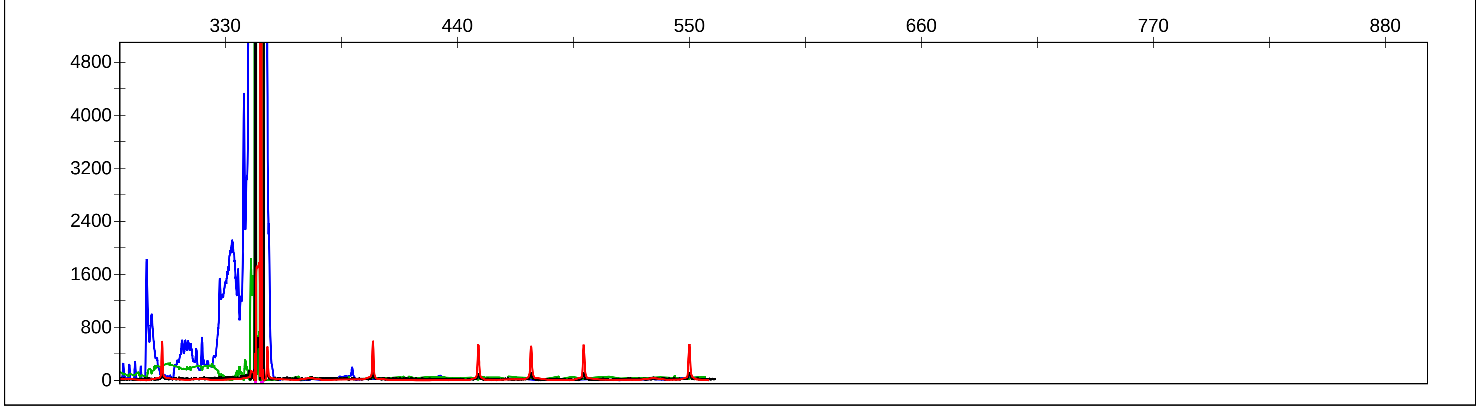

Sample File	Sample Name	Panel	OS	SQ
7-89-N51_A12.fsa	7-89-N51	R62_150-550		

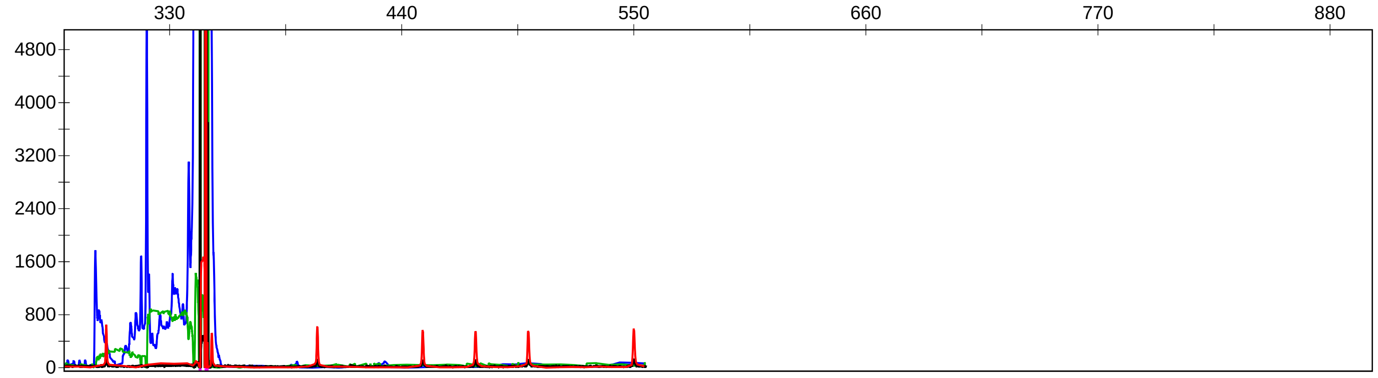

Sample File	Sample Name	Panel	OS	SQ
7-90-N52_B12.fsa	7-90-N52	R62_150-550		

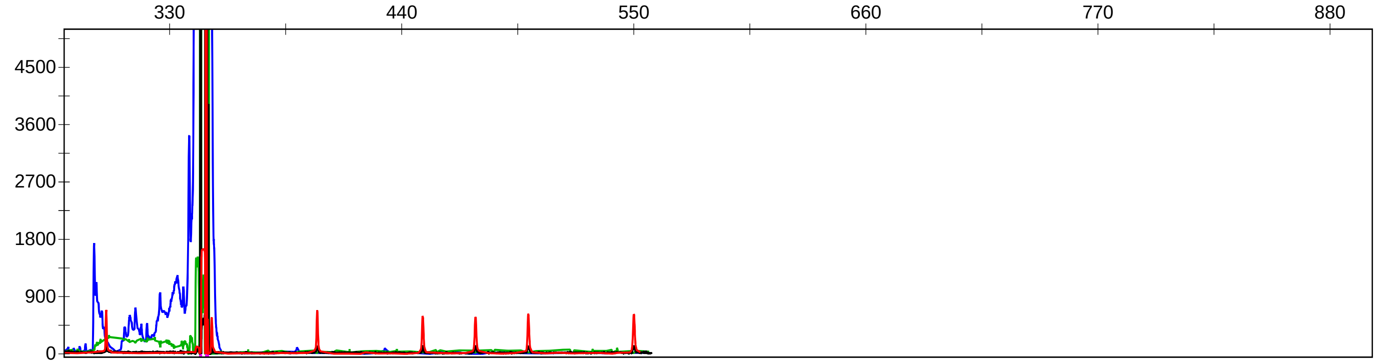

Sample File	Sample Name	Panel	OS	SQ
7-91-N54_C12.fsa	7-91-N54	R62_150-550		

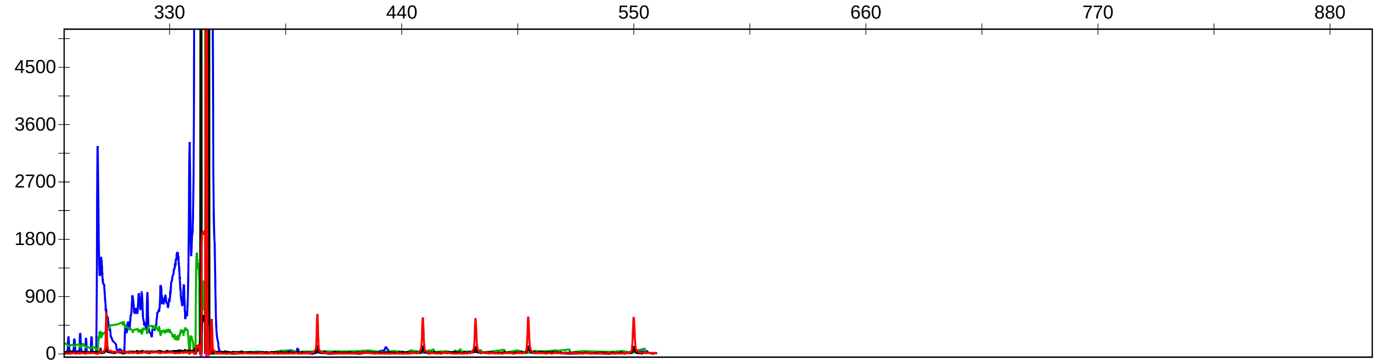

Sample File	Sample Name	Panel	OS	SQ
7-92-N57_D12.fsa	7-92-N57	R62_150-550		

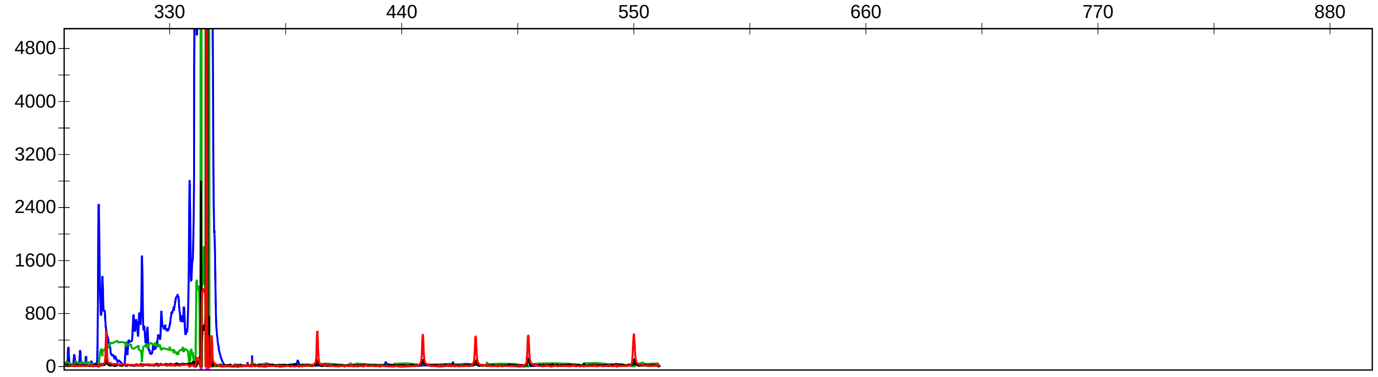

Sample File	Sample Name	Panel	OS	SQ
7-93-N60_E12.fsa	7-93-N60	R62_150-550		

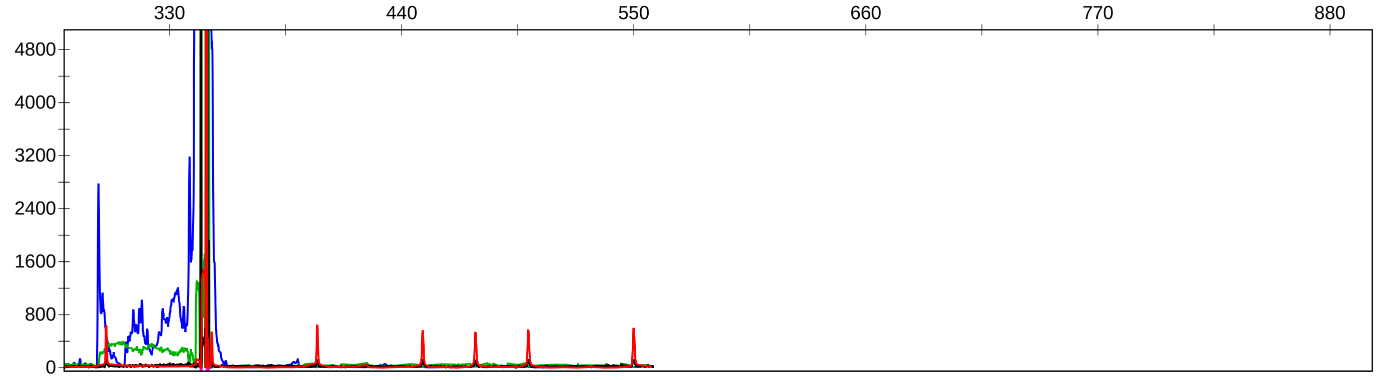

7-94-N67_F12.fsa	7-94-N67	R62_150-550		

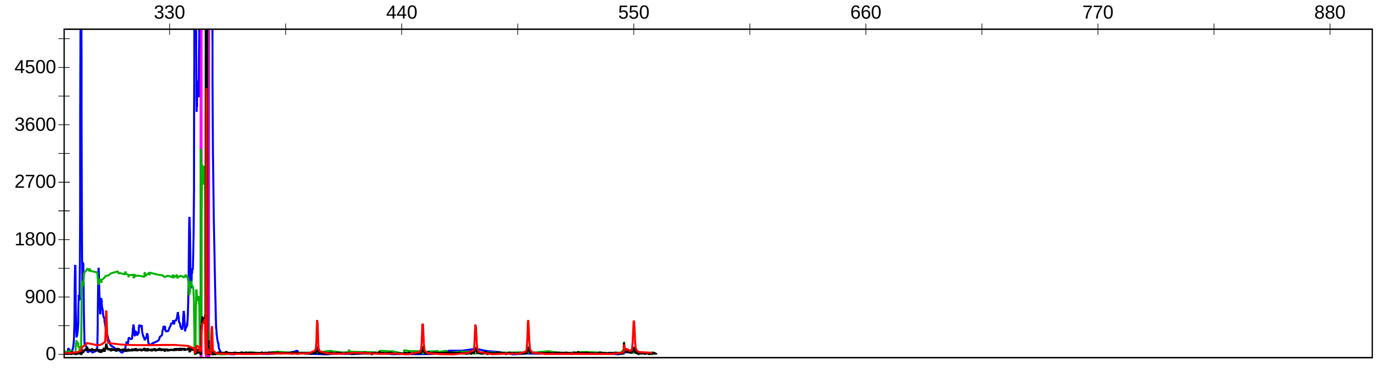

7-95-N68_G12.fsa	7-95-N68	R62_150-550		

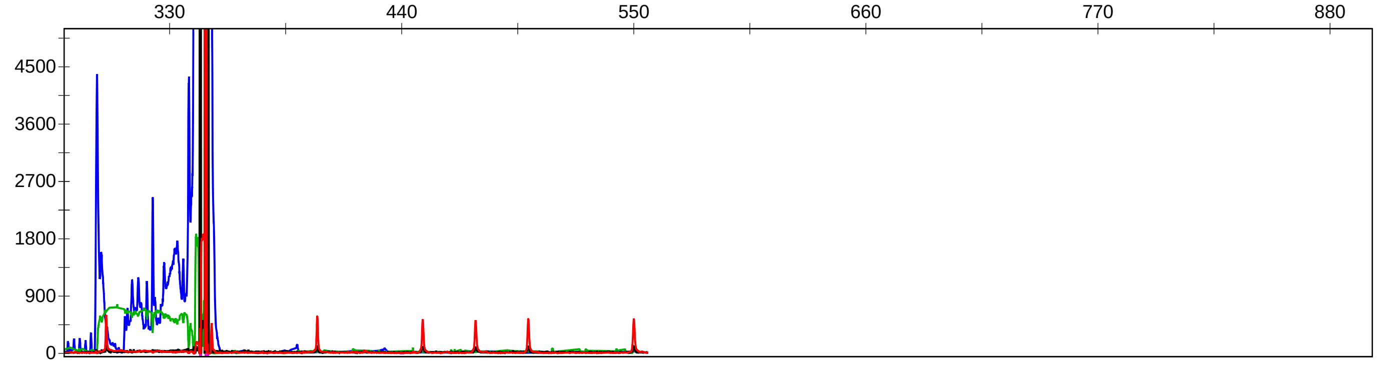

7-96-R62_H12.fsa	7-96-R62	R62_150-550		

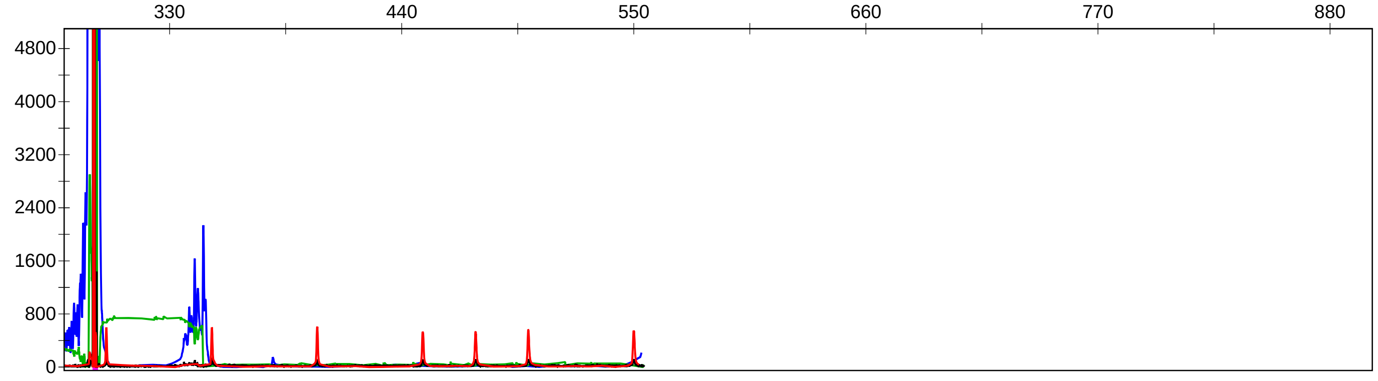

Supplement: Supplementary File [file pnas.1808377115.sd01.pdf]
